# Supplementary material for: Genome-wide identification, characterization and gene expression of BES1 transcription factor family in grapevine (Vitis vinifera L.)
Source: Sci Rep. 2023 Jan 5;13:240. doi: 10.1038/s41598-022-24407-y (PMC9816167; doi:10.1038/s41598-022-24407-y)
Supplement: Supplementary file 3 — Supplementary Information. [file 41598_2022_24407_MOESM3_ESM.zip › Vvi_Atr/Vitis_vinifera.PN40024.v4.dna_sm.toplevel.fa.vs.Amborella_trichopoda.AMTR1.0.dna_sm.toplevel.fa.html/Vvi-8.html]

|  |  |  |  |  |  |  |  |  |  |  |  |  |  |
| --- | --- | --- | --- | --- | --- | --- | --- | --- | --- | --- | --- | --- | --- |
| Duplication depth | Reference chromosome | Collinear blocks | | | | | | | | | | | |
| 0 | Vvi-Vitvi08g04000\_t001 |  |  |  |  |  |  |  |  |  |  |  |  |
| 0 | Vvi-Vitvi08g04001\_t001 |  |  |  |  |  |  |  |  |  |  |  |  |
| 0 | Vvi-Vitvi08g00004\_t002 |  |  |  |  |  |  |  |  |  |  |  |  |
| 0 | Vvi-Vitvi08g00005\_t001 |  |  |  |  |  |  |  |  |  |  |  |  |
| 1 | Vvi-Vitvi08g04002\_t001 |  | Atr-ERN04319 |  |  |  |  |  |  |  |  |  |  |
| 1 | Vvi-Vitvi08g04003\_t001 |  | \| \| |  |  |  |  |  |  |  |  |  |  |
| 1 | Vvi-Vitvi08g00008\_t001 |  | Atr-ERN04315 |  |  |  |  |  |  |  |  |  |  |
| 1 | Vvi-Vitvi08g00009\_t001 |  | \| \| |  |  |  |  |  |  |  |  |  |  |
| 1 | Vvi-Vitvi08g00010\_t001 |  | \| \| |  |  |  |  |  |  |  |  |  |  |
| 1 | Vvi-Vitvi08g00011\_t001 |  | \| \| |  |  |  |  |  |  |  |  |  |  |
| 1 | Vvi-Vitvi08g00012\_t001 |  | Atr-ERN04314 |  |  |  |  |  |  |  |  |  |  |
| 1 | Vvi-Vitvi08g00013\_t002 |  | Atr-ERN04313 |  |  |  |  |  |  |  |  |  |  |
| 1 | Vvi-Vitvi08g00014\_t001 |  | \| \| |  |  |  |  |  |  |  |  |  |  |
| 1 | Vvi-Vitvi08g01970\_t001 |  | Atr-ERN04308 |  |  |  |  |  |  |  |  |  |  |
| 1 | Vvi-Vitvi08g00015\_t001 |  | \| \| |  |  |  |  |  |  |  |  |  |  |
| 1 | Vvi-Vitvi08g01971\_t001 |  | \| \| |  |  |  |  |  |  |  |  |  |  |
| 1 | Vvi-Vitvi08g00016\_t001 |  | \| \| |  |  |  |  |  |  |  |  |  |  |
| 1 | Vvi-Vitvi08g00017\_t001 |  | \| \| |  |  |  |  |  |  |  |  |  |  |
| 1 | Vvi-Vitvi08g00018\_t001 |  | Atr-ERN04304 |  |  |  |  |  |  |  |  |  |  |
| 1 | Vvi-Vitvi08g00019\_t001 |  | Atr-ERN04302 |  |  |  |  |  |  |  |  |  |  |
| 1 | Vvi-Vitvi08g00020\_t001 |  | Atr-ERN04301 |  |  |  |  |  |  |  |  |  |  |
| 1 | Vvi-Vitvi08g00021\_t001 |  | Atr-ERN04299 |  |  |  |  |  |  |  |  |  |  |
| 1 | Vvi-Vitvi08g00022\_t001 |  | Atr-ERN04298 |  |  |  |  |  |  |  |  |  |  |
| 1 | Vvi-Vitvi08g00023\_t001 |  | Atr-ERN04297 |  |  |  |  |  |  |  |  |  |  |
| 1 | Vvi-Vitvi08g01973\_t001 |  | Atr-ERN04296 |  |  |  |  |  |  |  |  |  |  |
| 1 | Vvi-Vitvi08g00024\_t002 |  | \| \| |  |  |  |  |  |  |  |  |  |  |
| 1 | Vvi-Vitvi08g00026\_t001 |  | Atr-ERN04290 |  |  |  |  |  |  |  |  |  |  |
| 1 | Vvi-Vitvi08g01974\_t001 |  | \| \| |  |  |  |  |  |  |  |  |  |  |
| 1 | Vvi-Vitvi08g00027\_t001 |  | Atr-ERN04282 |  |  |  |  |  |  |  |  |  |  |
| 1 | Vvi-Vitvi08g00028\_t002 |  | Atr-ERN04278 |  |  |  |  |  |  |  |  |  |  |
| 1 | Vvi-Vitvi08g00030\_t001 |  | Atr-ERN04277 |  |  |  |  |  |  |  |  |  |  |
| 1 | Vvi-Vitvi08g04004\_t001 |  | \| \| |  |  |  |  |  |  |  |  |  |  |
| 1 | Vvi-Vitvi08g00033\_t001 |  | \| \| |  |  |  |  |  |  |  |  |  |  |
| 1 | Vvi-Vitvi08g04005\_t001 |  | \| \| |  |  |  |  |  |  |  |  |  |  |
| 1 | Vvi-Vitvi08g04006\_t001 |  | \| \| |  |  |  |  |  |  |  |  |  |  |
| 1 | Vvi-Vitvi08g04007\_t001 |  | \| \| |  |  |  |  |  |  |  |  |  |  |
| 1 | Vvi-Vitvi08g00037\_t001 |  | Atr-ERN04267 |  |  |  |  |  |  |  |  |  |  |
| 1 | Vvi-Vitvi08g00038\_t001 |  | Atr-ERN04265 |  |  |  |  |  |  |  |  |  |  |
| 1 | Vvi-Vitvi08g00039\_t001 |  | \| \| |  |  |  |  |  |  |  |  |  |  |
| 2 | Vvi-Vitvi08g00041\_t001 |  | Atr-ERN04263 |  | Atr-ERN04242 |  |  |  |  |  |  |  |  |
| 2 | Vvi-Vitvi08g00042\_t001 |  | \| \| |  | Atr-ERN04244 |  |  |  |  |  |  |  |  |
| 2 | Vvi-Vitvi08g04008\_t001 |  | \| \| |  | \| \| |  |  |  |  |  |  |  |  |
| 2 | Vvi-Vitvi08g00045\_t001 |  | \| \| |  | \| \| |  |  |  |  |  |  |  |  |
| 2 | Vvi-Vitvi08g00046\_t002 |  | \| \| |  | Atr-ERN04246 |  |  |  |  |  |  |  |  |
| 2 | Vvi-Vitvi08g04009\_t001 |  | \| \| |  | \| \| |  |  |  |  |  |  |  |  |
| 2 | Vvi-Vitvi08g01983\_t001 |  | \| \| |  | \| \| |  |  |  |  |  |  |  |  |
| 2 | Vvi-Vitvi08g00047\_t001 |  | Atr-ERN04249 |  | Atr-ERN04249 |  |  |  |  |  |  |  |  |
| 2 | Vvi-Vitvi08g00048\_t001 |  | \| \| |  | \| \| |  |  |  |  |  |  |  |  |
| 2 | Vvi-Vitvi08g04010\_t001 |  | \| \| |  | \| \| |  |  |  |  |  |  |  |  |
| 2 | Vvi-Vitvi08g00050\_t001 |  | \| \| |  | \| \| |  |  |  |  |  |  |  |  |
| 2 | Vvi-Vitvi08g00051\_t002 |  | \| \| |  | Atr-ERN04253 |  |  |  |  |  |  |  |  |
| 2 | Vvi-Vitvi08g00052\_t001 |  | \| \| |  | Atr-ERN04254 |  |  |  |  |  |  |  |  |
| 2 | Vvi-Vitvi08g00053\_t001 |  | \| \| |  | Atr-ERN04256 |  |  |  |  |  |  |  |  |
| 2 | Vvi-Vitvi08g00054\_t001 |  | \| \| |  | Atr-ERN04259 |  |  |  |  |  |  |  |  |
| 1 | Vvi-Vitvi08g00055\_t001 |  | Atr-ERN04240 |  |  |  |  |  |  |  |  |  |  |
| 0 | Vvi-Vitvi08g04011\_t001 |  |  |  |  |  |  |  |  |  |  |  |  |
| 0 | Vvi-Vitvi08g00056\_t001 |  |  |  |  |  |  |  |  |  |  |  |  |
| 0 | Vvi-Vitvi08g01987\_t005 |  |  |  |  |  |  |  |  |  |  |  |  |
| 0 | Vvi-Vitvi08g00058\_t001 |  |  |  |  |  |  |  |  |  |  |  |  |
| 0 | Vvi-Vitvi08g00059\_t001 |  |  |  |  |  |  |  |  |  |  |  |  |
| 0 | Vvi-Vitvi08g00060\_t001 |  |  |  |  |  |  |  |  |  |  |  |  |
| 0 | Vvi-Vitvi08g00061\_t002 |  |  |  |  |  |  |  |  |  |  |  |  |
| 0 | Vvi-Vitvi08g00062\_t002 |  |  |  |  |  |  |  |  |  |  |  |  |
| 0 | Vvi-Vitvi08g01988\_t001 |  |  |  |  |  |  |  |  |  |  |  |  |
| 0 | Vvi-Vitvi08g00064\_t001 |  |  |  |  |  |  |  |  |  |  |  |  |
| 0 | Vvi-Vitvi08g04012\_t001 |  |  |  |  |  |  |  |  |  |  |  |  |
| 0 | Vvi-Vitvi08g00067\_t001 |  |  |  |  |  |  |  |  |  |  |  |  |
| 0 | Vvi-Vitvi08g00068\_t002 |  |  |  |  |  |  |  |  |  |  |  |  |
| 0 | Vvi-Vitvi08g00069\_t001 |  |  |  |  |  |  |  |  |  |  |  |  |
| 1 | Vvi-Vitvi08g00070\_t001 |  | Atr-ERM97282 |  |  |  |  |  |  |  |  |  |  |
| 1 | Vvi-Vitvi08g00071\_t001 |  | \| \| |  |  |  |  |  |  |  |  |  |  |
| 1 | Vvi-Vitvi08g00072\_t001 |  | \| \| |  |  |  |  |  |  |  |  |  |  |
| 1 | Vvi-Vitvi08g04013\_t001 |  | \| \| |  |  |  |  |  |  |  |  |  |  |
| 1 | Vvi-Vitvi08g04014\_t001 |  | \| \| |  |  |  |  |  |  |  |  |  |  |
| 1 | Vvi-Vitvi08g04015\_t001 |  | \| \| |  |  |  |  |  |  |  |  |  |  |
| 1 | Vvi-Vitvi08g00076\_t001 |  | \| \| |  |  |  |  |  |  |  |  |  |  |
| 1 | Vvi-Vitvi08g01990\_t001 |  | \| \| |  |  |  |  |  |  |  |  |  |  |
| 1 | Vvi-Vitvi08g04016\_t001 |  | \| \| |  |  |  |  |  |  |  |  |  |  |
| 1 | Vvi-Vitvi08g00078\_t001 |  | \| \| |  |  |  |  |  |  |  |  |  |  |
| 1 | Vvi-Vitvi08g04017\_t001 |  | \| \| |  |  |  |  |  |  |  |  |  |  |
| 1 | Vvi-Vitvi08g00079\_t001 |  | Atr-ERM97280 |  |  |  |  |  |  |  |  |  |  |
| 1 | Vvi-Vitvi08g00083\_t001 |  | Atr-ERM97257 |  |  |  |  |  |  |  |  |  |  |
| 1 | Vvi-Vitvi08g00085\_t002 |  | \| \| |  |  |  |  |  |  |  |  |  |  |
| 1 | Vvi-Vitvi08g01991\_t001 |  | \| \| |  |  |  |  |  |  |  |  |  |  |
| 1 | Vvi-Vitvi08g04018\_t001 |  | \| \| |  |  |  |  |  |  |  |  |  |  |
| 1 | Vvi-Vitvi08g00086\_t001 |  | \| \| |  |  |  |  |  |  |  |  |  |  |
| 1 | Vvi-Vitvi08g04019\_t001 |  | \| \| |  |  |  |  |  |  |  |  |  |  |
| 1 | Vvi-Vitvi08g00090\_t001 |  | \| \| |  |  |  |  |  |  |  |  |  |  |
| 1 | Vvi-Vitvi08g04020\_t001 |  | \| \| |  |  |  |  |  |  |  |  |  |  |
| 1 | Vvi-Vitvi08g00091\_t001 |  | \| \| |  |  |  |  |  |  |  |  |  |  |
| 1 | Vvi-Vitvi08g04021\_t001 |  | \| \| |  |  |  |  |  |  |  |  |  |  |
| 1 | Vvi-Vitvi08g00092\_t001 |  | \| \| |  |  |  |  |  |  |  |  |  |  |
| 1 | Vvi-Vitvi08g00093\_t001 |  | \| \| |  |  |  |  |  |  |  |  |  |  |
| 1 | Vvi-Vitvi08g00094\_t001 |  | \| \| |  |  |  |  |  |  |  |  |  |  |
| 1 | Vvi-Vitvi08g04022\_t001 |  | \| \| |  |  |  |  |  |  |  |  |  |  |
| 1 | Vvi-Vitvi08g00095\_t001 |  | \| \| |  |  |  |  |  |  |  |  |  |  |
| 1 | Vvi-Vitvi08g00096\_t001 |  | \| \| |  |  |  |  |  |  |  |  |  |  |
| 1 | Vvi-Vitvi08g00097\_t001 |  | \| \| |  |  |  |  |  |  |  |  |  |  |
| 1 | Vvi-Vitvi08g00099\_t003 |  | \| \| |  |  |  |  |  |  |  |  |  |  |
| 1 | Vvi-Vitvi08g00100\_t001 |  | \| \| |  |  |  |  |  |  |  |  |  |  |
| 1 | Vvi-Vitvi08g00101\_t001 |  | \| \| |  |  |  |  |  |  |  |  |  |  |
| 1 | Vvi-Vitvi08g00102\_t001 |  | \| \| |  |  |  |  |  |  |  |  |  |  |
| 1 | Vvi-Vitvi08g00103\_t001 |  | \| \| |  |  |  |  |  |  |  |  |  |  |
| 1 | Vvi-Vitvi08g00104\_t001 |  | \| \| |  |  |  |  |  |  |  |  |  |  |
| 1 | Vvi-Vitvi08g00105\_t001 |  | Atr-ERM97256 |  |  |  |  |  |  |  |  |  |  |
| 1 | Vvi-Vitvi08g00107\_t001 |  | Atr-ERM97255 |  |  |  |  |  |  |  |  |  |  |
| 1 | Vvi-Vitvi08g00108\_t001 |  | Atr-ERM97252 |  |  |  |  |  |  |  |  |  |  |
| 1 | Vvi-Vitvi08g00110\_t001 |  | Atr-ERM97251 |  |  |  |  |  |  |  |  |  |  |
| 0 | Vvi-Vitvi08g01993\_t001 |  |  |  |  |  |  |  |  |  |  |  |  |
| 1 | Vvi-Vitvi08g00112\_t001 |  | Atr-ERM93559 |  |  |  |  |  |  |  |  |  |  |
| 1 | Vvi-Vitvi08g01994\_t001 |  | \| \| |  |  |  |  |  |  |  |  |  |  |
| 1 | Vvi-Vitvi08g00113\_t001 |  | Atr-ERM93570 |  |  |  |  |  |  |  |  |  |  |
| 2 | Vvi-Vitvi08g00114\_t001 |  | \| \| |  | Atr-ERM93585 |  |  |  |  |  |  |  |  |
| 2 | Vvi-Vitvi08g00115\_t001 |  | Atr-ERM93573 |  | \| \| |  |  |  |  |  |  |  |  |
| 2 | Vvi-Vitvi08g00117\_t001 |  | \| \| |  | \| \| |  |  |  |  |  |  |  |  |
| 2 | Vvi-Vitvi08g00118\_t001 |  | \| \| |  | \| \| |  |  |  |  |  |  |  |  |
| 2 | Vvi-Vitvi08g00120\_t001 |  | Atr-ERM93575 |  | \| \| |  |  |  |  |  |  |  |  |
| 2 | Vvi-Vitvi08g01995\_t001 |  | Atr-ERM93577 |  | \| \| |  |  |  |  |  |  |  |  |
| 2 | Vvi-Vitvi08g04023\_t001 |  | \| \| |  | \| \| |  |  |  |  |  |  |  |  |
| 2 | Vvi-Vitvi08g00125\_t001 |  | \| \| |  | \| \| |  |  |  |  |  |  |  |  |
| 2 | Vvi-Vitvi08g00126\_t001 |  | \| \| |  | \| \| |  |  |  |  |  |  |  |  |
| 3 | Vvi-Vitvi08g01999\_t001 |  | \| \| |  | \| \| |  | Atr-ERM93552 |  |  |  |  |  |  |
| 3 | Vvi-Vitvi08g00128\_t001 |  | \| \| |  | \| \| |  | \| \| |  |  |  |  |  |  |
| 3 | Vvi-Vitvi08g02000\_t001 |  | \| \| |  | \| \| |  | Atr-ERM93553 |  |  |  |  |  |  |
| 3 | Vvi-Vitvi08g00129\_t001.1.6037826f |  | \| \| |  | \| \| |  | \| \| |  |  |  |  |  |  |
| 3 | Vvi-Vitvi08g00130\_t001 |  | \| \| |  | \| \| |  | Atr-ERM93555 |  |  |  |  |  |  |
| 3 | Vvi-Vitvi08g00132\_t001 |  | \| \| |  | \| \| |  | Atr-ERM93557 |  |  |  |  |  |  |
| 3 | Vvi-Vitvi08g00133\_t001 |  | \| \| |  | \| \| |  | Atr-ERM93558 |  |  |  |  |  |  |
| 3 | Vvi-Vitvi08g00134\_t001 |  | Atr-ERM93584 |  | \| \| |  | \| \| |  |  |  |  |  |  |
| 3 | Vvi-Vitvi08g04024\_t001 |  | \| \| |  | \| \| |  | \| \| |  |  |  |  |  |  |
| 3 | Vvi-Vitvi08g00135\_t003 |  | \| \| |  | Atr-ERM93583 |  | \| \| |  |  |  |  |  |  |
| 3 | Vvi-Vitvi08g04025\_t001 |  | \| \| |  | \| \| |  | \| \| |  |  |  |  |  |  |
| 3 | Vvi-Vitvi08g04026\_t001 |  | \| \| |  | \| \| |  | \| \| |  |  |  |  |  |  |
| 3 | Vvi-Vitvi08g00137\_t001 |  | \| \| |  | Atr-ERM93580 |  | \| \| |  |  |  |  |  |  |
| 3 | Vvi-Vitvi08g00138\_t001 |  | \| \| |  | \| \| |  | \| \| |  |  |  |  |  |  |
| 3 | Vvi-Vitvi08g04027\_t001 |  | \| \| |  | \| \| |  | \| \| |  |  |  |  |  |  |
| 3 | Vvi-Vitvi08g00140\_t001 |  | \| \| |  | Atr-ERM93579 |  | \| \| |  |  |  |  |  |  |
| 3 | Vvi-Vitvi08g00142\_t001 |  | \| \| |  | \| \| |  | \| \| |  |  |  |  |  |  |
| 3 | Vvi-Vitvi08g04028\_t001 |  | \| \| |  | \| \| |  | \| \| |  |  |  |  |  |  |
| 3 | Vvi-Vitvi08g00144\_t001 |  | \| \| |  | \| \| |  | \| \| |  |  |  |  |  |  |
| 3 | Vvi-Vitvi08g00145\_t001 |  | \| \| |  | \| \| |  | \| \| |  |  |  |  |  |  |
| 3 | Vvi-Vitvi08g00146\_t001 |  | \| \| |  | \| \| |  | Atr-ERM93569 |  |  |  |  |  |  |
| 3 | Vvi-Vitvi08g00148\_t001 |  | \| \| |  | \| \| |  | \| \| |  |  |  |  |  |  |
| 3 | Vvi-Vitvi08g00151\_t001 |  | \| \| |  | \| \| |  | \| \| |  |  |  |  |  |  |
| 3 | Vvi-Vitvi08g00152\_t001 |  | \| \| |  | Atr-ERM93567 |  | \| \| |  |  |  |  |  |  |
| 3 | Vvi-Vitvi08g00153\_t001 |  | \| \| |  | \| \| |  | \| \| |  |  |  |  |  |  |
| 3 | Vvi-Vitvi08g02002\_t001 |  | \| \| |  | \| \| |  | \| \| |  |  |  |  |  |  |
| 3 | Vvi-Vitvi08g00155\_t001 |  | \| \| |  | \| \| |  | \| \| |  |  |  |  |  |  |
| 3 | Vvi-Vitvi08g04029\_t001 |  | \| \| |  | \| \| |  | \| \| |  |  |  |  |  |  |
| 3 | Vvi-Vitvi08g00157\_t001 |  | \| \| |  | \| \| |  | \| \| |  |  |  |  |  |  |
| 3 | Vvi-Vitvi08g00159\_t001 |  | \| \| |  | \| \| |  | \| \| |  |  |  |  |  |  |
| 3 | Vvi-Vitvi08g02003\_t001 |  | \| \| |  | \| \| |  | \| \| |  |  |  |  |  |  |
| 3 | Vvi-Vitvi08g00160\_t001 |  | \| \| |  | Atr-ERM93563 |  | \| \| |  |  |  |  |  |  |
| 2 | Vvi-Vitvi08g00161\_t001 |  | Atr-ERM93593 |  |  |  | \| \| |  |  |  |  |  |  |
| 2 | Vvi-Vitvi08g00163\_t001 |  | \| \| |  |  |  | Atr-ERM93591 |  |  |  |  |  |  |
| 2 | Vvi-Vitvi08g02004\_t001 |  | \| \| |  |  |  | \| \| |  |  |  |  |  |  |
| 2 | Vvi-Vitvi08g02006\_t001 |  | \| \| |  |  |  | \| \| |  |  |  |  |  |  |
| 2 | Vvi-Vitvi08g02007\_t001 |  | \| \| |  |  |  | \| \| |  |  |  |  |  |  |
| 2 | Vvi-Vitvi08g02008\_t001 |  | \| \| |  |  |  | \| \| |  |  |  |  |  |  |
| 2 | Vvi-Vitvi08g04030\_t001 |  | \| \| |  |  |  | \| \| |  |  |  |  |  |  |
| 2 | Vvi-Vitvi08g00169\_t001 |  | \| \| |  |  |  | \| \| |  |  |  |  |  |  |
| 2 | Vvi-Vitvi08g04031\_t001 |  | \| \| |  |  |  | \| \| |  |  |  |  |  |  |
| 2 | Vvi-Vitvi08g00171\_t001 |  | \| \| |  |  |  | \| \| |  |  |  |  |  |  |
| 2 | Vvi-Vitvi08g04032\_t001 |  | \| \| |  |  |  | \| \| |  |  |  |  |  |  |
| 2 | Vvi-Vitvi08g00172\_t001 |  | \| \| |  |  |  | \| \| |  |  |  |  |  |  |
| 2 | Vvi-Vitvi08g00173\_t001 |  | \| \| |  |  |  | Atr-ERM93597 |  |  |  |  |  |  |
| 2 | Vvi-Vitvi08g04033\_t001 |  | \| \| |  |  |  | \| \| |  |  |  |  |  |  |
| 2 | Vvi-Vitvi08g00174\_t002 |  | Atr-ERM93596 |  |  |  | \| \| |  |  |  |  |  |  |
| 2 | Vvi-Vitvi08g00175\_t001 |  | \| \| |  |  |  | \| \| |  |  |  |  |  |  |
| 2 | Vvi-Vitvi08g04034\_t002 |  | \| \| |  |  |  | \| \| |  |  |  |  |  |  |
| 2 | Vvi-Vitvi08g00177\_t001 |  | \| \| |  |  |  | \| \| |  |  |  |  |  |  |
| 2 | Vvi-Vitvi08g00178\_t001 |  | \| \| |  |  |  | \| \| |  |  |  |  |  |  |
| 2 | Vvi-Vitvi08g04035\_t001 |  | \| \| |  |  |  | \| \| |  |  |  |  |  |  |
| 2 | Vvi-Vitvi08g00180\_t001 |  | Atr-ERM93613 |  |  |  | \| \| |  |  |  |  |  |  |
| 2 | Vvi-Vitvi08g00182\_t001 |  | \| \| |  |  |  | \| \| |  |  |  |  |  |  |
| 2 | Vvi-Vitvi08g02016\_t001 |  | \| \| |  |  |  | \| \| |  |  |  |  |  |  |
| 2 | Vvi-Vitvi08g02017\_t001 |  | \| \| |  |  |  | \| \| |  |  |  |  |  |  |
| 2 | Vvi-Vitvi08g04036\_t001 |  | \| \| |  |  |  | \| \| |  |  |  |  |  |  |
| 2 | Vvi-Vitvi08g00184\_t001 |  | \| \| |  |  |  | \| \| |  |  |  |  |  |  |
| 2 | Vvi-Vitvi08g00185\_t001 |  | \| \| |  |  |  | \| \| |  |  |  |  |  |  |
| 2 | Vvi-Vitvi08g00186\_t001 |  | \| \| |  |  |  | Atr-ERM93610 |  |  |  |  |  |  |
| 2 | Vvi-Vitvi08g00187\_t001 |  | \| \| |  |  |  | \| \| |  |  |  |  |  |  |
| 2 | Vvi-Vitvi08g00188\_t001 |  | \| \| |  |  |  | \| \| |  |  |  |  |  |  |
| 2 | Vvi-Vitvi08g00189\_t003 |  | \| \| |  |  |  | \| \| |  |  |  |  |  |  |
| 2 | Vvi-Vitvi08g04037\_t001 |  | \| \| |  |  |  | \| \| |  |  |  |  |  |  |
| 2 | Vvi-Vitvi08g04038\_t001 |  | \| \| |  |  |  | \| \| |  |  |  |  |  |  |
| 2 | Vvi-Vitvi08g04039\_t001 |  | \| \| |  |  |  | \| \| |  |  |  |  |  |  |
| 2 | Vvi-Vitvi08g04040\_t001 |  | \| \| |  |  |  | \| \| |  |  |  |  |  |  |
| 2 | Vvi-Vitvi08g04041\_t001 |  | \| \| |  |  |  | \| \| |  |  |  |  |  |  |
| 2 | Vvi-Vitvi08g00192\_t001 |  | \| \| |  |  |  | \| \| |  |  |  |  |  |  |
| 2 | Vvi-Vitvi08g00193\_t001 |  | \| \| |  |  |  | \| \| |  |  |  |  |  |  |
| 2 | Vvi-Vitvi08g00195\_t001 |  | Atr-ERM93628 |  |  |  | \| \| |  |  |  |  |  |  |
| 1 | Vvi-Vitvi08g04042\_t001 |  |  |  |  |  | \| \| |  |  |  |  |  |  |
| 1 | Vvi-Vitvi08g04043\_t001 |  |  |  |  |  | \| \| |  |  |  |  |  |  |
| 1 | Vvi-Vitvi08g04044\_t001 |  |  |  |  |  | \| \| |  |  |  |  |  |  |
| 1 | Vvi-Vitvi08g04045\_t001 |  |  |  |  |  | \| \| |  |  |  |  |  |  |
| 1 | Vvi-Vitvi08g00197\_t001 |  |  |  |  |  | \| \| |  |  |  |  |  |  |
| 1 | Vvi-Vitvi08g00198\_t001 |  |  |  |  |  | Atr-ERM93613 |  |  |  |  |  |  |
| 1 | Vvi-Vitvi08g02021\_t001 |  |  |  |  |  | Atr-ERM93614 |  |  |  |  |  |  |
| 1 | Vvi-Vitvi08g04046\_t001 |  |  |  |  |  | \| \| |  |  |  |  |  |  |
| 1 | Vvi-Vitvi08g04047\_t001 |  |  |  |  |  | \| \| |  |  |  |  |  |  |
| 1 | Vvi-Vitvi08g00200\_t001 |  |  |  |  |  | Atr-ERM93621 |  |  |  |  |  |  |
| 1 | Vvi-Vitvi08g04048\_t001 |  |  |  |  |  | \| \| |  |  |  |  |  |  |
| 1 | Vvi-Vitvi08g04049\_t001 |  |  |  |  |  | \| \| |  |  |  |  |  |  |
| 1 | Vvi-Vitvi08g04050\_t001 |  |  |  |  |  | \| \| |  |  |  |  |  |  |
| 1 | Vvi-Vitvi08g00201\_t001 |  |  |  |  |  | \| \| |  |  |  |  |  |  |
| 1 | Vvi-Vitvi08g00202\_t001 |  |  |  |  |  | Atr-ERM93622 |  |  |  |  |  |  |
| 1 | Vvi-Vitvi08g04051\_t001 |  |  |  |  |  | \| \| |  |  |  |  |  |  |
| 1 | Vvi-Vitvi08g04052\_t001 |  |  |  |  |  | \| \| |  |  |  |  |  |  |
| 1 | Vvi-Vitvi08g04053\_t001 |  |  |  |  |  | \| \| |  |  |  |  |  |  |
| 1 | Vvi-Vitvi08g00203\_t002 |  |  |  |  |  | \| \| |  |  |  |  |  |  |
| 1 | Vvi-Vitvi08g00205\_t001 |  |  |  |  |  | Atr-ERM93623 |  |  |  |  |  |  |
| 1 | Vvi-Vitvi08g00207\_t001 |  |  |  |  |  | \| \| |  |  |  |  |  |  |
| 1 | Vvi-Vitvi08g00209\_t001 |  |  |  |  |  | Atr-ERM93627 |  |  |  |  |  |  |
| 1 | Vvi-Vitvi08g00212\_t001 |  |  |  |  |  | Atr-ERM93628 |  |  |  |  |  |  |
| 1 | Vvi-Vitvi08g00213\_t001 |  |  |  |  |  | \| \| |  |  |  |  |  |  |
| 1 | Vvi-Vitvi08g00214\_t001 |  |  |  |  |  | \| \| |  |  |  |  |  |  |
| 1 | Vvi-Vitvi08g00215\_t001 |  |  |  |  |  | Atr-ERM93631 |  |  |  |  |  |  |
| 1 | Vvi-Vitvi08g00216\_t001 |  |  |  |  |  | Atr-ERM93637 |  |  |  |  |  |  |
| 1 | Vvi-Vitvi08g00217\_t001 |  |  |  |  |  | \| \| |  |  |  |  |  |  |
| 1 | Vvi-Vitvi08g04054\_t001 |  |  |  |  |  | \| \| |  |  |  |  |  |  |
| 1 | Vvi-Vitvi08g00219\_t001 |  |  |  |  |  | \| \| |  |  |  |  |  |  |
| 1 | Vvi-Vitvi08g02028\_t001 |  |  |  |  |  | Atr-ERM93643 |  |  |  |  |  |  |
| 1 | Vvi-Vitvi08g04055\_t001 |  |  |  |  |  | \| \| |  |  |  |  |  |  |
| 1 | Vvi-Vitvi08g00221\_t001 |  |  |  |  |  | \| \| |  |  |  |  |  |  |
| 1 | Vvi-Vitvi08g04056\_t001 |  |  |  |  |  | \| \| |  |  |  |  |  |  |
| 1 | Vvi-Vitvi08g00222\_t001 |  |  |  |  |  | Atr-ERM93644 |  |  |  |  |  |  |
| 1 | Vvi-Vitvi08g00223\_t001 |  |  |  |  |  | Atr-ERM93645 |  |  |  |  |  |  |
| 1 | Vvi-Vitvi08g02030\_t001 |  |  |  |  |  | \| \| |  |  |  |  |  |  |
| 1 | Vvi-Vitvi08g04057\_t001 |  |  |  |  |  | \| \| |  |  |  |  |  |  |
| 1 | Vvi-Vitvi08g04058\_t001 |  |  |  |  |  | \| \| |  |  |  |  |  |  |
| 1 | Vvi-Vitvi08g00224\_t001 |  |  |  |  |  | \| \| |  |  |  |  |  |  |
| 1 | Vvi-Vitvi08g02032\_t001 |  |  |  |  |  | \| \| |  |  |  |  |  |  |
| 1 | Vvi-Vitvi08g04059\_t001 |  |  |  |  |  | \| \| |  |  |  |  |  |  |
| 1 | Vvi-Vitvi08g00225\_t001 |  |  |  |  |  | Atr-ERM93649 |  |  |  |  |  |  |
| 1 | Vvi-Vitvi08g04060\_t001 |  |  |  |  |  | \| \| |  |  |  |  |  |  |
| 1 | Vvi-Vitvi08g00227\_t001 |  |  |  |  |  | \| \| |  |  |  |  |  |  |
| 1 | Vvi-Vitvi08g00230\_t001 |  |  |  |  |  | \| \| |  |  |  |  |  |  |
| 1 | Vvi-Vitvi08g00233\_t001 |  |  |  |  |  | \| \| |  |  |  |  |  |  |
| 1 | Vvi-Vitvi08g00234\_t001 |  |  |  |  |  | \| \| |  |  |  |  |  |  |
| 1 | Vvi-Vitvi08g00235\_t003 |  |  |  |  |  | Atr-ERM93654 |  |  |  |  |  |  |
| 1 | Vvi-Vitvi08g00238\_t001 |  |  |  |  |  | \| \| |  |  |  |  |  |  |
| 1 | Vvi-Vitvi08g00239\_t001 |  |  |  |  |  | \| \| |  |  |  |  |  |  |
| 1 | Vvi-Vitvi08g04061\_t001 |  |  |  |  |  | \| \| |  |  |  |  |  |  |
| 1 | Vvi-Vitvi08g00240\_t001 |  |  |  |  |  | \| \| |  |  |  |  |  |  |
| 1 | Vvi-Vitvi08g02035\_t001 |  |  |  |  |  | \| \| |  |  |  |  |  |  |
| 1 | Vvi-Vitvi08g04062\_t001 |  |  |  |  |  | \| \| |  |  |  |  |  |  |
| 2 | Vvi-Vitvi08g00241\_t001 |  | Atr-ERM93681 |  |  |  | \| \| |  |  |  |  |  |  |
| 2 | Vvi-Vitvi08g00243\_t001 |  | \| \| |  |  |  | \| \| |  |  |  |  |  |  |
| 2 | Vvi-Vitvi08g04063\_t001 |  | \| \| |  |  |  | \| \| |  |  |  |  |  |  |
| 2 | Vvi-Vitvi08g02036\_t001 |  | \| \| |  |  |  | Atr-ERM93662 |  |  |  |  |  |  |
| 2 | Vvi-Vitvi08g00245\_t001 |  | \| \| |  |  |  | \| \| |  |  |  |  |  |  |
| 2 | Vvi-Vitvi08g04064\_t001 |  | \| \| |  |  |  | \| \| |  |  |  |  |  |  |
| 2 | Vvi-Vitvi08g04065\_t001 |  | \| \| |  |  |  | \| \| |  |  |  |  |  |  |
| 2 | Vvi-Vitvi08g04066\_t001 |  | \| \| |  |  |  | \| \| |  |  |  |  |  |  |
| 2 | Vvi-Vitvi08g04067\_t001 |  | \| \| |  |  |  | \| \| |  |  |  |  |  |  |
| 2 | Vvi-Vitvi08g01967\_t001 |  | \| \| |  |  |  | \| \| |  |  |  |  |  |  |
| 2 | Vvi-Vitvi08g04068\_t001 |  | \| \| |  |  |  | \| \| |  |  |  |  |  |  |
| 2 | Vvi-Vitvi08g00256\_t001 |  | \| \| |  |  |  | \| \| |  |  |  |  |  |  |
| 2 | Vvi-Vitvi08g00258\_t001 |  | Atr-ERM93680 |  |  |  | \| \| |  |  |  |  |  |  |
| 2 | Vvi-Vitvi08g00259\_t001 |  | \| \| |  |  |  | \| \| |  |  |  |  |  |  |
| 2 | Vvi-Vitvi08g04069\_t001 |  | \| \| |  |  |  | \| \| |  |  |  |  |  |  |
| 2 | Vvi-Vitvi08g02039\_t001 |  | Atr-ERM93675 |  |  |  | \| \| |  |  |  |  |  |  |
| 2 | Vvi-Vitvi08g00261\_t001 |  | \| \| |  |  |  | Atr-ERM93674 |  |  |  |  |  |  |
| 2 | Vvi-Vitvi08g00262\_t001 |  | Atr-ERM93669 |  |  |  | \| \| |  |  |  |  |  |  |
| 2 | Vvi-Vitvi08g04070\_t001 |  | \| \| |  |  |  | \| \| |  |  |  |  |  |  |
| 2 | Vvi-Vitvi08g00263\_t001 |  | \| \| |  |  |  | \| \| |  |  |  |  |  |  |
| 2 | Vvi-Vitvi08g04071\_t001 |  | \| \| |  |  |  | \| \| |  |  |  |  |  |  |
| 2 | Vvi-Vitvi08g04072\_t001 |  | \| \| |  |  |  | \| \| |  |  |  |  |  |  |
| 2 | Vvi-Vitvi08g00265\_t001 |  | \| \| |  |  |  | \| \| |  |  |  |  |  |  |
| 2 | Vvi-Vitvi08g04073\_t001 |  | \| \| |  |  |  | \| \| |  |  |  |  |  |  |
| 2 | Vvi-Vitvi08g04074\_t001 |  | \| \| |  |  |  | \| \| |  |  |  |  |  |  |
| 2 | Vvi-Vitvi08g04075\_t001 |  | \| \| |  |  |  | \| \| |  |  |  |  |  |  |
| 2 | Vvi-Vitvi08g00271\_t002 |  | Atr-ERM93667 |  |  |  | \| \| |  |  |  |  |  |  |
| 2 | Vvi-Vitvi08g00272\_t001 |  | \| \| |  |  |  | \| \| |  |  |  |  |  |  |
| 2 | Vvi-Vitvi08g00274\_t001 |  | Atr-ERM93666 |  |  |  | \| \| |  |  |  |  |  |  |
| 2 | Vvi-Vitvi08g04076\_t001 |  | \| \| |  |  |  | \| \| |  |  |  |  |  |  |
| 2 | Vvi-Vitvi08g02044\_t001 |  | \| \| |  |  |  | \| \| |  |  |  |  |  |  |
| 2 | Vvi-Vitvi08g00281\_t001 |  | \| \| |  |  |  | \| \| |  |  |  |  |  |  |
| 2 | Vvi-Vitvi08g00282\_t001 |  | \| \| |  |  |  | \| \| |  |  |  |  |  |  |
| 2 | Vvi-Vitvi08g00283\_t001 |  | \| \| |  |  |  | \| \| |  |  |  |  |  |  |
| 2 | Vvi-Vitvi08g02046\_t001 |  | \| \| |  |  |  | \| \| |  |  |  |  |  |  |
| 2 | Vvi-Vitvi08g00289\_t002 |  | \| \| |  |  |  | Atr-ERM93686 |  |  |  |  |  |  |
| 1 | Vvi-Vitvi08g00290\_t001 |  | \| \| |  |  |  |  |  |  |  |  |  |  |
| 1 | Vvi-Vitvi08g00291\_t001 |  | \| \| |  |  |  |  |  |  |  |  |  |  |
| 1 | Vvi-Vitvi08g04077\_t001 |  | \| \| |  |  |  |  |  |  |  |  |  |  |
| 1 | Vvi-Vitvi08g00292\_t001 |  | \| \| |  |  |  |  |  |  |  |  |  |  |
| 1 | Vvi-Vitvi08g00294\_t001 |  | \| \| |  |  |  |  |  |  |  |  |  |  |
| 1 | Vvi-Vitvi08g00297\_t001 |  | Atr-ERM93660 |  |  |  |  |  |  |  |  |  |  |
| 1 | Vvi-Vitvi08g00298\_t001 |  | Atr-ERM93658 |  |  |  |  |  |  |  |  |  |  |
| 0 | Vvi-Vitvi08g00300\_t001 |  |  |  |  |  |  |  |  |  |  |  |  |
| 0 | Vvi-Vitvi08g00301\_t001 |  |  |  |  |  |  |  |  |  |  |  |  |
| 0 | Vvi-Vitvi08g04078\_t001 |  |  |  |  |  |  |  |  |  |  |  |  |
| 0 | Vvi-Vitvi08g04079\_t001 |  |  |  |  |  |  |  |  |  |  |  |  |
| 0 | Vvi-Vitvi08g04080\_t001 |  |  |  |  |  |  |  |  |  |  |  |  |
| 0 | Vvi-Vitvi08g04081\_t001 |  |  |  |  |  |  |  |  |  |  |  |  |
| 0 | Vvi-Vitvi08g04082\_t001 |  |  |  |  |  |  |  |  |  |  |  |  |
| 0 | Vvi-Vitvi08g04083\_t001 |  |  |  |  |  |  |  |  |  |  |  |  |
| 0 | Vvi-Vitvi08g00621\_t001 |  |  |  |  |  |  |  |  |  |  |  |  |
| 0 | Vvi-Vitvi08g00623\_t001 |  |  |  |  |  |  |  |  |  |  |  |  |
| 0 | Vvi-Vitvi08g00624\_t001 |  |  |  |  |  |  |  |  |  |  |  |  |
| 0 | Vvi-Vitvi08g04084\_t001 |  |  |  |  |  |  |  |  |  |  |  |  |
| 0 | Vvi-Vitvi08g02052\_t001 |  |  |  |  |  |  |  |  |  |  |  |  |
| 1 | Vvi-Vitvi08g02051\_t001 |  | Atr-ERN16085 |  |  |  |  |  |  |  |  |  |  |
| 1 | Vvi-Vitvi08g04085\_t001 |  | \| \| |  |  |  |  |  |  |  |  |  |  |
| 1 | Vvi-Vitvi08g00630\_t001 |  | \| \| |  |  |  |  |  |  |  |  |  |  |
| 1 | Vvi-Vitvi08g00632\_t001 |  | Atr-ERN16088 |  |  |  |  |  |  |  |  |  |  |
| 1 | Vvi-Vitvi08g00633\_t001 |  | Atr-ERN16094 |  |  |  |  |  |  |  |  |  |  |
| 1 | Vvi-Vitvi08g04086\_t001 |  | \| \| |  |  |  |  |  |  |  |  |  |  |
| 1 | Vvi-Vitvi08g00636\_t001 |  | Atr-ERN16096 |  |  |  |  |  |  |  |  |  |  |
| 1 | Vvi-Vitvi08g00637\_t001 |  | Atr-ERN16097 |  |  |  |  |  |  |  |  |  |  |
| 1 | Vvi-Vitvi08g00639\_t001 |  | Atr-ERN16100 |  |  |  |  |  |  |  |  |  |  |
| 1 | Vvi-Vitvi08g00640\_t001 |  | Atr-ERN16104 |  |  |  |  |  |  |  |  |  |  |
| 0 | Vvi-Vitvi08g00641\_t001 |  |  |  |  |  |  |  |  |  |  |  |  |
| 1 | Vvi-Vitvi08g00642\_t001 |  | Atr-ERN05223 |  |  |  |  |  |  |  |  |  |  |
| 1 | Vvi-Vitvi08g00643\_t001 |  | Atr-ERN05230 |  |  |  |  |  |  |  |  |  |  |
| 1 | Vvi-Vitvi08g00644\_t001 |  | Atr-ERN05241 |  |  |  |  |  |  |  |  |  |  |
| 1 | Vvi-Vitvi08g00645\_t001 |  | \| \| |  |  |  |  |  |  |  |  |  |  |
| 1 | Vvi-Vitvi08g04087\_t001 |  | \| \| |  |  |  |  |  |  |  |  |  |  |
| 1 | Vvi-Vitvi08g00650\_t002 |  | \| \| |  |  |  |  |  |  |  |  |  |  |
| 1 | Vvi-Vitvi08g00653\_t001 |  | \| \| |  |  |  |  |  |  |  |  |  |  |
| 1 | Vvi-Vitvi08g00654\_t001 |  | \| \| |  |  |  |  |  |  |  |  |  |  |
| 1 | Vvi-Vitvi08g04088\_t001 |  | \| \| |  |  |  |  |  |  |  |  |  |  |
| 1 | Vvi-Vitvi08g00656\_t001 |  | Atr-ERN05253 |  |  |  |  |  |  |  |  |  |  |
| 1 | Vvi-Vitvi08g02055\_t001 |  | \| \| |  |  |  |  |  |  |  |  |  |  |
| 1 | Vvi-Vitvi08g00658\_t001 |  | \| \| |  |  |  |  |  |  |  |  |  |  |
| 1 | Vvi-Vitvi08g00661\_t001 |  | \| \| |  |  |  |  |  |  |  |  |  |  |
| 1 | Vvi-Vitvi08g04089\_t001 |  | \| \| |  |  |  |  |  |  |  |  |  |  |
| 1 | Vvi-Vitvi08g02056\_t001 |  | \| \| |  |  |  |  |  |  |  |  |  |  |
| 1 | Vvi-Vitvi08g00662\_t001 |  | Atr-ERN05262 |  |  |  |  |  |  |  |  |  |  |
| 1 | Vvi-Vitvi08g00665\_t001 |  | Atr-ERN05264 |  |  |  |  |  |  |  |  |  |  |
| 1 | Vvi-Vitvi08g00666\_t001 |  | Atr-ERN05265 |  |  |  |  |  |  |  |  |  |  |
| 1 | Vvi-Vitvi08g04090\_t001 |  | \| \| |  |  |  |  |  |  |  |  |  |  |
| 1 | Vvi-Vitvi08g00667\_t001 |  | Atr-ERN05269 |  |  |  |  |  |  |  |  |  |  |
| 1 | Vvi-Vitvi08g00668\_t001 |  | \| \| |  |  |  |  |  |  |  |  |  |  |
| 1 | Vvi-Vitvi08g04091\_t001 |  | \| \| |  |  |  |  |  |  |  |  |  |  |
| 1 | Vvi-Vitvi08g00671\_t001 |  | Atr-ERN05281 |  |  |  |  |  |  |  |  |  |  |
| 1 | Vvi-Vitvi08g00672\_t001 |  | Atr-ERN05282 |  |  |  |  |  |  |  |  |  |  |
| 1 | Vvi-Vitvi08g00673\_t001 |  | \| \| |  |  |  |  |  |  |  |  |  |  |
| 1 | Vvi-Vitvi08g00674\_t002 |  | Atr-ERN05299 |  |  |  |  |  |  |  |  |  |  |
| 1 | Vvi-Vitvi08g02058\_t001 |  | \| \| |  |  |  |  |  |  |  |  |  |  |
| 1 | Vvi-Vitvi08g00675\_t001 |  | \| \| |  |  |  |  |  |  |  |  |  |  |
| 1 | Vvi-Vitvi08g00676\_t002 |  | Atr-ERN05301 |  |  |  |  |  |  |  |  |  |  |
| 1 | Vvi-Vitvi08g00678\_t001 |  | Atr-ERN05306 |  |  |  |  |  |  |  |  |  |  |
| 1 | Vvi-Vitvi08g00679\_t001 |  | Atr-ERN05313 |  |  |  |  |  |  |  |  |  |  |
| 1 | Vvi-Vitvi08g04092\_t001 |  | \| \| |  |  |  |  |  |  |  |  |  |  |
| 1 | Vvi-Vitvi08g00682\_t001 |  | \| \| |  |  |  |  |  |  |  |  |  |  |
| 1 | Vvi-Vitvi08g00684\_t001 |  | \| \| |  |  |  |  |  |  |  |  |  |  |
| 1 | Vvi-Vitvi08g00685\_t001 |  | Atr-ERN05319 |  |  |  |  |  |  |  |  |  |  |
| 1 | Vvi-Vitvi08g00686\_t001 |  | \| \| |  |  |  |  |  |  |  |  |  |  |
| 1 | Vvi-Vitvi08g00687\_t001 |  | Atr-ERN05325 |  |  |  |  |  |  |  |  |  |  |
| 1 | Vvi-Vitvi08g00689\_t001 |  | \| \| |  |  |  |  |  |  |  |  |  |  |
| 1 | Vvi-Vitvi08g00690\_t001 |  | Atr-ERN05331 |  |  |  |  |  |  |  |  |  |  |
| 0 | Vvi-Vitvi08g00691\_t001 |  |  |  |  |  |  |  |  |  |  |  |  |
| 0 | Vvi-Vitvi08g04093\_t001 |  |  |  |  |  |  |  |  |  |  |  |  |
| 0 | Vvi-Vitvi08g02060\_t001 |  |  |  |  |  |  |  |  |  |  |  |  |
| 0 | Vvi-Vitvi08g00700\_t001 |  |  |  |  |  |  |  |  |  |  |  |  |
| 0 | Vvi-Vitvi08g04094\_t001 |  |  |  |  |  |  |  |  |  |  |  |  |
| 0 | Vvi-Vitvi08g04095\_t001 |  |  |  |  |  |  |  |  |  |  |  |  |
| 0 | Vvi-Vitvi08g04096\_t001 |  |  |  |  |  |  |  |  |  |  |  |  |
| 0 | Vvi-Vitvi08g00703\_t001 |  |  |  |  |  |  |  |  |  |  |  |  |
| 0 | Vvi-Vitvi08g04097\_t001 |  |  |  |  |  |  |  |  |  |  |  |  |
| 0 | Vvi-Vitvi08g04098\_t001 |  |  |  |  |  |  |  |  |  |  |  |  |
| 0 | Vvi-Vitvi08g04099\_t001 |  |  |  |  |  |  |  |  |  |  |  |  |
| 0 | Vvi-Vitvi08g04100\_t001 |  |  |  |  |  |  |  |  |  |  |  |  |
| 0 | Vvi-Vitvi08g04101\_t001 |  |  |  |  |  |  |  |  |  |  |  |  |
| 0 | Vvi-Vitvi08g02065\_t001 |  |  |  |  |  |  |  |  |  |  |  |  |
| 0 | Vvi-Vitvi08g04102\_t001 |  |  |  |  |  |  |  |  |  |  |  |  |
| 0 | Vvi-Vitvi08g04103\_t001 |  |  |  |  |  |  |  |  |  |  |  |  |
| 0 | Vvi-Vitvi08g04104\_t001 |  |  |  |  |  |  |  |  |  |  |  |  |
| 0 | Vvi-Vitvi08g04105\_t001 |  |  |  |  |  |  |  |  |  |  |  |  |
| 0 | Vvi-Vitvi08g04106\_t001 |  |  |  |  |  |  |  |  |  |  |  |  |
| 0 | Vvi-Vitvi08g02066\_t001 |  |  |  |  |  |  |  |  |  |  |  |  |
| 0 | Vvi-Vitvi08g02067\_t001 |  |  |  |  |  |  |  |  |  |  |  |  |
| 0 | Vvi-Vitvi08g04107\_t001 |  |  |  |  |  |  |  |  |  |  |  |  |
| 0 | Vvi-Vitvi08g02068\_t001 |  |  |  |  |  |  |  |  |  |  |  |  |
| 0 | Vvi-Vitvi08g04108\_t001 |  |  |  |  |  |  |  |  |  |  |  |  |
| 0 | Vvi-Vitvi08g04109\_t001 |  |  |  |  |  |  |  |  |  |  |  |  |
| 0 | Vvi-Vitvi08g02069\_t001 |  |  |  |  |  |  |  |  |  |  |  |  |
| 0 | Vvi-Vitvi08g04110\_t001 |  |  |  |  |  |  |  |  |  |  |  |  |
| 0 | Vvi-Vitvi08g04111\_t001 |  |  |  |  |  |  |  |  |  |  |  |  |
| 0 | Vvi-Vitvi08g04112\_t001 |  |  |  |  |  |  |  |  |  |  |  |  |
| 0 | Vvi-Vitvi08g00715\_t001 |  |  |  |  |  |  |  |  |  |  |  |  |
| 0 | Vvi-Vitvi08g00716\_t001 |  |  |  |  |  |  |  |  |  |  |  |  |
| 0 | Vvi-Vitvi08g00718\_t001 |  |  |  |  |  |  |  |  |  |  |  |  |
| 0 | Vvi-Vitvi08g00719\_t001 |  |  |  |  |  |  |  |  |  |  |  |  |
| 0 | Vvi-Vitvi08g00720\_t001 |  |  |  |  |  |  |  |  |  |  |  |  |
| 0 | Vvi-Vitvi08g00721\_t001 |  |  |  |  |  |  |  |  |  |  |  |  |
| 0 | Vvi-Vitvi08g00723\_t001 |  |  |  |  |  |  |  |  |  |  |  |  |
| 0 | Vvi-Vitvi08g00725\_t001 |  |  |  |  |  |  |  |  |  |  |  |  |
| 0 | Vvi-Vitvi08g00726\_t001 |  |  |  |  |  |  |  |  |  |  |  |  |
| 0 | Vvi-Vitvi08g04113\_t001 |  |  |  |  |  |  |  |  |  |  |  |  |
| 0 | Vvi-Vitvi08g04114\_t001 |  |  |  |  |  |  |  |  |  |  |  |  |
| 0 | Vvi-Vitvi08g00728\_t001 |  |  |  |  |  |  |  |  |  |  |  |  |
| 0 | Vvi-Vitvi08g04115\_t001 |  |  |  |  |  |  |  |  |  |  |  |  |
| 0 | Vvi-Vitvi08g00729\_t001 |  |  |  |  |  |  |  |  |  |  |  |  |
| 0 | Vvi-Vitvi08g00730\_t001 |  |  |  |  |  |  |  |  |  |  |  |  |
| 0 | Vvi-Vitvi08g00731\_t001 |  |  |  |  |  |  |  |  |  |  |  |  |
| 0 | Vvi-Vitvi08g04116\_t001 |  |  |  |  |  |  |  |  |  |  |  |  |
| 0 | Vvi-Vitvi08g00735\_t001 |  |  |  |  |  |  |  |  |  |  |  |  |
| 0 | Vvi-Vitvi08g04117\_t001 |  |  |  |  |  |  |  |  |  |  |  |  |
| 0 | Vvi-Vitvi08g00736\_t001 |  |  |  |  |  |  |  |  |  |  |  |  |
| 0 | Vvi-Vitvi08g00738\_t001 |  |  |  |  |  |  |  |  |  |  |  |  |
| 0 | Vvi-Vitvi08g02071\_t001 |  |  |  |  |  |  |  |  |  |  |  |  |
| 0 | Vvi-Vitvi08g00739\_t001 |  |  |  |  |  |  |  |  |  |  |  |  |
| 0 | Vvi-Vitvi08g00740\_t001 |  |  |  |  |  |  |  |  |  |  |  |  |
| 0 | Vvi-Vitvi08g04118\_t001 |  |  |  |  |  |  |  |  |  |  |  |  |
| 0 | Vvi-Vitvi08g04119\_t001 |  |  |  |  |  |  |  |  |  |  |  |  |
| 0 | Vvi-Vitvi08g00743\_t001 |  |  |  |  |  |  |  |  |  |  |  |  |
| 0 | Vvi-Vitvi08g00744\_t001 |  |  |  |  |  |  |  |  |  |  |  |  |
| 0 | Vvi-Vitvi08g00746\_t001 |  |  |  |  |  |  |  |  |  |  |  |  |
| 0 | Vvi-Vitvi08g00747\_t001 |  |  |  |  |  |  |  |  |  |  |  |  |
| 0 | Vvi-Vitvi08g04120\_t001 |  |  |  |  |  |  |  |  |  |  |  |  |
| 0 | Vvi-Vitvi08g00749\_t001 |  |  |  |  |  |  |  |  |  |  |  |  |
| 0 | Vvi-Vitvi08g04121\_t001 |  |  |  |  |  |  |  |  |  |  |  |  |
| 0 | Vvi-Vitvi08g01969\_t001 |  |  |  |  |  |  |  |  |  |  |  |  |
| 0 | Vvi-Vitvi08g00752\_t001 |  |  |  |  |  |  |  |  |  |  |  |  |
| 0 | Vvi-Vitvi08g02074\_t001 |  |  |  |  |  |  |  |  |  |  |  |  |
| 0 | Vvi-Vitvi08g00754\_t001 |  |  |  |  |  |  |  |  |  |  |  |  |
| 0 | Vvi-Vitvi08g00755\_t002 |  |  |  |  |  |  |  |  |  |  |  |  |
| 0 | Vvi-Vitvi08g00756\_t001 |  |  |  |  |  |  |  |  |  |  |  |  |
| 0 | Vvi-Vitvi08g00757\_t001 |  |  |  |  |  |  |  |  |  |  |  |  |
| 0 | Vvi-Vitvi08g02076\_t001 |  |  |  |  |  |  |  |  |  |  |  |  |
| 0 | Vvi-Vitvi08g00761\_t001 |  |  |  |  |  |  |  |  |  |  |  |  |
| 0 | Vvi-Vitvi08g04122\_t001 |  |  |  |  |  |  |  |  |  |  |  |  |
| 0 | Vvi-Vitvi08g04123\_t001 |  |  |  |  |  |  |  |  |  |  |  |  |
| 0 | Vvi-Vitvi08g00766\_t001 |  |  |  |  |  |  |  |  |  |  |  |  |
| 0 | Vvi-Vitvi08g04124\_t001 |  |  |  |  |  |  |  |  |  |  |  |  |
| 0 | Vvi-Vitvi08g00767\_t001 |  |  |  |  |  |  |  |  |  |  |  |  |
| 0 | Vvi-Vitvi08g00768\_t001 |  |  |  |  |  |  |  |  |  |  |  |  |
| 1 | Vvi-Vitvi08g00769\_t001 |  | Atr-ERM95539 |  |  |  |  |  |  |  |  |  |  |
| 1 | Vvi-Vitvi08g00771\_t001 |  | \| \| |  |  |  |  |  |  |  |  |  |  |
| 1 | Vvi-Vitvi08g00772\_t001 |  | \| \| |  |  |  |  |  |  |  |  |  |  |
| 1 | Vvi-Vitvi08g00773\_t001 |  | \| \| |  |  |  |  |  |  |  |  |  |  |
| 1 | Vvi-Vitvi08g00777\_t001 |  | \| \| |  |  |  |  |  |  |  |  |  |  |
| 1 | Vvi-Vitvi08g00778\_t001 |  | Atr-ERM95542 |  |  |  |  |  |  |  |  |  |  |
| 1 | Vvi-Vitvi08g04125\_t001 |  | \| \| |  |  |  |  |  |  |  |  |  |  |
| 1 | Vvi-Vitvi08g00780\_t001 |  | Atr-ERM95546 |  |  |  |  |  |  |  |  |  |  |
| 1 | Vvi-Vitvi08g00781\_t001 |  | Atr-ERM95551 |  |  |  |  |  |  |  |  |  |  |
| 1 | Vvi-Vitvi08g00782\_t001 |  | \| \| |  |  |  |  |  |  |  |  |  |  |
| 1 | Vvi-Vitvi08g00783\_t003 |  | Atr-ERM95561 |  |  |  |  |  |  |  |  |  |  |
| 1 | Vvi-Vitvi08g00784\_t001 |  | \| \| |  |  |  |  |  |  |  |  |  |  |
| 1 | Vvi-Vitvi08g00785\_t001 |  | \| \| |  |  |  |  |  |  |  |  |  |  |
| 1 | Vvi-Vitvi08g04126\_t001 |  | \| \| |  |  |  |  |  |  |  |  |  |  |
| 1 | Vvi-Vitvi08g00786\_t002 |  | Atr-ERM95568 |  |  |  |  |  |  |  |  |  |  |
| 1 | Vvi-Vitvi08g00788\_t002 |  | Atr-ERM95569 |  |  |  |  |  |  |  |  |  |  |
| 1 | Vvi-Vitvi08g00789\_t001 |  | \| \| |  |  |  |  |  |  |  |  |  |  |
| 1 | Vvi-Vitvi08g00790\_t001 |  | \| \| |  |  |  |  |  |  |  |  |  |  |
| 1 | Vvi-Vitvi08g02080\_t001 |  | Atr-ERM95575 |  |  |  |  |  |  |  |  |  |  |
| 1 | Vvi-Vitvi08g00791\_t001 |  | \| \| |  |  |  |  |  |  |  |  |  |  |
| 1 | Vvi-Vitvi08g00792\_t001 |  | \| \| |  |  |  |  |  |  |  |  |  |  |
| 1 | Vvi-Vitvi08g00793\_t001 |  | Atr-ERM95579 |  |  |  |  |  |  |  |  |  |  |
| 1 | Vvi-Vitvi08g00794\_t001 |  | Atr-ERM95580 |  |  |  |  |  |  |  |  |  |  |
| 1 | Vvi-Vitvi08g00795\_t002 |  | Atr-ERM95581 |  |  |  |  |  |  |  |  |  |  |
| 1 | Vvi-Vitvi08g02084\_t001 |  | \| \| |  |  |  |  |  |  |  |  |  |  |
| 1 | Vvi-Vitvi08g04127\_t001 |  | \| \| |  |  |  |  |  |  |  |  |  |  |
| 1 | Vvi-Vitvi08g00796\_t001 |  | \| \| |  |  |  |  |  |  |  |  |  |  |
| 1 | Vvi-Vitvi08g00797\_t002 |  | Atr-ERM95583 |  |  |  |  |  |  |  |  |  |  |
| 1 | Vvi-Vitvi08g04128\_t001 |  | \| \| |  |  |  |  |  |  |  |  |  |  |
| 1 | Vvi-Vitvi08g00798\_t001 |  | \| \| |  |  |  |  |  |  |  |  |  |  |
| 1 | Vvi-Vitvi08g00799\_t001 |  | Atr-ERM95585 |  |  |  |  |  |  |  |  |  |  |
| 1 | Vvi-Vitvi08g00801\_t001 |  | Atr-ERM95587 |  |  |  |  |  |  |  |  |  |  |
| 1 | Vvi-Vitvi08g04129\_t001 |  | \| \| |  |  |  |  |  |  |  |  |  |  |
| 1 | Vvi-Vitvi08g00802\_t001 |  | Atr-ERM95606 |  |  |  |  |  |  |  |  |  |  |
| 1 | Vvi-Vitvi08g00803\_t001 |  | Atr-ERN05359 |  |  |  |  |  |  |  |  |  |  |
| 1 | Vvi-Vitvi08g00804\_t001 |  | \| \| |  |  |  |  |  |  |  |  |  |  |
| 1 | Vvi-Vitvi08g00805\_t001 |  | Atr-ERN05360 |  |  |  |  |  |  |  |  |  |  |
| 1 | Vvi-Vitvi08g00806\_t001 |  | Atr-ERN05362 |  |  |  |  |  |  |  |  |  |  |
| 1 | Vvi-Vitvi08g04130\_t001 |  | \| \| |  |  |  |  |  |  |  |  |  |  |
| 1 | Vvi-Vitvi08g00808\_t001 |  | \| \| |  |  |  |  |  |  |  |  |  |  |
| 1 | Vvi-Vitvi08g00809\_t001 |  | \| \| |  |  |  |  |  |  |  |  |  |  |
| 1 | Vvi-Vitvi08g04131\_t001 |  | \| \| |  |  |  |  |  |  |  |  |  |  |
| 1 | Vvi-Vitvi08g02086\_t001 |  | Atr-ERN05369 |  |  |  |  |  |  |  |  |  |  |
| 1 | Vvi-Vitvi08g00812\_t001 |  | Atr-ERN05370 |  |  |  |  |  |  |  |  |  |  |
| 1 | Vvi-Vitvi08g02087\_t001 |  | Atr-ERN05372 |  |  |  |  |  |  |  |  |  |  |
| 1 | Vvi-Vitvi08g00813\_t001 |  | \| \| |  |  |  |  |  |  |  |  |  |  |
| 1 | Vvi-Vitvi08g04132\_t001 |  | \| \| |  |  |  |  |  |  |  |  |  |  |
| 1 | Vvi-Vitvi08g00816\_t001 |  | Atr-ERN05374 |  |  |  |  |  |  |  |  |  |  |
| 1 | Vvi-Vitvi08g00817\_t001 |  | Atr-ERN05375 |  |  |  |  |  |  |  |  |  |  |
| 1 | Vvi-Vitvi08g00819\_t001 |  | Atr-ERN05377 |  |  |  |  |  |  |  |  |  |  |
| 2 | Vvi-Vitvi08g00820\_t001 |  | \| \| |  | Atr-ERN04107 |  |  |  |  |  |  |  |  |
| 3 | Vvi-Vitvi08g00821\_t001 |  | \| \| |  | \| \| |  | Atr-ERN05419 |  |  |  |  |  |  |
| 3 | Vvi-Vitvi08g00822\_t001 |  | \| \| |  | \| \| |  | \| \| |  |  |  |  |  |  |
| 3 | Vvi-Vitvi08g00823\_t001 |  | \| \| |  | \| \| |  | \| \| |  |  |  |  |  |  |
| 3 | Vvi-Vitvi08g00824\_t001 |  | \| \| |  | \| \| |  | \| \| |  |  |  |  |  |  |
| 3 | Vvi-Vitvi08g02089\_t001 |  | \| \| |  | \| \| |  | Atr-ERN05413 |  |  |  |  |  |  |
| 3 | Vvi-Vitvi08g00825\_t002 |  | \| \| |  | \| \| |  | Atr-ERN05412 |  |  |  |  |  |  |
| 3 | Vvi-Vitvi08g00826\_t002 |  | \| \| |  | \| \| |  | Atr-ERN05411 |  |  |  |  |  |  |
| 3 | Vvi-Vitvi08g00827\_t001 |  | \| \| |  | \| \| |  | \| \| |  |  |  |  |  |  |
| 3 | Vvi-Vitvi08g00828\_t001 |  | Atr-ERN05398 |  | \| \| |  | \| \| |  |  |  |  |  |  |
| 3 | Vvi-Vitvi08g00829\_t001 |  | Atr-ERN05399 |  | \| \| |  | \| \| |  |  |  |  |  |  |
| 3 | Vvi-Vitvi08g04133\_t001 |  | \| \| |  | \| \| |  | \| \| |  |  |  |  |  |  |
| 3 | Vvi-Vitvi08g00833\_t001 |  | \| \| |  | \| \| |  | \| \| |  |  |  |  |  |  |
| 3 | Vvi-Vitvi08g00834\_t001 |  | \| \| |  | \| \| |  | Atr-ERN05407 |  |  |  |  |  |  |
| 3 | Vvi-Vitvi08g04134\_t001 |  | \| \| |  | \| \| |  | \| \| |  |  |  |  |  |  |
| 3 | Vvi-Vitvi08g00835\_t001 |  | Atr-ERN05404 |  | \| \| |  | Atr-ERN05398 |  |  |  |  |  |  |
| 2 | Vvi-Vitvi08g00836\_t001 |  |  |  | \| \| |  | Atr-ERN05392 |  |  |  |  |  |  |
| 2 | Vvi-Vitvi08g00838\_t001 |  |  |  | \| \| |  | \| \| |  |  |  |  |  |  |
| 2 | Vvi-Vitvi08g00839\_t001 |  |  |  | \| \| |  | Atr-ERN05388 |  |  |  |  |  |  |
| 2 | Vvi-Vitvi08g00840\_t001 |  |  |  | \| \| |  | Atr-ERN05383 |  |  |  |  |  |  |
| 2 | Vvi-Vitvi08g00841\_t001 |  |  |  | \| \| |  | Atr-ERN05382 |  |  |  |  |  |  |
| 2 | Vvi-Vitvi08g00843\_t001 |  |  |  | \| \| |  | Atr-ERN05381 |  |  |  |  |  |  |
| 2 | Vvi-Vitvi08g00844\_t001 |  |  |  | \| \| |  | Atr-ERN05379 |  |  |  |  |  |  |
| 2 | Vvi-Vitvi08g00845\_t001 |  |  |  | \| \| |  | Atr-ERN05378 |  |  |  |  |  |  |
| 1 | Vvi-Vitvi08g00846\_t001 |  |  |  | \| \| |  |  |  |  |  |  |  |  |
| 1 | Vvi-Vitvi08g00847\_t001 |  |  |  | Atr-ERN04120 |  |  |  |  |  |  |  |  |
| 1 | Vvi-Vitvi08g00849\_t001 |  |  |  | \| \| |  |  |  |  |  |  |  |  |
| 1 | Vvi-Vitvi08g00850\_t001 |  |  |  | Atr-ERN04122 |  |  |  |  |  |  |  |  |
| 1 | Vvi-Vitvi08g00851\_t001 |  |  |  | \| \| |  |  |  |  |  |  |  |  |
| 1 | Vvi-Vitvi08g00852\_t001 |  |  |  | Atr-ERN04133 |  |  |  |  |  |  |  |  |
| 1 | Vvi-Vitvi08g00853\_t001 |  |  |  | Atr-ERN04138 |  |  |  |  |  |  |  |  |
| 1 | Vvi-Vitvi08g02093\_t001 |  |  |  | \| \| |  |  |  |  |  |  |  |  |
| 1 | Vvi-Vitvi08g00854\_t001 |  |  |  | \| \| |  |  |  |  |  |  |  |  |
| 1 | Vvi-Vitvi08g02094\_t001 |  |  |  | \| \| |  |  |  |  |  |  |  |  |
| 1 | Vvi-Vitvi08g04135\_t001 |  |  |  | \| \| |  |  |  |  |  |  |  |  |
| 1 | Vvi-Vitvi08g00857\_t001 |  |  |  | Atr-ERN04146 |  |  |  |  |  |  |  |  |
| 1 | Vvi-Vitvi08g02095\_t001 |  |  |  | \| \| |  |  |  |  |  |  |  |  |
| 1 | Vvi-Vitvi08g00858\_t001 |  |  |  | Atr-ERN04150 |  |  |  |  |  |  |  |  |
| 1 | Vvi-Vitvi08g00859\_t001 |  |  |  | Atr-ERN04159 |  |  |  |  |  |  |  |  |
| 1 | Vvi-Vitvi08g00860\_t001 |  |  |  | Atr-ERN04163 |  |  |  |  |  |  |  |  |
| 1 | Vvi-Vitvi08g00861\_t001 |  |  |  | Atr-ERN04164 |  |  |  |  |  |  |  |  |
| 1 | Vvi-Vitvi08g04136\_t001 |  |  |  | \| \| |  |  |  |  |  |  |  |  |
| 1 | Vvi-Vitvi08g00862\_t001 |  |  |  | Atr-ERN04166 |  |  |  |  |  |  |  |  |
| 1 | Vvi-Vitvi08g00863\_t001 |  |  |  | Atr-ERN04168 |  |  |  |  |  |  |  |  |
| 1 | Vvi-Vitvi08g00864\_t001 |  |  |  | Atr-ERN04170 |  |  |  |  |  |  |  |  |
| 1 | Vvi-Vitvi08g00865\_t001 |  |  |  | Atr-ERN04171 |  |  |  |  |  |  |  |  |
| 1 | Vvi-Vitvi08g00866\_t001 |  |  |  | Atr-ERN04190 |  |  |  |  |  |  |  |  |
| 1 | Vvi-Vitvi08g00867\_t001 |  |  |  | \| \| |  |  |  |  |  |  |  |  |
| 1 | Vvi-Vitvi08g00868\_t001 |  |  |  | \| \| |  |  |  |  |  |  |  |  |
| 1 | Vvi-Vitvi08g00869\_t001 |  |  |  | Atr-ERN04193 |  |  |  |  |  |  |  |  |
| 1 | Vvi-Vitvi08g04137\_t001 |  |  |  | \| \| |  |  |  |  |  |  |  |  |
| 1 | Vvi-Vitvi08g00870\_t001 |  |  |  | Atr-ERN04195 |  |  |  |  |  |  |  |  |
| 1 | Vvi-Vitvi08g00871\_t001 |  |  |  | Atr-ERN04196 |  |  |  |  |  |  |  |  |
| 1 | Vvi-Vitvi08g00872\_t001 |  |  |  | Atr-ERN04220 |  |  |  |  |  |  |  |  |
| 1 | Vvi-Vitvi08g04138\_t001 |  |  |  | \| \| |  |  |  |  |  |  |  |  |
| 1 | Vvi-Vitvi08g04139\_t001 |  |  |  | \| \| |  |  |  |  |  |  |  |  |
| 1 | Vvi-Vitvi08g00874\_t001 |  |  |  | Atr-ERN04221 |  |  |  |  |  |  |  |  |
| 1 | Vvi-Vitvi08g02097\_t001 |  |  |  | Atr-ERN04222 |  |  |  |  |  |  |  |  |
| 1 | Vvi-Vitvi08g00877\_t001 |  |  |  | Atr-ERN04226 |  |  |  |  |  |  |  |  |
| 1 | Vvi-Vitvi08g04140\_t001 |  |  |  | Atr-ERN04229 |  |  |  |  |  |  |  |  |
| 1 | Vvi-Vitvi08g04141\_t001 |  |  |  | Atr-ERN04230 |  |  |  |  |  |  |  |  |
| 1 | Vvi-Vitvi08g04142\_t002 |  |  |  | Atr-ERN04233 |  |  |  |  |  |  |  |  |
| 1 | Vvi-Vitvi08g00881\_t001 |  |  |  | Atr-ERN04235 |  |  |  |  |  |  |  |  |
| 1 | Vvi-Vitvi08g00883\_t004 |  |  |  | Atr-ERN04238 |  |  |  |  |  |  |  |  |
| 0 | Vvi-Vitvi08g00884\_t001 |  |  |  |  |  |  |  |  |  |  |  |  |
| 0 | Vvi-Vitvi08g04143\_t001 |  |  |  |  |  |  |  |  |  |  |  |  |
| 0 | Vvi-Vitvi08g02099\_t001 |  |  |  |  |  |  |  |  |  |  |  |  |
| 0 | Vvi-Vitvi08g02100\_t001 |  |  |  |  |  |  |  |  |  |  |  |  |
| 0 | Vvi-Vitvi08g00885\_t001 |  |  |  |  |  |  |  |  |  |  |  |  |
| 0 | Vvi-Vitvi08g00886\_t001 |  |  |  |  |  |  |  |  |  |  |  |  |
| 0 | Vvi-Vitvi08g00887\_t001 |  |  |  |  |  |  |  |  |  |  |  |  |
| 0 | Vvi-Vitvi08g04144\_t001 |  |  |  |  |  |  |  |  |  |  |  |  |
| 0 | Vvi-Vitvi08g00888\_t001 |  |  |  |  |  |  |  |  |  |  |  |  |
| 0 | Vvi-Vitvi08g00890\_t001 |  |  |  |  |  |  |  |  |  |  |  |  |
| 0 | Vvi-Vitvi08g00891\_t001 |  |  |  |  |  |  |  |  |  |  |  |  |
| 0 | Vvi-Vitvi08g04145\_t001 |  |  |  |  |  |  |  |  |  |  |  |  |
| 0 | Vvi-Vitvi08g02101\_t001 |  |  |  |  |  |  |  |  |  |  |  |  |
| 0 | Vvi-Vitvi08g00892\_t002 |  |  |  |  |  |  |  |  |  |  |  |  |
| 0 | Vvi-Vitvi08g00893\_t001 |  |  |  |  |  |  |  |  |  |  |  |  |
| 0 | Vvi-Vitvi08g04146\_t001 |  |  |  |  |  |  |  |  |  |  |  |  |
| 0 | Vvi-Vitvi08g00894\_t001 |  |  |  |  |  |  |  |  |  |  |  |  |
| 0 | Vvi-Vitvi08g00895\_t002 |  |  |  |  |  |  |  |  |  |  |  |  |
| 0 | Vvi-Vitvi08g02102\_t001 |  |  |  |  |  |  |  |  |  |  |  |  |
| 0 | Vvi-Vitvi08g02103\_t001 |  |  |  |  |  |  |  |  |  |  |  |  |
| 0 | Vvi-Vitvi08g04147\_t001 |  |  |  |  |  |  |  |  |  |  |  |  |
| 0 | Vvi-Vitvi08g00896\_t001 |  |  |  |  |  |  |  |  |  |  |  |  |
| 0 | Vvi-Vitvi08g00897\_t001 |  |  |  |  |  |  |  |  |  |  |  |  |
| 0 | Vvi-Vitvi08g04148\_t001 |  |  |  |  |  |  |  |  |  |  |  |  |
| 0 | Vvi-Vitvi08g04149\_t001 |  |  |  |  |  |  |  |  |  |  |  |  |
| 0 | Vvi-Vitvi08g04150\_t001 |  |  |  |  |  |  |  |  |  |  |  |  |
| 0 | Vvi-Vitvi08g04151\_t001 |  |  |  |  |  |  |  |  |  |  |  |  |
| 1 | Vvi-Vitvi08g00900\_t001 |  | Atr-ERM98215 |  |  |  |  |  |  |  |  |  |  |
| 1 | Vvi-Vitvi08g00901\_t001 |  | Atr-ERM98189 |  |  |  |  |  |  |  |  |  |  |
| 1 | Vvi-Vitvi08g00902\_t001 |  | \| \| |  |  |  |  |  |  |  |  |  |  |
| 1 | Vvi-Vitvi08g00903\_t001 |  | Atr-ERM98188 |  |  |  |  |  |  |  |  |  |  |
| 1 | Vvi-Vitvi08g00904\_t001 |  | Atr-ERM98186 |  |  |  |  |  |  |  |  |  |  |
| 1 | Vvi-Vitvi08g00905\_t001 |  | Atr-ERM98185 |  |  |  |  |  |  |  |  |  |  |
| 1 | Vvi-Vitvi08g02105\_t001 |  | \| \| |  |  |  |  |  |  |  |  |  |  |
| 1 | Vvi-Vitvi08g00906\_t001 |  | Atr-ERM98183 |  |  |  |  |  |  |  |  |  |  |
| 1 | Vvi-Vitvi08g02106\_t001 |  | Atr-ERM98182 |  |  |  |  |  |  |  |  |  |  |
| 1 | Vvi-Vitvi08g02107\_t001 |  | Atr-ERM98172 |  |  |  |  |  |  |  |  |  |  |
| 1 | Vvi-Vitvi08g00908\_t003 |  | Atr-ERM98171 |  |  |  |  |  |  |  |  |  |  |
| 1 | Vvi-Vitvi08g04152\_t001 |  | Atr-ERM98170 |  |  |  |  |  |  |  |  |  |  |
| 1 | Vvi-Vitvi08g00910\_t001 |  | Atr-ERM98169 |  |  |  |  |  |  |  |  |  |  |
| 1 | Vvi-Vitvi08g00911\_t001 |  | Atr-ERM98168 |  |  |  |  |  |  |  |  |  |  |
| 1 | Vvi-Vitvi08g00912\_t001 |  | Atr-ERM98167 |  |  |  |  |  |  |  |  |  |  |
| 1 | Vvi-Vitvi08g00913\_t001 |  | Atr-ERM98166 |  |  |  |  |  |  |  |  |  |  |
| 1 | Vvi-Vitvi08g04153\_t001 |  | \| \| |  |  |  |  |  |  |  |  |  |  |
| 1 | Vvi-Vitvi08g00914\_t001 |  | Atr-ERM98142 |  |  |  |  |  |  |  |  |  |  |
| 0 | Vvi-Vitvi08g00915\_t001 |  |  |  |  |  |  |  |  |  |  |  |  |
| 0 | Vvi-Vitvi08g00916\_t001 |  |  |  |  |  |  |  |  |  |  |  |  |
| 0 | Vvi-Vitvi08g00917\_t001 |  |  |  |  |  |  |  |  |  |  |  |  |
| 0 | Vvi-Vitvi08g00921\_t001 |  |  |  |  |  |  |  |  |  |  |  |  |
| 0 | Vvi-Vitvi08g00922\_t001 |  |  |  |  |  |  |  |  |  |  |  |  |
| 0 | Vvi-Vitvi08g04154\_t001 |  |  |  |  |  |  |  |  |  |  |  |  |
| 0 | Vvi-Vitvi08g00923\_t001 |  |  |  |  |  |  |  |  |  |  |  |  |
| 0 | Vvi-Vitvi08g00924\_t001 |  |  |  |  |  |  |  |  |  |  |  |  |
| 0 | Vvi-Vitvi08g00925\_t001 |  |  |  |  |  |  |  |  |  |  |  |  |
| 0 | Vvi-Vitvi08g00926\_t001 |  |  |  |  |  |  |  |  |  |  |  |  |
| 0 | Vvi-Vitvi08g02109\_t001 |  |  |  |  |  |  |  |  |  |  |  |  |
| 0 | Vvi-Vitvi08g00928\_t001 |  |  |  |  |  |  |  |  |  |  |  |  |
| 0 | Vvi-Vitvi08g02111\_t001 |  |  |  |  |  |  |  |  |  |  |  |  |
| 0 | Vvi-Vitvi08g00929\_t001 |  |  |  |  |  |  |  |  |  |  |  |  |
| 0 | Vvi-Vitvi08g00930\_t003 |  |  |  |  |  |  |  |  |  |  |  |  |
| 0 | Vvi-Vitvi08g04155\_t001 |  |  |  |  |  |  |  |  |  |  |  |  |
| 0 | Vvi-Vitvi08g00931\_t001 |  |  |  |  |  |  |  |  |  |  |  |  |
| 0 | Vvi-Vitvi08g02112\_t001 |  |  |  |  |  |  |  |  |  |  |  |  |
| 0 | Vvi-Vitvi08g00932\_t001 |  |  |  |  |  |  |  |  |  |  |  |  |
| 0 | Vvi-Vitvi08g00933\_t001 |  |  |  |  |  |  |  |  |  |  |  |  |
| 0 | Vvi-Vitvi08g00934\_t001 |  |  |  |  |  |  |  |  |  |  |  |  |
| 1 | Vvi-Vitvi08g00935\_t001 |  | Atr-ERM99072 |  |  |  |  |  |  |  |  |  |  |
| 1 | Vvi-Vitvi08g00936\_t001 |  | \| \| |  |  |  |  |  |  |  |  |  |  |
| 1 | Vvi-Vitvi08g04156\_t001 |  | \| \| |  |  |  |  |  |  |  |  |  |  |
| 1 | Vvi-Vitvi08g04157\_t001 |  | \| \| |  |  |  |  |  |  |  |  |  |  |
| 1 | Vvi-Vitvi08g02113\_t001 |  | \| \| |  |  |  |  |  |  |  |  |  |  |
| 1 | Vvi-Vitvi08g00937\_t001 |  | \| \| |  |  |  |  |  |  |  |  |  |  |
| 1 | Vvi-Vitvi08g00939\_t001 |  | Atr-ERM99075 |  |  |  |  |  |  |  |  |  |  |
| 1 | Vvi-Vitvi08g00940\_t001 |  | Atr-ERM99077 |  |  |  |  |  |  |  |  |  |  |
| 1 | Vvi-Vitvi08g00941\_t001 |  | \| \| |  |  |  |  |  |  |  |  |  |  |
| 1 | Vvi-Vitvi08g00942\_t001 |  | \| \| |  |  |  |  |  |  |  |  |  |  |
| 1 | Vvi-Vitvi08g04158\_t001 |  | \| \| |  |  |  |  |  |  |  |  |  |  |
| 1 | Vvi-Vitvi08g00943\_t001 |  | Atr-ERM99086 |  |  |  |  |  |  |  |  |  |  |
| 1 | Vvi-Vitvi08g00944\_t001 |  | \| \| |  |  |  |  |  |  |  |  |  |  |
| 1 | Vvi-Vitvi08g00945\_t001 |  | \| \| |  |  |  |  |  |  |  |  |  |  |
| 1 | Vvi-Vitvi08g00946\_t001 |  | \| \| |  |  |  |  |  |  |  |  |  |  |
| 1 | Vvi-Vitvi08g00947\_t001 |  | Atr-ERM99093 |  |  |  |  |  |  |  |  |  |  |
| 1 | Vvi-Vitvi08g04159\_t001 |  | \| \| |  |  |  |  |  |  |  |  |  |  |
| 1 | Vvi-Vitvi08g00948\_t001 |  | \| \| |  |  |  |  |  |  |  |  |  |  |
| 1 | Vvi-Vitvi08g02114\_t001 |  | \| \| |  |  |  |  |  |  |  |  |  |  |
| 1 | Vvi-Vitvi08g04160\_t001 |  | \| \| |  |  |  |  |  |  |  |  |  |  |
| 1 | Vvi-Vitvi08g00949\_t001 |  | \| \| |  |  |  |  |  |  |  |  |  |  |
| 1 | Vvi-Vitvi08g04161\_t001 |  | \| \| |  |  |  |  |  |  |  |  |  |  |
| 1 | Vvi-Vitvi08g00950\_t001 |  | Atr-ERM99094 |  |  |  |  |  |  |  |  |  |  |
| 1 | Vvi-Vitvi08g04162\_t001 |  | \| \| |  |  |  |  |  |  |  |  |  |  |
| 1 | Vvi-Vitvi08g00953\_t001 |  | \| \| |  |  |  |  |  |  |  |  |  |  |
| 1 | Vvi-Vitvi08g00954\_t003 |  | Atr-ERM99095 |  |  |  |  |  |  |  |  |  |  |
| 1 | Vvi-Vitvi08g00955\_t001 |  | Atr-ERM99098 |  |  |  |  |  |  |  |  |  |  |
| 1 | Vvi-Vitvi08g00956\_t001 |  | \| \| |  |  |  |  |  |  |  |  |  |  |
| 1 | Vvi-Vitvi08g00957\_t001 |  | \| \| |  |  |  |  |  |  |  |  |  |  |
| 1 | Vvi-Vitvi08g00958\_t001 |  | Atr-ERM99103 |  |  |  |  |  |  |  |  |  |  |
| 1 | Vvi-Vitvi08g04163\_t001 |  | \| \| |  |  |  |  |  |  |  |  |  |  |
| 1 | Vvi-Vitvi08g00959\_t001 |  | \| \| |  |  |  |  |  |  |  |  |  |  |
| 1 | Vvi-Vitvi08g00960\_t001 |  | \| \| |  |  |  |  |  |  |  |  |  |  |
| 1 | Vvi-Vitvi08g00961\_t001 |  | \| \| |  |  |  |  |  |  |  |  |  |  |
| 1 | Vvi-Vitvi08g00962\_t001 |  | Atr-ERM99112 |  |  |  |  |  |  |  |  |  |  |
| 1 | Vvi-Vitvi08g00963\_t001 |  | \| \| |  |  |  |  |  |  |  |  |  |  |
| 1 | Vvi-Vitvi08g00965\_t001 |  | \| \| |  |  |  |  |  |  |  |  |  |  |
| 1 | Vvi-Vitvi08g00967\_t001 |  | Atr-ERM99113 |  |  |  |  |  |  |  |  |  |  |
| 1 | Vvi-Vitvi08g04164\_t001 |  | \| \| |  |  |  |  |  |  |  |  |  |  |
| 1 | Vvi-Vitvi08g02126\_t001 |  | \| \| |  |  |  |  |  |  |  |  |  |  |
| 1 | Vvi-Vitvi08g04165\_t003 |  | \| \| |  |  |  |  |  |  |  |  |  |  |
| 1 | Vvi-Vitvi08g02128\_t001 |  | \| \| |  |  |  |  |  |  |  |  |  |  |
| 1 | Vvi-Vitvi08g02129\_t001 |  | \| \| |  |  |  |  |  |  |  |  |  |  |
| 1 | Vvi-Vitvi08g04166\_t001 |  | \| \| |  |  |  |  |  |  |  |  |  |  |
| 1 | Vvi-Vitvi08g00970\_t001 |  | \| \| |  |  |  |  |  |  |  |  |  |  |
| 1 | Vvi-Vitvi08g00971\_t001 |  | Atr-ERM99118 |  |  |  |  |  |  |  |  |  |  |
| 1 | Vvi-Vitvi08g04167\_t001 |  | \| \| |  |  |  |  |  |  |  |  |  |  |
| 1 | Vvi-Vitvi08g00972\_t001 |  | Atr-ERM99119 |  |  |  |  |  |  |  |  |  |  |
| 1 | Vvi-Vitvi08g04168\_t001 |  | \| \| |  |  |  |  |  |  |  |  |  |  |
| 1 | Vvi-Vitvi08g02131\_t002 |  | \| \| |  |  |  |  |  |  |  |  |  |  |
| 1 | Vvi-Vitvi08g00974\_t001 |  | \| \| |  |  |  |  |  |  |  |  |  |  |
| 1 | Vvi-Vitvi08g00976\_t001 |  | \| \| |  |  |  |  |  |  |  |  |  |  |
| 1 | Vvi-Vitvi08g00977\_t001 |  | Atr-ERM99121 |  |  |  |  |  |  |  |  |  |  |
| 1 | Vvi-Vitvi08g00978\_t001 |  | \| \| |  |  |  |  |  |  |  |  |  |  |
| 1 | Vvi-Vitvi08g04169\_t001 |  | \| \| |  |  |  |  |  |  |  |  |  |  |
| 1 | Vvi-Vitvi08g00979\_t001 |  | Atr-ERM99122 |  |  |  |  |  |  |  |  |  |  |
| 1 | Vvi-Vitvi08g00981\_t001 |  | Atr-ERM99128 |  |  |  |  |  |  |  |  |  |  |
| 1 | Vvi-Vitvi08g00982\_t001 |  | Atr-ERM99130 |  |  |  |  |  |  |  |  |  |  |
| 1 | Vvi-Vitvi08g00983\_t001 |  | Atr-ERM99134 |  |  |  |  |  |  |  |  |  |  |
| 1 | Vvi-Vitvi08g00984\_t001 |  | Atr-ERM99135 |  |  |  |  |  |  |  |  |  |  |
| 1 | Vvi-Vitvi08g00986\_t001 |  | Atr-ERM99136 |  |  |  |  |  |  |  |  |  |  |
| 0 | Vvi-Vitvi08g00987\_t001 |  |  |  |  |  |  |  |  |  |  |  |  |
| 0 | Vvi-Vitvi08g00988\_t001 |  |  |  |  |  |  |  |  |  |  |  |  |
| 0 | Vvi-Vitvi08g04170\_t001 |  |  |  |  |  |  |  |  |  |  |  |  |
| 0 | Vvi-Vitvi08g04171\_t001 |  |  |  |  |  |  |  |  |  |  |  |  |
| 0 | Vvi-Vitvi08g00989\_t001 |  |  |  |  |  |  |  |  |  |  |  |  |
| 0 | Vvi-Vitvi08g00992\_t001 |  |  |  |  |  |  |  |  |  |  |  |  |
| 0 | Vvi-Vitvi08g00993\_t001 |  |  |  |  |  |  |  |  |  |  |  |  |
| 0 | Vvi-Vitvi08g02133\_t001 |  |  |  |  |  |  |  |  |  |  |  |  |
| 0 | Vvi-Vitvi08g00994\_t001 |  |  |  |  |  |  |  |  |  |  |  |  |
| 0 | Vvi-Vitvi08g00995\_t001 |  |  |  |  |  |  |  |  |  |  |  |  |
| 0 | Vvi-Vitvi08g00996\_t001 |  |  |  |  |  |  |  |  |  |  |  |  |
| 0 | Vvi-Vitvi08g00998\_t001 |  |  |  |  |  |  |  |  |  |  |  |  |
| 0 | Vvi-Vitvi08g02134\_t001 |  |  |  |  |  |  |  |  |  |  |  |  |
| 0 | Vvi-Vitvi08g02135\_t001 |  |  |  |  |  |  |  |  |  |  |  |  |
| 0 | Vvi-Vitvi08g04172\_t001 |  |  |  |  |  |  |  |  |  |  |  |  |
| 0 | Vvi-Vitvi08g00999\_t001 |  |  |  |  |  |  |  |  |  |  |  |  |
| 0 | Vvi-Vitvi08g04173\_t001 |  |  |  |  |  |  |  |  |  |  |  |  |
| 0 | Vvi-Vitvi08g02136\_t001 |  |  |  |  |  |  |  |  |  |  |  |  |
| 0 | Vvi-Vitvi08g01001\_t001 |  |  |  |  |  |  |  |  |  |  |  |  |
| 0 | Vvi-Vitvi08g01002\_t001 |  |  |  |  |  |  |  |  |  |  |  |  |
| 0 | Vvi-Vitvi08g02137\_t001 |  |  |  |  |  |  |  |  |  |  |  |  |
| 0 | Vvi-Vitvi08g01004\_t001 |  |  |  |  |  |  |  |  |  |  |  |  |
| 0 | Vvi-Vitvi08g04174\_t001 |  |  |  |  |  |  |  |  |  |  |  |  |
| 0 | Vvi-Vitvi08g01006\_t001 |  |  |  |  |  |  |  |  |  |  |  |  |
| 0 | Vvi-Vitvi08g01007\_t001 |  |  |  |  |  |  |  |  |  |  |  |  |
| 1 | Vvi-Vitvi08g02139\_t001 |  | Atr-ERM94837 |  |  |  |  |  |  |  |  |  |  |
| 1 | Vvi-Vitvi08g01008\_t001 |  | \| \| |  |  |  |  |  |  |  |  |  |  |
| 1 | Vvi-Vitvi08g01009\_t001 |  | \| \| |  |  |  |  |  |  |  |  |  |  |
| 1 | Vvi-Vitvi08g01010\_t005 |  | Atr-ERM94842 |  |  |  |  |  |  |  |  |  |  |
| 1 | Vvi-Vitvi08g04175\_t001 |  | \| \| |  |  |  |  |  |  |  |  |  |  |
| 1 | Vvi-Vitvi08g01011\_t001 |  | \| \| |  |  |  |  |  |  |  |  |  |  |
| 1 | Vvi-Vitvi08g01012\_t002 |  | \| \| |  |  |  |  |  |  |  |  |  |  |
| 1 | Vvi-Vitvi08g01013\_t001 |  | Atr-ERM94849 |  |  |  |  |  |  |  |  |  |  |
| 1 | Vvi-Vitvi08g01014\_t001 |  | Atr-ERM94850 |  |  |  |  |  |  |  |  |  |  |
| 1 | Vvi-Vitvi08g02140\_t003 |  | Atr-ERM94861 |  |  |  |  |  |  |  |  |  |  |
| 1 | Vvi-Vitvi08g01015\_t001 |  | Atr-ERM94866 |  |  |  |  |  |  |  |  |  |  |
| 1 | Vvi-Vitvi08g01016\_t001 |  | Atr-ERN08408 |  |  |  |  |  |  |  |  |  |  |
| 1 | Vvi-Vitvi08g01018\_t001 |  | Atr-ERN08407 |  |  |  |  |  |  |  |  |  |  |
| 1 | Vvi-Vitvi08g01019\_t001 |  | \| \| |  |  |  |  |  |  |  |  |  |  |
| 1 | Vvi-Vitvi08g04176\_t001 |  | \| \| |  |  |  |  |  |  |  |  |  |  |
| 1 | Vvi-Vitvi08g01021\_t001 |  | \| \| |  |  |  |  |  |  |  |  |  |  |
| 1 | Vvi-Vitvi08g04177\_t001 |  | \| \| |  |  |  |  |  |  |  |  |  |  |
| 2 | Vvi-Vitvi08g01023\_t001 |  | \| \| |  | Atr-ERN08378 |  |  |  |  |  |  |  |  |
| 2 | Vvi-Vitvi08g01024\_t001 |  | \| \| |  | Atr-ERN08380 |  |  |  |  |  |  |  |  |
| 2 | Vvi-Vitvi08g01026\_t001 |  | \| \| |  | Atr-ERN08387 |  |  |  |  |  |  |  |  |
| 2 | Vvi-Vitvi08g01027\_t001 |  | \| \| |  | Atr-ERN08390 |  |  |  |  |  |  |  |  |
| 2 | Vvi-Vitvi08g01028\_t001 |  | \| \| |  | Atr-ERN08391 |  |  |  |  |  |  |  |  |
| 2 | Vvi-Vitvi08g01029\_t001 |  | \| \| |  | \| \| |  |  |  |  |  |  |  |  |
| 2 | Vvi-Vitvi08g01030\_t001 |  | Atr-ERN08393 |  | Atr-ERN08393 |  |  |  |  |  |  |  |  |
| 2 | Vvi-Vitvi08g01031\_t001 |  | \| \| |  | \| \| |  |  |  |  |  |  |  |  |
| 2 | Vvi-Vitvi08g01032\_t001 |  | \| \| |  | Atr-ERN08400 |  |  |  |  |  |  |  |  |
| 1 | Vvi-Vitvi08g01033\_t001 |  | Atr-ERN08367 |  |  |  |  |  |  |  |  |  |  |
| 1 | Vvi-Vitvi08g02143\_t001 |  | \| \| |  |  |  |  |  |  |  |  |  |  |
| 1 | Vvi-Vitvi08g01035\_t001 |  | \| \| |  |  |  |  |  |  |  |  |  |  |
| 1 | Vvi-Vitvi08g02145\_t001 |  | Atr-ERN08359 |  |  |  |  |  |  |  |  |  |  |
| 1 | Vvi-Vitvi08g01036\_t001 |  | Atr-ERN08355 |  |  |  |  |  |  |  |  |  |  |
| 1 | Vvi-Vitvi08g04178\_t001 |  | \| \| |  |  |  |  |  |  |  |  |  |  |
| 1 | Vvi-Vitvi08g01037\_t001 |  | Atr-ERN08354 |  |  |  |  |  |  |  |  |  |  |
| 1 | Vvi-Vitvi08g01038\_t001 |  | Atr-ERN08352 |  |  |  |  |  |  |  |  |  |  |
| 0 | Vvi-Vitvi08g01039\_t002 |  |  |  |  |  |  |  |  |  |  |  |  |
| 0 | Vvi-Vitvi08g04179\_t001 |  |  |  |  |  |  |  |  |  |  |  |  |
| 0 | Vvi-Vitvi08g01040\_t001 |  |  |  |  |  |  |  |  |  |  |  |  |
| 0 | Vvi-Vitvi08g01041\_t001 |  |  |  |  |  |  |  |  |  |  |  |  |
| 0 | Vvi-Vitvi08g01042\_t001 |  |  |  |  |  |  |  |  |  |  |  |  |
| 0 | Vvi-Vitvi08g01043\_t001 |  |  |  |  |  |  |  |  |  |  |  |  |
| 0 | Vvi-Vitvi08g01044\_t001 |  |  |  |  |  |  |  |  |  |  |  |  |
| 0 | Vvi-Vitvi08g02147\_t001 |  |  |  |  |  |  |  |  |  |  |  |  |
| 0 | Vvi-Vitvi08g02148\_t001 |  |  |  |  |  |  |  |  |  |  |  |  |
| 0 | Vvi-Vitvi08g02149\_t001 |  |  |  |  |  |  |  |  |  |  |  |  |
| 0 | Vvi-Vitvi08g02150\_t001 |  |  |  |  |  |  |  |  |  |  |  |  |
| 0 | Vvi-Vitvi08g02151\_t001 |  |  |  |  |  |  |  |  |  |  |  |  |
| 0 | Vvi-Vitvi08g04180\_t001 |  |  |  |  |  |  |  |  |  |  |  |  |
| 0 | Vvi-Vitvi08g02152\_t001 |  |  |  |  |  |  |  |  |  |  |  |  |
| 0 | Vvi-Vitvi08g02153\_t001 |  |  |  |  |  |  |  |  |  |  |  |  |
| 0 | Vvi-Vitvi08g02154\_t001 |  |  |  |  |  |  |  |  |  |  |  |  |
| 0 | Vvi-Vitvi08g02155\_t001 |  |  |  |  |  |  |  |  |  |  |  |  |
| 0 | Vvi-Vitvi08g04181\_t001 |  |  |  |  |  |  |  |  |  |  |  |  |
| 0 | Vvi-Vitvi08g02156\_t001 |  |  |  |  |  |  |  |  |  |  |  |  |
| 0 | Vvi-Vitvi08g02157\_t001 |  |  |  |  |  |  |  |  |  |  |  |  |
| 0 | Vvi-Vitvi08g04182\_t001 |  |  |  |  |  |  |  |  |  |  |  |  |
| 0 | Vvi-Vitvi08g02158\_t001 |  |  |  |  |  |  |  |  |  |  |  |  |
| 0 | Vvi-Vitvi08g02159\_t001 |  |  |  |  |  |  |  |  |  |  |  |  |
| 0 | Vvi-Vitvi08g02160\_t001 |  |  |  |  |  |  |  |  |  |  |  |  |
| 0 | Vvi-Vitvi08g04183\_t001 |  |  |  |  |  |  |  |  |  |  |  |  |
| 0 | Vvi-Vitvi08g01047\_t001 |  |  |  |  |  |  |  |  |  |  |  |  |
| 0 | Vvi-Vitvi08g01049\_t001 |  |  |  |  |  |  |  |  |  |  |  |  |
| 0 | Vvi-Vitvi08g01050\_t003 |  |  |  |  |  |  |  |  |  |  |  |  |
| 0 | Vvi-Vitvi08g01052\_t001 |  |  |  |  |  |  |  |  |  |  |  |  |
| 0 | Vvi-Vitvi08g01053\_t001 |  |  |  |  |  |  |  |  |  |  |  |  |
| 0 | Vvi-Vitvi08g04184\_t001 |  |  |  |  |  |  |  |  |  |  |  |  |
| 0 | Vvi-Vitvi08g01054\_t001 |  |  |  |  |  |  |  |  |  |  |  |  |
| 0 | Vvi-Vitvi08g01055\_t001 |  |  |  |  |  |  |  |  |  |  |  |  |
| 0 | Vvi-Vitvi08g01056\_t001 |  |  |  |  |  |  |  |  |  |  |  |  |
| 0 | Vvi-Vitvi08g01057\_t002 |  |  |  |  |  |  |  |  |  |  |  |  |
| 0 | Vvi-Vitvi08g01058\_t001 |  |  |  |  |  |  |  |  |  |  |  |  |
| 0 | Vvi-Vitvi08g04185\_t001 |  |  |  |  |  |  |  |  |  |  |  |  |
| 0 | Vvi-Vitvi08g01059\_t001 |  |  |  |  |  |  |  |  |  |  |  |  |
| 0 | Vvi-Vitvi08g02161\_t003 |  |  |  |  |  |  |  |  |  |  |  |  |
| 0 | Vvi-Vitvi08g01060\_t002 |  |  |  |  |  |  |  |  |  |  |  |  |
| 0 | Vvi-Vitvi08g02162\_t001 |  |  |  |  |  |  |  |  |  |  |  |  |
| 0 | Vvi-Vitvi08g01061\_t001 |  |  |  |  |  |  |  |  |  |  |  |  |
| 0 | Vvi-Vitvi08g01062\_t001 |  |  |  |  |  |  |  |  |  |  |  |  |
| 0 | Vvi-Vitvi08g01063\_t001 |  |  |  |  |  |  |  |  |  |  |  |  |
| 0 | Vvi-Vitvi08g01065\_t001 |  |  |  |  |  |  |  |  |  |  |  |  |
| 0 | Vvi-Vitvi08g01067\_t001 |  |  |  |  |  |  |  |  |  |  |  |  |
| 0 | Vvi-Vitvi08g02163\_t001 |  |  |  |  |  |  |  |  |  |  |  |  |
| 0 | Vvi-Vitvi08g01068\_t001 |  |  |  |  |  |  |  |  |  |  |  |  |
| 0 | Vvi-Vitvi08g01069\_t001 |  |  |  |  |  |  |  |  |  |  |  |  |
| 0 | Vvi-Vitvi08g01070\_t001 |  |  |  |  |  |  |  |  |  |  |  |  |
| 0 | Vvi-Vitvi08g01071\_t001 |  |  |  |  |  |  |  |  |  |  |  |  |
| 0 | Vvi-Vitvi08g01072\_t001 |  |  |  |  |  |  |  |  |  |  |  |  |
| 0 | Vvi-Vitvi08g01073\_t001 |  |  |  |  |  |  |  |  |  |  |  |  |
| 0 | Vvi-Vitvi08g04186\_t001 |  |  |  |  |  |  |  |  |  |  |  |  |
| 0 | Vvi-Vitvi08g01075\_t003 |  |  |  |  |  |  |  |  |  |  |  |  |
| 0 | Vvi-Vitvi08g02164\_t001 |  |  |  |  |  |  |  |  |  |  |  |  |
| 0 | Vvi-Vitvi08g01076\_t001 |  |  |  |  |  |  |  |  |  |  |  |  |
| 0 | Vvi-Vitvi08g01077\_t001 |  |  |  |  |  |  |  |  |  |  |  |  |
| 0 | Vvi-Vitvi08g01078\_t001 |  |  |  |  |  |  |  |  |  |  |  |  |
| 0 | Vvi-Vitvi08g01079\_t001 |  |  |  |  |  |  |  |  |  |  |  |  |
| 0 | Vvi-Vitvi08g04187\_t001 |  |  |  |  |  |  |  |  |  |  |  |  |
| 0 | Vvi-Vitvi08g01081\_t001 |  |  |  |  |  |  |  |  |  |  |  |  |
| 0 | Vvi-Vitvi08g01082\_t004 |  |  |  |  |  |  |  |  |  |  |  |  |
| 0 | Vvi-Vitvi08g01083\_t001 |  |  |  |  |  |  |  |  |  |  |  |  |
| 0 | Vvi-Vitvi08g04188\_t001 |  |  |  |  |  |  |  |  |  |  |  |  |
| 0 | Vvi-Vitvi08g04189\_t001 |  |  |  |  |  |  |  |  |  |  |  |  |
| 0 | Vvi-Vitvi08g01085\_t001 |  |  |  |  |  |  |  |  |  |  |  |  |
| 0 | Vvi-Vitvi08g01087\_t001 |  |  |  |  |  |  |  |  |  |  |  |  |
| 0 | Vvi-Vitvi08g04190\_t001 |  |  |  |  |  |  |  |  |  |  |  |  |
| 0 | Vvi-Vitvi08g02166\_t001 |  |  |  |  |  |  |  |  |  |  |  |  |
| 0 | Vvi-Vitvi08g02167\_t001 |  |  |  |  |  |  |  |  |  |  |  |  |
| 0 | Vvi-Vitvi08g02168\_t002 |  |  |  |  |  |  |  |  |  |  |  |  |
| 0 | Vvi-Vitvi08g01089\_t001 |  |  |  |  |  |  |  |  |  |  |  |  |
| 0 | Vvi-Vitvi08g04191\_t001 |  |  |  |  |  |  |  |  |  |  |  |  |
| 0 | Vvi-Vitvi08g01090\_t001 |  |  |  |  |  |  |  |  |  |  |  |  |
| 0 | Vvi-Vitvi08g01091\_t002 |  |  |  |  |  |  |  |  |  |  |  |  |
| 0 | Vvi-Vitvi08g02169\_t001 |  |  |  |  |  |  |  |  |  |  |  |  |
| 0 | Vvi-Vitvi08g02170\_t001 |  |  |  |  |  |  |  |  |  |  |  |  |
| 0 | Vvi-Vitvi08g01095\_t001 |  |  |  |  |  |  |  |  |  |  |  |  |
| 0 | Vvi-Vitvi08g01096\_t001 |  |  |  |  |  |  |  |  |  |  |  |  |
| 0 | Vvi-Vitvi08g02171\_t001 |  |  |  |  |  |  |  |  |  |  |  |  |
| 0 | Vvi-Vitvi08g02172\_t001 |  |  |  |  |  |  |  |  |  |  |  |  |
| 0 | Vvi-Vitvi08g04192\_t001 |  |  |  |  |  |  |  |  |  |  |  |  |
| 0 | Vvi-Vitvi08g01097\_t001 |  |  |  |  |  |  |  |  |  |  |  |  |
| 0 | Vvi-Vitvi08g01098\_t001 |  |  |  |  |  |  |  |  |  |  |  |  |
| 0 | Vvi-Vitvi08g01099\_t001 |  |  |  |  |  |  |  |  |  |  |  |  |
| 0 | Vvi-Vitvi08g04193\_t001 |  |  |  |  |  |  |  |  |  |  |  |  |
| 0 | Vvi-Vitvi08g04194\_t001 |  |  |  |  |  |  |  |  |  |  |  |  |
| 0 | Vvi-Vitvi08g04195\_t001 |  |  |  |  |  |  |  |  |  |  |  |  |
| 0 | Vvi-Vitvi08g01101\_t001 |  |  |  |  |  |  |  |  |  |  |  |  |
| 0 | Vvi-Vitvi08g01102\_t001 |  |  |  |  |  |  |  |  |  |  |  |  |
| 0 | Vvi-Vitvi08g01103\_t001 |  |  |  |  |  |  |  |  |  |  |  |  |
| 0 | Vvi-Vitvi08g01104\_t003 |  |  |  |  |  |  |  |  |  |  |  |  |
| 0 | Vvi-Vitvi08g01105\_t001 |  |  |  |  |  |  |  |  |  |  |  |  |
| 0 | Vvi-Vitvi08g01107\_t001 |  |  |  |  |  |  |  |  |  |  |  |  |
| 0 | Vvi-Vitvi08g04196\_t001 |  |  |  |  |  |  |  |  |  |  |  |  |
| 0 | Vvi-Vitvi08g01108\_t001 |  |  |  |  |  |  |  |  |  |  |  |  |
| 0 | Vvi-Vitvi08g04197\_t001 |  |  |  |  |  |  |  |  |  |  |  |  |
| 0 | Vvi-Vitvi08g01109\_t001 |  |  |  |  |  |  |  |  |  |  |  |  |
| 0 | Vvi-Vitvi08g04198\_t001 |  |  |  |  |  |  |  |  |  |  |  |  |
| 0 | Vvi-Vitvi08g01111\_t001 |  |  |  |  |  |  |  |  |  |  |  |  |
| 0 | Vvi-Vitvi08g01112\_t001 |  |  |  |  |  |  |  |  |  |  |  |  |
| 0 | Vvi-Vitvi08g01113\_t001 |  |  |  |  |  |  |  |  |  |  |  |  |
| 0 | Vvi-Vitvi08g01114\_t001 |  |  |  |  |  |  |  |  |  |  |  |  |
| 0 | Vvi-Vitvi08g04199\_t001 |  |  |  |  |  |  |  |  |  |  |  |  |
| 1 | Vvi-Vitvi08g04200\_t001 |  | Atr-ERM95635 |  |  |  |  |  |  |  |  |  |  |
| 1 | Vvi-Vitvi08g01116\_t001 |  | \| \| |  |  |  |  |  |  |  |  |  |  |
| 1 | Vvi-Vitvi08g01117\_t001 |  | Atr-ERM95636 |  |  |  |  |  |  |  |  |  |  |
| 1 | Vvi-Vitvi08g04201\_t001 |  | \| \| |  |  |  |  |  |  |  |  |  |  |
| 1 | Vvi-Vitvi08g01118\_t001 |  | Atr-ERM95639 |  |  |  |  |  |  |  |  |  |  |
| 1 | Vvi-Vitvi08g01119\_t001 |  | \| \| |  |  |  |  |  |  |  |  |  |  |
| 1 | Vvi-Vitvi08g01120\_t001 |  | Atr-ERM95649 |  |  |  |  |  |  |  |  |  |  |
| 1 | Vvi-Vitvi08g01121\_t001 |  | \| \| |  |  |  |  |  |  |  |  |  |  |
| 1 | Vvi-Vitvi08g01122\_t001 |  | \| \| |  |  |  |  |  |  |  |  |  |  |
| 1 | Vvi-Vitvi08g01123\_t001 |  | \| \| |  |  |  |  |  |  |  |  |  |  |
| 1 | Vvi-Vitvi08g02176\_t001 |  | \| \| |  |  |  |  |  |  |  |  |  |  |
| 1 | Vvi-Vitvi08g01124\_t001 |  | Atr-ERM95663 |  |  |  |  |  |  |  |  |  |  |
| 1 | Vvi-Vitvi08g04202\_t001 |  | \| \| |  |  |  |  |  |  |  |  |  |  |
| 1 | Vvi-Vitvi08g01125\_t001 |  | Atr-ERM95664 |  |  |  |  |  |  |  |  |  |  |
| 1 | Vvi-Vitvi08g02177\_t001 |  | \| \| |  |  |  |  |  |  |  |  |  |  |
| 1 | Vvi-Vitvi08g04203\_t001 |  | \| \| |  |  |  |  |  |  |  |  |  |  |
| 1 | Vvi-Vitvi08g01127\_t002 |  | \| \| |  |  |  |  |  |  |  |  |  |  |
| 1 | Vvi-Vitvi08g01129\_t001 |  | \| \| |  |  |  |  |  |  |  |  |  |  |
| 1 | Vvi-Vitvi08g01130\_t001 |  | Atr-ERM95683 |  |  |  |  |  |  |  |  |  |  |
| 1 | Vvi-Vitvi08g01133\_t002 |  | \| \| |  |  |  |  |  |  |  |  |  |  |
| 1 | Vvi-Vitvi08g01134\_t001 |  | \| \| |  |  |  |  |  |  |  |  |  |  |
| 1 | Vvi-Vitvi08g04204\_t001 |  | \| \| |  |  |  |  |  |  |  |  |  |  |
| 1 | Vvi-Vitvi08g04205\_t001 |  | \| \| |  |  |  |  |  |  |  |  |  |  |
| 1 | Vvi-Vitvi08g04206\_t001 |  | \| \| |  |  |  |  |  |  |  |  |  |  |
| 1 | Vvi-Vitvi08g01137\_t001 |  | \| \| |  |  |  |  |  |  |  |  |  |  |
| 1 | Vvi-Vitvi08g04207\_t001 |  | \| \| |  |  |  |  |  |  |  |  |  |  |
| 1 | Vvi-Vitvi08g01139\_t001 |  | Atr-ERM95693 |  |  |  |  |  |  |  |  |  |  |
| 1 | Vvi-Vitvi08g04208\_t001 |  | \| \| |  |  |  |  |  |  |  |  |  |  |
| 1 | Vvi-Vitvi08g01140\_t002 |  | \| \| |  |  |  |  |  |  |  |  |  |  |
| 1 | Vvi-Vitvi08g01142\_t003 |  | \| \| |  |  |  |  |  |  |  |  |  |  |
| 1 | Vvi-Vitvi08g04209\_t001 |  | \| \| |  |  |  |  |  |  |  |  |  |  |
| 1 | Vvi-Vitvi08g01143\_t002 |  | \| \| |  |  |  |  |  |  |  |  |  |  |
| 1 | Vvi-Vitvi08g02180\_t001 |  | \| \| |  |  |  |  |  |  |  |  |  |  |
| 1 | Vvi-Vitvi08g04210\_t001 |  | \| \| |  |  |  |  |  |  |  |  |  |  |
| 1 | Vvi-Vitvi08g01144\_t001 |  | \| \| |  |  |  |  |  |  |  |  |  |  |
| 1 | Vvi-Vitvi08g01146\_t001 |  | \| \| |  |  |  |  |  |  |  |  |  |  |
| 1 | Vvi-Vitvi08g01147\_t001 |  | \| \| |  |  |  |  |  |  |  |  |  |  |
| 1 | Vvi-Vitvi08g01148\_t001 |  | \| \| |  |  |  |  |  |  |  |  |  |  |
| 1 | Vvi-Vitvi08g01149\_t002 |  | \| \| |  |  |  |  |  |  |  |  |  |  |
| 1 | Vvi-Vitvi08g04211\_t001 |  | \| \| |  |  |  |  |  |  |  |  |  |  |
| 1 | Vvi-Vitvi08g04212\_t001 |  | \| \| |  |  |  |  |  |  |  |  |  |  |
| 1 | Vvi-Vitvi08g01150\_t001 |  | \| \| |  |  |  |  |  |  |  |  |  |  |
| 1 | Vvi-Vitvi08g01152\_t003 |  | \| \| |  |  |  |  |  |  |  |  |  |  |
| 1 | Vvi-Vitvi08g01153\_t001 |  | Atr-ERM95711 |  |  |  |  |  |  |  |  |  |  |
| 0 | Vvi-Vitvi08g01154\_t001 |  |  |  |  |  |  |  |  |  |  |  |  |
| 0 | Vvi-Vitvi08g01155\_t001 |  |  |  |  |  |  |  |  |  |  |  |  |
| 0 | Vvi-Vitvi08g01156\_t001 |  |  |  |  |  |  |  |  |  |  |  |  |
| 0 | Vvi-Vitvi08g01158\_t001 |  |  |  |  |  |  |  |  |  |  |  |  |
| 0 | Vvi-Vitvi08g01159\_t001 |  |  |  |  |  |  |  |  |  |  |  |  |
| 1 | Vvi-Vitvi08g01161\_t003 |  | Atr-ERN07772 |  |  |  |  |  |  |  |  |  |  |
| 1 | Vvi-Vitvi08g01162\_t001 |  | Atr-ERN07771 |  |  |  |  |  |  |  |  |  |  |
| 1 | Vvi-Vitvi08g01163\_t002 |  | Atr-ERN07768 |  |  |  |  |  |  |  |  |  |  |
| 1 | Vvi-Vitvi08g01164\_t001 |  | Atr-ERN07767 |  |  |  |  |  |  |  |  |  |  |
| 1 | Vvi-Vitvi08g01165\_t001 |  | Atr-ERN07755 |  |  |  |  |  |  |  |  |  |  |
| 1 | Vvi-Vitvi08g01166\_t001 |  | Atr-ERN07753 |  |  |  |  |  |  |  |  |  |  |
| 1 | Vvi-Vitvi08g02183\_t001 |  | \| \| |  |  |  |  |  |  |  |  |  |  |
| 1 | Vvi-Vitvi08g01167\_t001 |  | \| \| |  |  |  |  |  |  |  |  |  |  |
| 1 | Vvi-Vitvi08g02184\_t001 |  | \| \| |  |  |  |  |  |  |  |  |  |  |
| 1 | Vvi-Vitvi08g01168\_t001 |  | Atr-ERN07746 |  |  |  |  |  |  |  |  |  |  |
| 1 | Vvi-Vitvi08g01171\_t001 |  | \| \| |  |  |  |  |  |  |  |  |  |  |
| 1 | Vvi-Vitvi08g01172\_t001 |  | \| \| |  |  |  |  |  |  |  |  |  |  |
| 1 | Vvi-Vitvi08g01174\_t001 |  | \| \| |  |  |  |  |  |  |  |  |  |  |
| 1 | Vvi-Vitvi08g04213\_t001 |  | \| \| |  |  |  |  |  |  |  |  |  |  |
| 1 | Vvi-Vitvi08g01176\_t001 |  | \| \| |  |  |  |  |  |  |  |  |  |  |
| 1 | Vvi-Vitvi08g02185\_t001 |  | \| \| |  |  |  |  |  |  |  |  |  |  |
| 1 | Vvi-Vitvi08g01177\_t001 |  | Atr-ERN07734 |  |  |  |  |  |  |  |  |  |  |
| 0 | Vvi-Vitvi08g02186\_t001 |  |  |  |  |  |  |  |  |  |  |  |  |
| 0 | Vvi-Vitvi08g01178\_t001 |  |  |  |  |  |  |  |  |  |  |  |  |
| 0 | Vvi-Vitvi08g04214\_t001 |  |  |  |  |  |  |  |  |  |  |  |  |
| 1 | Vvi-Vitvi08g01179\_t001 |  | Atr-ERN07814 |  |  |  |  |  |  |  |  |  |  |
| 1 | Vvi-Vitvi08g02188\_t001 |  | \| \| |  |  |  |  |  |  |  |  |  |  |
| 1 | Vvi-Vitvi08g01180\_t001 |  | \| \| |  |  |  |  |  |  |  |  |  |  |
| 1 | Vvi-Vitvi08g02189\_t001 |  | \| \| |  |  |  |  |  |  |  |  |  |  |
| 1 | Vvi-Vitvi08g01182\_t001 |  | \| \| |  |  |  |  |  |  |  |  |  |  |
| 1 | Vvi-Vitvi08g04215\_t001 |  | \| \| |  |  |  |  |  |  |  |  |  |  |
| 1 | Vvi-Vitvi08g04216\_t001 |  | \| \| |  |  |  |  |  |  |  |  |  |  |
| 1 | Vvi-Vitvi08g01184\_t001 |  | \| \| |  |  |  |  |  |  |  |  |  |  |
| 1 | Vvi-Vitvi08g01185\_t001 |  | \| \| |  |  |  |  |  |  |  |  |  |  |
| 1 | Vvi-Vitvi08g01186\_t001 |  | Atr-ERN07821 |  |  |  |  |  |  |  |  |  |  |
| 1 | Vvi-Vitvi08g02190\_t001 |  | \| \| |  |  |  |  |  |  |  |  |  |  |
| 1 | Vvi-Vitvi08g01188\_t001 |  | Atr-ERN07825 |  |  |  |  |  |  |  |  |  |  |
| 1 | Vvi-Vitvi08g02191\_t001 |  | Atr-ERN07829 |  |  |  |  |  |  |  |  |  |  |
| 1 | Vvi-Vitvi08g01189\_t001 |  | Atr-ERN07830 |  |  |  |  |  |  |  |  |  |  |
| 1 | Vvi-Vitvi08g04217\_t001 |  | \| \| |  |  |  |  |  |  |  |  |  |  |
| 1 | Vvi-Vitvi08g02192\_t001 |  | \| \| |  |  |  |  |  |  |  |  |  |  |
| 1 | Vvi-Vitvi08g04218\_t001 |  | \| \| |  |  |  |  |  |  |  |  |  |  |
| 1 | Vvi-Vitvi08g01192\_t001 |  | \| \| |  |  |  |  |  |  |  |  |  |  |
| 1 | Vvi-Vitvi08g01193\_t001 |  | Atr-ERN07834 |  |  |  |  |  |  |  |  |  |  |
| 1 | Vvi-Vitvi08g01194\_t001 |  | Atr-ERN07838 |  |  |  |  |  |  |  |  |  |  |
| 1 | Vvi-Vitvi08g01195\_t001 |  | Atr-ERN07841 |  |  |  |  |  |  |  |  |  |  |
| 1 | Vvi-Vitvi08g04219\_t001 |  | \| \| |  |  |  |  |  |  |  |  |  |  |
| 1 | Vvi-Vitvi08g01196\_t001 |  | \| \| |  |  |  |  |  |  |  |  |  |  |
| 1 | Vvi-Vitvi08g01197\_t001 |  | Atr-ERN07847 |  |  |  |  |  |  |  |  |  |  |
| 1 | Vvi-Vitvi08g02195\_t001 |  | Atr-ERN07849 |  |  |  |  |  |  |  |  |  |  |
| 1 | Vvi-Vitvi08g02196\_t001 |  | Atr-ERN07858 |  |  |  |  |  |  |  |  |  |  |
| 1 | Vvi-Vitvi08g01201\_t003 |  | Atr-ERN07860 |  |  |  |  |  |  |  |  |  |  |
| 1 | Vvi-Vitvi08g04220\_t001 |  | \| \| |  |  |  |  |  |  |  |  |  |  |
| 1 | Vvi-Vitvi08g04221\_t001 |  | \| \| |  |  |  |  |  |  |  |  |  |  |
| 1 | Vvi-Vitvi08g04222\_t001 |  | \| \| |  |  |  |  |  |  |  |  |  |  |
| 1 | Vvi-Vitvi08g01204\_t004 |  | Atr-ERN07877 |  |  |  |  |  |  |  |  |  |  |
| 1 | Vvi-Vitvi08g01205\_t001 |  | \| \| |  |  |  |  |  |  |  |  |  |  |
| 1 | Vvi-Vitvi08g01206\_t001 |  | Atr-ERN07898 |  |  |  |  |  |  |  |  |  |  |
| 1 | Vvi-Vitvi08g01208\_t001 |  | Atr-ERN07900 |  |  |  |  |  |  |  |  |  |  |
| 1 | Vvi-Vitvi08g01209\_t001 |  | Atr-ERN07902 |  |  |  |  |  |  |  |  |  |  |
| 1 | Vvi-Vitvi08g01211\_t001 |  | Atr-ERN07919 |  |  |  |  |  |  |  |  |  |  |
| 1 | Vvi-Vitvi08g01212\_t001 |  | \| \| |  |  |  |  |  |  |  |  |  |  |
| 1 | Vvi-Vitvi08g01213\_t002 |  | Atr-ERN07920 |  |  |  |  |  |  |  |  |  |  |
| 1 | Vvi-Vitvi08g04223\_t001 |  | \| \| |  |  |  |  |  |  |  |  |  |  |
| 1 | Vvi-Vitvi08g01214\_t001 |  | Atr-ERN07921 |  |  |  |  |  |  |  |  |  |  |
| 1 | Vvi-Vitvi08g01215\_t001 |  | Atr-ERN07923 |  |  |  |  |  |  |  |  |  |  |
| 1 | Vvi-Vitvi08g01216\_t001 |  | Atr-ERN07928 |  |  |  |  |  |  |  |  |  |  |
| 1 | Vvi-Vitvi08g01217\_t001 |  | Atr-ERN07931 |  |  |  |  |  |  |  |  |  |  |
| 1 | Vvi-Vitvi08g01218\_t001 |  | \| \| |  |  |  |  |  |  |  |  |  |  |
| 1 | Vvi-Vitvi08g04224\_t001 |  | \| \| |  |  |  |  |  |  |  |  |  |  |
| 1 | Vvi-Vitvi08g04225\_t001 |  | \| \| |  |  |  |  |  |  |  |  |  |  |
| 1 | Vvi-Vitvi08g04226\_t001 |  | \| \| |  |  |  |  |  |  |  |  |  |  |
| 1 | Vvi-Vitvi08g01221\_t001 |  | Atr-ERN07935 |  |  |  |  |  |  |  |  |  |  |
| 1 | Vvi-Vitvi08g01222\_t001 |  | \| \| |  |  |  |  |  |  |  |  |  |  |
| 1 | Vvi-Vitvi08g01223\_t001 |  | Atr-ERN07940 |  |  |  |  |  |  |  |  |  |  |
| 1 | Vvi-Vitvi08g04227\_t001 |  | \| \| |  |  |  |  |  |  |  |  |  |  |
| 1 | Vvi-Vitvi08g02198\_t001 |  | \| \| |  |  |  |  |  |  |  |  |  |  |
| 1 | Vvi-Vitvi08g01224\_t001 |  | Atr-ERN07948 |  |  |  |  |  |  |  |  |  |  |
| 1 | Vvi-Vitvi08g04228\_t001 |  | \| \| |  |  |  |  |  |  |  |  |  |  |
| 1 | Vvi-Vitvi08g04229\_t001 |  | \| \| |  |  |  |  |  |  |  |  |  |  |
| 1 | Vvi-Vitvi08g04230\_t001 |  | \| \| |  |  |  |  |  |  |  |  |  |  |
| 1 | Vvi-Vitvi08g04231\_t001 |  | \| \| |  |  |  |  |  |  |  |  |  |  |
| 1 | Vvi-Vitvi08g04232\_t001 |  | \| \| |  |  |  |  |  |  |  |  |  |  |
| 1 | Vvi-Vitvi08g02200\_t001 |  | \| \| |  |  |  |  |  |  |  |  |  |  |
| 1 | Vvi-Vitvi08g04233\_t001 |  | Atr-ERN07950 |  |  |  |  |  |  |  |  |  |  |
| 1 | Vvi-Vitvi08g02201\_t001 |  | \| \| |  |  |  |  |  |  |  |  |  |  |
| 1 | Vvi-Vitvi08g01228\_t001 |  | \| \| |  |  |  |  |  |  |  |  |  |  |
| 1 | Vvi-Vitvi08g04234\_t001 |  | \| \| |  |  |  |  |  |  |  |  |  |  |
| 1 | Vvi-Vitvi08g01229\_t001 |  | \| \| |  |  |  |  |  |  |  |  |  |  |
| 1 | Vvi-Vitvi08g01230\_t001 |  | Atr-ERN07955 |  |  |  |  |  |  |  |  |  |  |
| 1 | Vvi-Vitvi08g01231\_t001 |  | Atr-ERN07958 |  |  |  |  |  |  |  |  |  |  |
| 1 | Vvi-Vitvi08g01232\_t001 |  | \| \| |  |  |  |  |  |  |  |  |  |  |
| 1 | Vvi-Vitvi08g01233\_t001 |  | \| \| |  |  |  |  |  |  |  |  |  |  |
| 1 | Vvi-Vitvi08g01234\_t001 |  | Atr-ERN07965 |  |  |  |  |  |  |  |  |  |  |
| 1 | Vvi-Vitvi08g01235\_t001 |  | Atr-ERN07967 |  |  |  |  |  |  |  |  |  |  |
| 1 | Vvi-Vitvi08g01236\_t001 |  | \| \| |  |  |  |  |  |  |  |  |  |  |
| 1 | Vvi-Vitvi08g01237\_t001 |  | Atr-ERN07970 |  |  |  |  |  |  |  |  |  |  |
| 1 | Vvi-Vitvi08g01238\_t001 |  | Atr-ERN07973 |  |  |  |  |  |  |  |  |  |  |
| 1 | Vvi-Vitvi08g04235\_t001 |  | \| \| |  |  |  |  |  |  |  |  |  |  |
| 1 | Vvi-Vitvi08g01239\_t001 |  | Atr-ERN07975 |  |  |  |  |  |  |  |  |  |  |
| 1 | Vvi-Vitvi08g02202\_t001 |  | Atr-ERN07978 |  |  |  |  |  |  |  |  |  |  |
| 1 | Vvi-Vitvi08g01240\_t001 |  | Atr-ERN07979 |  |  |  |  |  |  |  |  |  |  |
| 1 | Vvi-Vitvi08g01241\_t001 |  | Atr-ERN07982 |  |  |  |  |  |  |  |  |  |  |
| 1 | Vvi-Vitvi08g01242\_t001 |  | \| \| |  |  |  |  |  |  |  |  |  |  |
| 1 | Vvi-Vitvi08g01243\_t001 |  | Atr-ERN07985 |  |  |  |  |  |  |  |  |  |  |
| 1 | Vvi-Vitvi08g04236\_t001 |  | \| \| |  |  |  |  |  |  |  |  |  |  |
| 1 | Vvi-Vitvi08g01245\_t001 |  | Atr-ERN08004 |  |  |  |  |  |  |  |  |  |  |
| 1 | Vvi-Vitvi08g01246\_t001 |  | \| \| |  |  |  |  |  |  |  |  |  |  |
| 1 | Vvi-Vitvi08g01247\_t001 |  | Atr-ERN08008 |  |  |  |  |  |  |  |  |  |  |
| 1 | Vvi-Vitvi08g01248\_t001 |  | Atr-ERN08018 |  |  |  |  |  |  |  |  |  |  |
| 1 | Vvi-Vitvi08g02203\_t001 |  | Atr-ERN08020 |  |  |  |  |  |  |  |  |  |  |
| 1 | Vvi-Vitvi08g01249\_t001 |  | \| \| |  |  |  |  |  |  |  |  |  |  |
| 1 | Vvi-Vitvi08g01250\_t001 |  | Atr-ERN08026 |  |  |  |  |  |  |  |  |  |  |
| 1 | Vvi-Vitvi08g04237\_t001 |  | \| \| |  |  |  |  |  |  |  |  |  |  |
| 1 | Vvi-Vitvi08g01252\_t001 |  | \| \| |  |  |  |  |  |  |  |  |  |  |
| 1 | Vvi-Vitvi08g04238\_t001 |  | Atr-ERN08028 |  |  |  |  |  |  |  |  |  |  |
| 1 | Vvi-Vitvi08g02204\_t001 |  | Atr-ERN08033 |  |  |  |  |  |  |  |  |  |  |
| 1 | Vvi-Vitvi08g01253\_t001 |  | \| \| |  |  |  |  |  |  |  |  |  |  |
| 1 | Vvi-Vitvi08g02205\_t001 |  | Atr-ERN08038 |  |  |  |  |  |  |  |  |  |  |
| 1 | Vvi-Vitvi08g01254\_t002 |  | Atr-ERN08039 |  |  |  |  |  |  |  |  |  |  |
| 1 | Vvi-Vitvi08g04239\_t001 |  | \| \| |  |  |  |  |  |  |  |  |  |  |
| 1 | Vvi-Vitvi08g02206\_t001 |  | \| \| |  |  |  |  |  |  |  |  |  |  |
| 1 | Vvi-Vitvi08g01255\_t001 |  | \| \| |  |  |  |  |  |  |  |  |  |  |
| 1 | Vvi-Vitvi08g04240\_t001 |  | \| \| |  |  |  |  |  |  |  |  |  |  |
| 1 | Vvi-Vitvi08g01256\_t001 |  | \| \| |  |  |  |  |  |  |  |  |  |  |
| 1 | Vvi-Vitvi08g01257\_t001 |  | Atr-ERN08048 |  |  |  |  |  |  |  |  |  |  |
| 1 | Vvi-Vitvi08g04241\_t001 |  | Atr-ERN08050 |  |  |  |  |  |  |  |  |  |  |
| 1 | Vvi-Vitvi08g01259\_t001 |  | \| \| |  |  |  |  |  |  |  |  |  |  |
| 1 | Vvi-Vitvi08g02209\_t001 |  | \| \| |  |  |  |  |  |  |  |  |  |  |
| 1 | Vvi-Vitvi08g02210\_t001 |  | \| \| |  |  |  |  |  |  |  |  |  |  |
| 1 | Vvi-Vitvi08g01260\_t001 |  | \| \| |  |  |  |  |  |  |  |  |  |  |
| 1 | Vvi-Vitvi08g01261\_t001 |  | Atr-ERN08053 |  |  |  |  |  |  |  |  |  |  |
| 1 | Vvi-Vitvi08g01262\_t001 |  | Atr-ERN08057 |  |  |  |  |  |  |  |  |  |  |
| 1 | Vvi-Vitvi08g04242\_t001 |  | \| \| |  |  |  |  |  |  |  |  |  |  |
| 1 | Vvi-Vitvi08g01264\_t001 |  | \| \| |  |  |  |  |  |  |  |  |  |  |
| 1 | Vvi-Vitvi08g04243\_t001 |  | \| \| |  |  |  |  |  |  |  |  |  |  |
| 1 | Vvi-Vitvi08g01265\_t001 |  | Atr-ERN08059 |  |  |  |  |  |  |  |  |  |  |
| 1 | Vvi-Vitvi08g02211\_t001 |  | \| \| |  |  |  |  |  |  |  |  |  |  |
| 1 | Vvi-Vitvi08g02212\_t001 |  | \| \| |  |  |  |  |  |  |  |  |  |  |
| 1 | Vvi-Vitvi08g04244\_t001 |  | \| \| |  |  |  |  |  |  |  |  |  |  |
| 1 | Vvi-Vitvi08g01266\_t002 |  | Atr-ERN08071 |  |  |  |  |  |  |  |  |  |  |
| 1 | Vvi-Vitvi08g01267\_t001 |  | Atr-ERN08074 |  |  |  |  |  |  |  |  |  |  |
| 1 | Vvi-Vitvi08g02214\_t001 |  | Atr-ERN08078 |  |  |  |  |  |  |  |  |  |  |
| 1 | Vvi-Vitvi08g04245\_t001 |  | \| \| |  |  |  |  |  |  |  |  |  |  |
| 1 | Vvi-Vitvi08g04246\_t001 |  | \| \| |  |  |  |  |  |  |  |  |  |  |
| 1 | Vvi-Vitvi08g02216\_t001 |  | \| \| |  |  |  |  |  |  |  |  |  |  |
| 1 | Vvi-Vitvi08g04247\_t001 |  | \| \| |  |  |  |  |  |  |  |  |  |  |
| 1 | Vvi-Vitvi08g04248\_t001 |  | \| \| |  |  |  |  |  |  |  |  |  |  |
| 1 | Vvi-Vitvi08g02217\_t001 |  | \| \| |  |  |  |  |  |  |  |  |  |  |
| 1 | Vvi-Vitvi08g02218\_t001 |  | \| \| |  |  |  |  |  |  |  |  |  |  |
| 1 | Vvi-Vitvi08g04249\_t001 |  | \| \| |  |  |  |  |  |  |  |  |  |  |
| 1 | Vvi-Vitvi08g04250\_t001 |  | \| \| |  |  |  |  |  |  |  |  |  |  |
| 1 | Vvi-Vitvi08g02220\_t001 |  | \| \| |  |  |  |  |  |  |  |  |  |  |
| 1 | Vvi-Vitvi08g02221\_t001 |  | \| \| |  |  |  |  |  |  |  |  |  |  |
| 1 | Vvi-Vitvi08g01270\_t001 |  | Atr-ERN08080 |  |  |  |  |  |  |  |  |  |  |
| 1 | Vvi-Vitvi08g01272\_t001 |  | Atr-ERN08085 |  |  |  |  |  |  |  |  |  |  |
| 0 | Vvi-Vitvi08g02222\_t001 |  |  |  |  |  |  |  |  |  |  |  |  |
| 1 | Vvi-Vitvi08g01273\_t001 |  | Atr-ERN12027 |  |  |  |  |  |  |  |  |  |  |
| 1 | Vvi-Vitvi08g01274\_t001 |  | \| \| |  |  |  |  |  |  |  |  |  |  |
| 1 | Vvi-Vitvi08g01276\_t001 |  | \| \| |  |  |  |  |  |  |  |  |  |  |
| 1 | Vvi-Vitvi08g04251\_t001 |  | Atr-ERN12019 |  |  |  |  |  |  |  |  |  |  |
| 1 | Vvi-Vitvi08g01278\_t001 |  | \| \| |  |  |  |  |  |  |  |  |  |  |
| 1 | Vvi-Vitvi08g02223\_t001 |  | \| \| |  |  |  |  |  |  |  |  |  |  |
| 1 | Vvi-Vitvi08g01280\_t001 |  | \| \| |  |  |  |  |  |  |  |  |  |  |
| 1 | Vvi-Vitvi08g04252\_t001 |  | \| \| |  |  |  |  |  |  |  |  |  |  |
| 1 | Vvi-Vitvi08g04253\_t006 |  | \| \| |  |  |  |  |  |  |  |  |  |  |
| 1 | Vvi-Vitvi08g01281\_t001 |  | Atr-ERN12011 |  |  |  |  |  |  |  |  |  |  |
| 1 | Vvi-Vitvi08g01282\_t001 |  | Atr-ERN12008 |  |  |  |  |  |  |  |  |  |  |
| 1 | Vvi-Vitvi08g01283\_t001 |  | \| \| |  |  |  |  |  |  |  |  |  |  |
| 1 | Vvi-Vitvi08g02226\_t002 |  | \| \| |  |  |  |  |  |  |  |  |  |  |
| 1 | Vvi-Vitvi08g02228\_t001 |  | \| \| |  |  |  |  |  |  |  |  |  |  |
| 1 | Vvi-Vitvi08g04254\_t001 |  | \| \| |  |  |  |  |  |  |  |  |  |  |
| 1 | Vvi-Vitvi08g01285\_t001 |  | Atr-ERN12005 |  |  |  |  |  |  |  |  |  |  |
| 1 | Vvi-Vitvi08g01287\_t001 |  | Atr-ERN12003 |  |  |  |  |  |  |  |  |  |  |
| 1 | Vvi-Vitvi08g01288\_t002 |  | Atr-ERN12002 |  |  |  |  |  |  |  |  |  |  |
| 1 | Vvi-Vitvi08g02230\_t001 |  | Atr-ERN12000 |  |  |  |  |  |  |  |  |  |  |
| 1 | Vvi-Vitvi08g04255\_t001 |  | \| \| |  |  |  |  |  |  |  |  |  |  |
| 1 | Vvi-Vitvi08g01290\_t001 |  | \| \| |  |  |  |  |  |  |  |  |  |  |
| 1 | Vvi-Vitvi08g01291\_t001 |  | Atr-ERN11999 |  |  |  |  |  |  |  |  |  |  |
| 1 | Vvi-Vitvi08g04256\_t001 |  | \| \| |  |  |  |  |  |  |  |  |  |  |
| 1 | Vvi-Vitvi08g01292\_t001 |  | Atr-ERN11990 |  |  |  |  |  |  |  |  |  |  |
| 1 | Vvi-Vitvi08g04257\_t001 |  | \| \| |  |  |  |  |  |  |  |  |  |  |
| 1 | Vvi-Vitvi08g04258\_t001 |  | \| \| |  |  |  |  |  |  |  |  |  |  |
| 2 | Vvi-Vitvi08g01293\_t001 |  | Atr-ERN11984 |  | Atr-ERM95074 |  |  |  |  |  |  |  |  |
| 1 | Vvi-Vitvi08g01294\_t001 |  |  |  | \| \| |  |  |  |  |  |  |  |  |
| 2 | Vvi-Vitvi08g01295\_t001 |  | Atr-ERN09611 |  | Atr-ERM95076 |  |  |  |  |  |  |  |  |
| 2 | Vvi-Vitvi08g01296\_t001 |  | Atr-ERN09612 |  | Atr-ERM95078 |  |  |  |  |  |  |  |  |
| 2 | Vvi-Vitvi08g01297\_t001 |  | Atr-ERN09613 |  | Atr-ERM95080 |  |  |  |  |  |  |  |  |
| 2 | Vvi-Vitvi08g04259\_t001 |  | \| \| |  | \| \| |  |  |  |  |  |  |  |  |
| 2 | Vvi-Vitvi08g01298\_t001 |  | Atr-ERN09614 |  | \| \| |  |  |  |  |  |  |  |  |
| 2 | Vvi-Vitvi08g01299\_t001 |  | Atr-ERN09615 |  | \| \| |  |  |  |  |  |  |  |  |
| 2 | Vvi-Vitvi08g01300\_t001 |  | Atr-ERN09616 |  | Atr-ERM95083 |  |  |  |  |  |  |  |  |
| 2 | Vvi-Vitvi08g01301\_t001 |  | Atr-ERN09618 |  | \| \| |  |  |  |  |  |  |  |  |
| 2 | Vvi-Vitvi08g04260\_t001 |  | \| \| |  | \| \| |  |  |  |  |  |  |  |  |
| 2 | Vvi-Vitvi08g01303\_t001 |  | Atr-ERN09619 |  | \| \| |  |  |  |  |  |  |  |  |
| 2 | Vvi-Vitvi08g01304\_t001 |  | Atr-ERN09622 |  | \| \| |  |  |  |  |  |  |  |  |
| 2 | Vvi-Vitvi08g04261\_t001 |  | \| \| |  | \| \| |  |  |  |  |  |  |  |  |
| 2 | Vvi-Vitvi08g01305\_t001 |  | Atr-ERN09624 |  | \| \| |  |  |  |  |  |  |  |  |
| 2 | Vvi-Vitvi08g02232\_t002 |  | Atr-ERN09625 |  | Atr-ERM95096 |  |  |  |  |  |  |  |  |
| 2 | Vvi-Vitvi08g01307\_t001 |  | \| \| |  | \| \| |  |  |  |  |  |  |  |  |
| 2 | Vvi-Vitvi08g02233\_t001 |  | Atr-ERN09630 |  | \| \| |  |  |  |  |  |  |  |  |
| 2 | Vvi-Vitvi08g04262\_t001 |  | \| \| |  | \| \| |  |  |  |  |  |  |  |  |
| 2 | Vvi-Vitvi08g01308\_t001 |  | Atr-ERN09632 |  | \| \| |  |  |  |  |  |  |  |  |
| 2 | Vvi-Vitvi08g01309\_t002 |  | Atr-ERN09634 |  | \| \| |  |  |  |  |  |  |  |  |
| 2 | Vvi-Vitvi08g04263\_t001 |  | Atr-ERN09635 |  | \| \| |  |  |  |  |  |  |  |  |
| 2 | Vvi-Vitvi08g04264\_t001 |  | \| \| |  | \| \| |  |  |  |  |  |  |  |  |
| 2 | Vvi-Vitvi08g01311\_t001 |  | Atr-ERN09637 |  | \| \| |  |  |  |  |  |  |  |  |
| 2 | Vvi-Vitvi08g04265\_t001 |  | \| \| |  | \| \| |  |  |  |  |  |  |  |  |
| 2 | Vvi-Vitvi08g01312\_t001 |  | \| \| |  | \| \| |  |  |  |  |  |  |  |  |
| 2 | Vvi-Vitvi08g01313\_t001 |  | Atr-ERN09651 |  | \| \| |  |  |  |  |  |  |  |  |
| 2 | Vvi-Vitvi08g04266\_t001 |  | \| \| |  | \| \| |  |  |  |  |  |  |  |  |
| 2 | Vvi-Vitvi08g01314\_t001 |  | \| \| |  | \| \| |  |  |  |  |  |  |  |  |
| 2 | Vvi-Vitvi08g01315\_t001 |  | Atr-ERN09653 |  | \| \| |  |  |  |  |  |  |  |  |
| 2 | Vvi-Vitvi08g01316\_t001 |  | Atr-ERN09654 |  | \| \| |  |  |  |  |  |  |  |  |
| 2 | Vvi-Vitvi08g01318\_t001 |  | Atr-ERN09657 |  | \| \| |  |  |  |  |  |  |  |  |
| 2 | Vvi-Vitvi08g01319\_t001 |  | Atr-ERN09661 |  | Atr-ERM95113 |  |  |  |  |  |  |  |  |
| 1 | Vvi-Vitvi08g01320\_t001 |  | Atr-ERN09664 |  |  |  |  |  |  |  |  |  |  |
| 1 | Vvi-Vitvi08g01321\_t001 |  | \| \| |  |  |  |  |  |  |  |  |  |  |
| 1 | Vvi-Vitvi08g01322\_t001 |  | \| \| |  |  |  |  |  |  |  |  |  |  |
| 1 | Vvi-Vitvi08g01323\_t001 |  | Atr-ERN09669 |  |  |  |  |  |  |  |  |  |  |
| 1 | Vvi-Vitvi08g04267\_t001 |  | Atr-ERN09670 |  |  |  |  |  |  |  |  |  |  |
| 1 | Vvi-Vitvi08g01325\_t001 |  | Atr-ERN09673 |  |  |  |  |  |  |  |  |  |  |
| 1 | Vvi-Vitvi08g01326\_t001 |  | Atr-ERN09679 |  |  |  |  |  |  |  |  |  |  |
| 1 | Vvi-Vitvi08g01327\_t001 |  | Atr-ERN09683 |  |  |  |  |  |  |  |  |  |  |
| 1 | Vvi-Vitvi08g04268\_t001 |  | \| \| |  |  |  |  |  |  |  |  |  |  |
| 1 | Vvi-Vitvi08g01329\_t001 |  | Atr-ERN09684 |  |  |  |  |  |  |  |  |  |  |
| 1 | Vvi-Vitvi08g04269\_t001 |  | \| \| |  |  |  |  |  |  |  |  |  |  |
| 1 | Vvi-Vitvi08g04270\_t001 |  | \| \| |  |  |  |  |  |  |  |  |  |  |
| 1 | Vvi-Vitvi08g04271\_t001 |  | Atr-ERN09685 |  |  |  |  |  |  |  |  |  |  |
| 1 | Vvi-Vitvi08g04272\_t001 |  | \| \| |  |  |  |  |  |  |  |  |  |  |
| 1 | Vvi-Vitvi08g01332\_t001 |  | Atr-ERN09686 |  |  |  |  |  |  |  |  |  |  |
| 1 | Vvi-Vitvi08g01333\_t001 |  | Atr-ERN09688 |  |  |  |  |  |  |  |  |  |  |
| 1 | Vvi-Vitvi08g04273\_t001 |  | \| \| |  |  |  |  |  |  |  |  |  |  |
| 1 | Vvi-Vitvi08g01334\_t001 |  | \| \| |  |  |  |  |  |  |  |  |  |  |
| 1 | Vvi-Vitvi08g01335\_t001 |  | \| \| |  |  |  |  |  |  |  |  |  |  |
| 1 | Vvi-Vitvi08g01336\_t001 |  | Atr-ERN09692 |  |  |  |  |  |  |  |  |  |  |
| 1 | Vvi-Vitvi08g01337\_t001 |  | Atr-ERN09709 |  |  |  |  |  |  |  |  |  |  |
| 1 | Vvi-Vitvi08g01338\_t001 |  | Atr-ERN09711 |  |  |  |  |  |  |  |  |  |  |
| 1 | Vvi-Vitvi08g02235\_t001 |  | \| \| |  |  |  |  |  |  |  |  |  |  |
| 1 | Vvi-Vitvi08g01339\_t001 |  | Atr-ERN09713 |  |  |  |  |  |  |  |  |  |  |
| 1 | Vvi-Vitvi08g02236\_t001 |  | \| \| |  |  |  |  |  |  |  |  |  |  |
| 1 | Vvi-Vitvi08g01340\_t001 |  | Atr-ERN09715 |  |  |  |  |  |  |  |  |  |  |
| 1 | Vvi-Vitvi08g04274\_t001 |  | \| \| |  |  |  |  |  |  |  |  |  |  |
| 1 | Vvi-Vitvi08g04275\_t001 |  | \| \| |  |  |  |  |  |  |  |  |  |  |
| 1 | Vvi-Vitvi08g01341\_t001 |  | Atr-ERN09719 |  |  |  |  |  |  |  |  |  |  |
| 1 | Vvi-Vitvi08g01342\_t001 |  | Atr-ERN09725 |  |  |  |  |  |  |  |  |  |  |
| 1 | Vvi-Vitvi08g04276\_t001 |  | \| \| |  |  |  |  |  |  |  |  |  |  |
| 1 | Vvi-Vitvi08g04277\_t001 |  | \| \| |  |  |  |  |  |  |  |  |  |  |
| 1 | Vvi-Vitvi08g02238\_t001 |  | \| \| |  |  |  |  |  |  |  |  |  |  |
| 1 | Vvi-Vitvi08g04278\_t001 |  | \| \| |  |  |  |  |  |  |  |  |  |  |
| 1 | Vvi-Vitvi08g01346\_t001 |  | Atr-ERN09739 |  |  |  |  |  |  |  |  |  |  |
| 1 | Vvi-Vitvi08g01347\_t001 |  | \| \| |  |  |  |  |  |  |  |  |  |  |
| 1 | Vvi-Vitvi08g01348\_t001 |  | \| \| |  |  |  |  |  |  |  |  |  |  |
| 1 | Vvi-Vitvi08g02239\_t001 |  | \| \| |  |  |  |  |  |  |  |  |  |  |
| 1 | Vvi-Vitvi08g04279\_t001 |  | \| \| |  |  |  |  |  |  |  |  |  |  |
| 1 | Vvi-Vitvi08g02241\_t001 |  | \| \| |  |  |  |  |  |  |  |  |  |  |
| 1 | Vvi-Vitvi08g02242\_t001 |  | \| \| |  |  |  |  |  |  |  |  |  |  |
| 1 | Vvi-Vitvi08g02243\_t002 |  | Atr-ERN09755 |  |  |  |  |  |  |  |  |  |  |
| 1 | Vvi-Vitvi08g01349\_t001 |  | Atr-ERN09757 |  |  |  |  |  |  |  |  |  |  |
| 1 | Vvi-Vitvi08g01351\_t001 |  | \| \| |  |  |  |  |  |  |  |  |  |  |
| 1 | Vvi-Vitvi08g01352\_t001 |  | Atr-ERN09758 |  |  |  |  |  |  |  |  |  |  |
| 1 | Vvi-Vitvi08g01353\_t001 |  | Atr-ERN09763 |  |  |  |  |  |  |  |  |  |  |
| 1 | Vvi-Vitvi08g01354\_t001 |  | Atr-ERN09764 |  |  |  |  |  |  |  |  |  |  |
| 1 | Vvi-Vitvi08g01355\_t001 |  | Atr-ERN09766 |  |  |  |  |  |  |  |  |  |  |
| 1 | Vvi-Vitvi08g01356\_t001 |  | Atr-ERN09767 |  |  |  |  |  |  |  |  |  |  |
| 1 | Vvi-Vitvi08g01357\_t001 |  | Atr-ERN09769 |  |  |  |  |  |  |  |  |  |  |
| 1 | Vvi-Vitvi08g04280\_t001 |  | \| \| |  |  |  |  |  |  |  |  |  |  |
| 1 | Vvi-Vitvi08g01358\_t001 |  | Atr-ERN09772 |  |  |  |  |  |  |  |  |  |  |
| 1 | Vvi-Vitvi08g01359\_t003 |  | Atr-ERN09773 |  |  |  |  |  |  |  |  |  |  |
| 1 | Vvi-Vitvi08g01360\_t001 |  | Atr-ERN09774 |  |  |  |  |  |  |  |  |  |  |
| 1 | Vvi-Vitvi08g04281\_t001 |  | \| \| |  |  |  |  |  |  |  |  |  |  |
| 1 | Vvi-Vitvi08g02244\_t003 |  | Atr-ERN09781 |  |  |  |  |  |  |  |  |  |  |
| 1 | Vvi-Vitvi08g01361\_t006 |  | Atr-ERN09783 |  |  |  |  |  |  |  |  |  |  |
| 1 | Vvi-Vitvi08g01363\_t001 |  | Atr-ERN09793 |  |  |  |  |  |  |  |  |  |  |
| 1 | Vvi-Vitvi08g01364\_t001 |  | Atr-ERN09795 |  |  |  |  |  |  |  |  |  |  |
| 1 | Vvi-Vitvi08g02245\_t002 |  | Atr-ERN09796 |  |  |  |  |  |  |  |  |  |  |
| 1 | Vvi-Vitvi08g04282\_t001 |  | \| \| |  |  |  |  |  |  |  |  |  |  |
| 1 | Vvi-Vitvi08g01365\_t001 |  | Atr-ERN09799 |  |  |  |  |  |  |  |  |  |  |
| 1 | Vvi-Vitvi08g01366\_t001 |  | Atr-ERN09801 |  |  |  |  |  |  |  |  |  |  |
| 1 | Vvi-Vitvi08g01367\_t001 |  | Atr-ERN09802 |  |  |  |  |  |  |  |  |  |  |
| 0 | Vvi-Vitvi08g01369\_t001 |  |  |  |  |  |  |  |  |  |  |  |  |
| 1 | Vvi-Vitvi08g01370\_t001 |  | Atr-ERN16106 |  |  |  |  |  |  |  |  |  |  |
| 1 | Vvi-Vitvi08g01371\_t001 |  | Atr-ERN16111 |  |  |  |  |  |  |  |  |  |  |
| 1 | Vvi-Vitvi08g01372\_t001 |  | \| \| |  |  |  |  |  |  |  |  |  |  |
| 1 | Vvi-Vitvi08g01373\_t001 |  | Atr-ERN16136 |  |  |  |  |  |  |  |  |  |  |
| 1 | Vvi-Vitvi08g01374\_t001 |  | Atr-ERN16137 |  |  |  |  |  |  |  |  |  |  |
| 1 | Vvi-Vitvi08g01375\_t001 |  | Atr-ERN16141 |  |  |  |  |  |  |  |  |  |  |
| 1 | Vvi-Vitvi08g04283\_t001 |  | \| \| |  |  |  |  |  |  |  |  |  |  |
| 1 | Vvi-Vitvi08g01376\_t001 |  | Atr-ERN16143 |  |  |  |  |  |  |  |  |  |  |
| 1 | Vvi-Vitvi08g01377\_t001 |  | Atr-ERN16146 |  |  |  |  |  |  |  |  |  |  |
| 1 | Vvi-Vitvi08g02246\_t001 |  | Atr-ERN16151 |  |  |  |  |  |  |  |  |  |  |
| 1 | Vvi-Vitvi08g01378\_t001 |  | Atr-ERN16155 |  |  |  |  |  |  |  |  |  |  |
| 1 | Vvi-Vitvi08g01379\_t001 |  | Atr-ERN16157 |  |  |  |  |  |  |  |  |  |  |
| 1 | Vvi-Vitvi08g04284\_t001 |  | Atr-ERN16159 |  |  |  |  |  |  |  |  |  |  |
| 1 | Vvi-Vitvi08g04285\_t001 |  | \| \| |  |  |  |  |  |  |  |  |  |  |
| 1 | Vvi-Vitvi08g04286\_t001 |  | \| \| |  |  |  |  |  |  |  |  |  |  |
| 1 | Vvi-Vitvi08g04287\_t001 |  | \| \| |  |  |  |  |  |  |  |  |  |  |
| 1 | Vvi-Vitvi08g04288\_t001 |  | \| \| |  |  |  |  |  |  |  |  |  |  |
| 1 | Vvi-Vitvi08g02249\_t001 |  | \| \| |  |  |  |  |  |  |  |  |  |  |
| 1 | Vvi-Vitvi08g04289\_t001 |  | \| \| |  |  |  |  |  |  |  |  |  |  |
| 1 | Vvi-Vitvi08g04290\_t001 |  | \| \| |  |  |  |  |  |  |  |  |  |  |
| 1 | Vvi-Vitvi08g01382\_t001 |  | \| \| |  |  |  |  |  |  |  |  |  |  |
| 1 | Vvi-Vitvi08g04291\_t001 |  | \| \| |  |  |  |  |  |  |  |  |  |  |
| 1 | Vvi-Vitvi08g01384\_t001 |  | \| \| |  |  |  |  |  |  |  |  |  |  |
| 1 | Vvi-Vitvi08g01385\_t001 |  | Atr-ERN16160 |  |  |  |  |  |  |  |  |  |  |
| 1 | Vvi-Vitvi08g04292\_t001 |  | \| \| |  |  |  |  |  |  |  |  |  |  |
| 1 | Vvi-Vitvi08g01386\_t001 |  | Atr-ERN16166 |  |  |  |  |  |  |  |  |  |  |
| 1 | Vvi-Vitvi08g01387\_t001 |  | Atr-ERN16168 |  |  |  |  |  |  |  |  |  |  |
| 1 | Vvi-Vitvi08g01388\_t001 |  | Atr-ERN16172 |  |  |  |  |  |  |  |  |  |  |
| 1 | Vvi-Vitvi08g01389\_t001 |  | Atr-ERN16176 |  |  |  |  |  |  |  |  |  |  |
| 1 | Vvi-Vitvi08g02251\_t001.1.6037826f |  | Atr-ERN16177 |  |  |  |  |  |  |  |  |  |  |
| 1 | Vvi-Vitvi08g01390\_t001 |  | Atr-ERN16179 |  |  |  |  |  |  |  |  |  |  |
| 1 | Vvi-Vitvi08g01391\_t001 |  | Atr-ERN16181 |  |  |  |  |  |  |  |  |  |  |
| 1 | Vvi-Vitvi08g01392\_t001 |  | Atr-ERN16183 |  |  |  |  |  |  |  |  |  |  |
| 1 | Vvi-Vitvi08g01393\_t001 |  | Atr-ERN16186 |  |  |  |  |  |  |  |  |  |  |
| 1 | Vvi-Vitvi08g01394\_t001 |  | Atr-ERN16189 |  |  |  |  |  |  |  |  |  |  |
| 1 | Vvi-Vitvi08g02252\_t001 |  | Atr-ERN16190 |  |  |  |  |  |  |  |  |  |  |
| 1 | Vvi-Vitvi08g01395\_t001 |  | Atr-ERN16191 |  |  |  |  |  |  |  |  |  |  |
| 1 | Vvi-Vitvi08g01396\_t001 |  | Atr-ERN16196 |  |  |  |  |  |  |  |  |  |  |
| 1 | Vvi-Vitvi08g01397\_t001 |  | Atr-ERN16202 |  |  |  |  |  |  |  |  |  |  |
| 1 | Vvi-Vitvi08g02253\_t001 |  | Atr-ERN16204 |  |  |  |  |  |  |  |  |  |  |
| 1 | Vvi-Vitvi08g02254\_t001 |  | \| \| |  |  |  |  |  |  |  |  |  |  |
| 1 | Vvi-Vitvi08g04293\_t001 |  | \| \| |  |  |  |  |  |  |  |  |  |  |
| 1 | Vvi-Vitvi08g04294\_t001 |  | \| \| |  |  |  |  |  |  |  |  |  |  |
| 1 | Vvi-Vitvi08g04295\_t001 |  | \| \| |  |  |  |  |  |  |  |  |  |  |
| 1 | Vvi-Vitvi08g01399\_t001 |  | \| \| |  |  |  |  |  |  |  |  |  |  |
| 1 | Vvi-Vitvi08g02255\_t001 |  | \| \| |  |  |  |  |  |  |  |  |  |  |
| 1 | Vvi-Vitvi08g01401\_t001 |  | \| \| |  |  |  |  |  |  |  |  |  |  |
| 1 | Vvi-Vitvi08g04296\_t001 |  | \| \| |  |  |  |  |  |  |  |  |  |  |
| 1 | Vvi-Vitvi08g01402\_t001 |  | \| \| |  |  |  |  |  |  |  |  |  |  |
| 1 | Vvi-Vitvi08g01403\_t001 |  | \| \| |  |  |  |  |  |  |  |  |  |  |
| 1 | Vvi-Vitvi08g04297\_t001 |  | \| \| |  |  |  |  |  |  |  |  |  |  |
| 1 | Vvi-Vitvi08g04298\_t001 |  | \| \| |  |  |  |  |  |  |  |  |  |  |
| 1 | Vvi-Vitvi08g04299\_t001 |  | \| \| |  |  |  |  |  |  |  |  |  |  |
| 1 | Vvi-Vitvi08g01406\_t002 |  | Atr-ERN16208 |  |  |  |  |  |  |  |  |  |  |
| 1 | Vvi-Vitvi08g01408\_t001 |  | \| \| |  |  |  |  |  |  |  |  |  |  |
| 1 | Vvi-Vitvi08g01409\_t001 |  | \| \| |  |  |  |  |  |  |  |  |  |  |
| 1 | Vvi-Vitvi08g01410\_t002 |  | \| \| |  |  |  |  |  |  |  |  |  |  |
| 2 | Vvi-Vitvi08g01411\_t001 |  | Atr-ERN16214 |  | Atr-ERM98949 |  |  |  |  |  |  |  |  |
| 1 | Vvi-Vitvi08g04300\_t001 |  |  |  | \| \| |  |  |  |  |  |  |  |  |
| 1 | Vvi-Vitvi08g01412\_t001 |  |  |  | Atr-ERM98946 |  |  |  |  |  |  |  |  |
| 1 | Vvi-Vitvi08g01413\_t001 |  |  |  | \| \| |  |  |  |  |  |  |  |  |
| 1 | Vvi-Vitvi08g04301\_t001 |  |  |  | \| \| |  |  |  |  |  |  |  |  |
| 1 | Vvi-Vitvi08g01414\_t001 |  |  |  | Atr-ERM98940 |  |  |  |  |  |  |  |  |
| 1 | Vvi-Vitvi08g01415\_t001 |  |  |  | Atr-ERM98939 |  |  |  |  |  |  |  |  |
| 1 | Vvi-Vitvi08g01416\_t001 |  |  |  | \| \| |  |  |  |  |  |  |  |  |
| 1 | Vvi-Vitvi08g02257\_t001 |  |  |  | Atr-ERM98938 |  |  |  |  |  |  |  |  |
| 1 | Vvi-Vitvi08g04302\_t001 |  |  |  | \| \| |  |  |  |  |  |  |  |  |
| 1 | Vvi-Vitvi08g01417\_t001 |  |  |  | Atr-ERM98931 |  |  |  |  |  |  |  |  |
| 1 | Vvi-Vitvi08g01418\_t001 |  | Atr-ERN13061 |  |  |  |  |  |  |  |  |  |  |
| 1 | Vvi-Vitvi08g01419\_t001 |  | Atr-ERN13060 |  |  |  |  |  |  |  |  |  |  |
| 1 | Vvi-Vitvi08g04303\_t001 |  | \| \| |  |  |  |  |  |  |  |  |  |  |
| 1 | Vvi-Vitvi08g01421\_t001 |  | Atr-ERN13057 |  |  |  |  |  |  |  |  |  |  |
| 1 | Vvi-Vitvi08g01422\_t001 |  | Atr-ERN13056 |  |  |  |  |  |  |  |  |  |  |
| 1 | Vvi-Vitvi08g01423\_t001 |  | Atr-ERN13055 |  |  |  |  |  |  |  |  |  |  |
| 1 | Vvi-Vitvi08g01425\_t001 |  | Atr-ERN13045 |  |  |  |  |  |  |  |  |  |  |
| 1 | Vvi-Vitvi08g01426\_t001 |  | Atr-ERN13043 |  |  |  |  |  |  |  |  |  |  |
| 1 | Vvi-Vitvi08g04304\_t001 |  | \| \| |  |  |  |  |  |  |  |  |  |  |
| 1 | Vvi-Vitvi08g01427\_t001 |  | \| \| |  |  |  |  |  |  |  |  |  |  |
| 1 | Vvi-Vitvi08g01428\_t001 |  | Atr-ERN13041 |  |  |  |  |  |  |  |  |  |  |
| 1 | Vvi-Vitvi08g02258\_t001 |  | Atr-ERN13040 |  |  |  |  |  |  |  |  |  |  |
| 1 | Vvi-Vitvi08g02259\_t001 |  | Atr-ERN13038 |  |  |  |  |  |  |  |  |  |  |
| 1 | Vvi-Vitvi08g02260\_t001 |  | \| \| |  |  |  |  |  |  |  |  |  |  |
| 1 | Vvi-Vitvi08g01429\_t001 |  | Atr-ERN13037 |  |  |  |  |  |  |  |  |  |  |
| 1 | Vvi-Vitvi08g01430\_t001 |  | \| \| |  |  |  |  |  |  |  |  |  |  |
| 1 | Vvi-Vitvi08g04305\_t001 |  | Atr-ERN13029 |  |  |  |  |  |  |  |  |  |  |
| 1 | Vvi-Vitvi08g01432\_t001 |  | Atr-ERN13028 |  |  |  |  |  |  |  |  |  |  |
| 1 | Vvi-Vitvi08g02261\_t001 |  | \| \| |  |  |  |  |  |  |  |  |  |  |
| 1 | Vvi-Vitvi08g01433\_t001 |  | \| \| |  |  |  |  |  |  |  |  |  |  |
| 1 | Vvi-Vitvi08g01434\_t001 |  | \| \| |  |  |  |  |  |  |  |  |  |  |
| 1 | Vvi-Vitvi08g02262\_t001 |  | \| \| |  |  |  |  |  |  |  |  |  |  |
| 1 | Vvi-Vitvi08g01436\_t001 |  | \| \| |  |  |  |  |  |  |  |  |  |  |
| 1 | Vvi-Vitvi08g01437\_t001 |  | Atr-ERN13020 |  |  |  |  |  |  |  |  |  |  |
| 1 | Vvi-Vitvi08g01438\_t001 |  | \| \| |  |  |  |  |  |  |  |  |  |  |
| 1 | Vvi-Vitvi08g01439\_t001 |  | Atr-ERN13013 |  |  |  |  |  |  |  |  |  |  |
| 1 | Vvi-Vitvi08g01441\_t001 |  | Atr-ERN13010 |  |  |  |  |  |  |  |  |  |  |
| 1 | Vvi-Vitvi08g01442\_t001 |  | Atr-ERN13009 |  |  |  |  |  |  |  |  |  |  |
| 0 | Vvi-Vitvi08g01443\_t001 |  |  |  |  |  |  |  |  |  |  |  |  |
| 1 | Vvi-Vitvi08g01444\_t001 |  | Atr-ERN16580 |  |  |  |  |  |  |  |  |  |  |
| 1 | Vvi-Vitvi08g01445\_t001 |  | \| \| |  |  |  |  |  |  |  |  |  |  |
| 1 | Vvi-Vitvi08g01448\_t001 |  | Atr-ERN16555 |  |  |  |  |  |  |  |  |  |  |
| 1 | Vvi-Vitvi08g04306\_t001 |  | \| \| |  |  |  |  |  |  |  |  |  |  |
| 1 | Vvi-Vitvi08g01450\_t002 |  | Atr-ERN16551 |  |  |  |  |  |  |  |  |  |  |
| 1 | Vvi-Vitvi08g04307\_t001 |  | \| \| |  |  |  |  |  |  |  |  |  |  |
| 1 | Vvi-Vitvi08g01451\_t001 |  | Atr-ERN16537 |  |  |  |  |  |  |  |  |  |  |
| 1 | Vvi-Vitvi08g01452\_t001 |  | \| \| |  |  |  |  |  |  |  |  |  |  |
| 1 | Vvi-Vitvi08g01454\_t001 |  | \| \| |  |  |  |  |  |  |  |  |  |  |
| 1 | Vvi-Vitvi08g01455\_t001 |  | Atr-ERN16532 |  |  |  |  |  |  |  |  |  |  |
| 1 | Vvi-Vitvi08g02264\_t001 |  | \| \| |  |  |  |  |  |  |  |  |  |  |
| 1 | Vvi-Vitvi08g01456\_t001 |  | Atr-ERN16512 |  |  |  |  |  |  |  |  |  |  |
| 1 | Vvi-Vitvi08g01457\_t001 |  | \| \| |  |  |  |  |  |  |  |  |  |  |
| 1 | Vvi-Vitvi08g01458\_t001 |  | \| \| |  |  |  |  |  |  |  |  |  |  |
| 1 | Vvi-Vitvi08g01459\_t001 |  | Atr-ERN16507 |  |  |  |  |  |  |  |  |  |  |
| 1 | Vvi-Vitvi08g02265\_t001 |  | Atr-ERN16491 |  |  |  |  |  |  |  |  |  |  |
| 1 | Vvi-Vitvi08g01460\_t002 |  | \| \| |  |  |  |  |  |  |  |  |  |  |
| 1 | Vvi-Vitvi08g01461\_t001 |  | \| \| |  |  |  |  |  |  |  |  |  |  |
| 1 | Vvi-Vitvi08g01462\_t001 |  | \| \| |  |  |  |  |  |  |  |  |  |  |
| 1 | Vvi-Vitvi08g01463\_t001 |  | \| \| |  |  |  |  |  |  |  |  |  |  |
| 1 | Vvi-Vitvi08g01464\_t001 |  | Atr-ERN16486 |  |  |  |  |  |  |  |  |  |  |
| 1 | Vvi-Vitvi08g01465\_t001 |  | \| \| |  |  |  |  |  |  |  |  |  |  |
| 1 | Vvi-Vitvi08g01466\_t001 |  | \| \| |  |  |  |  |  |  |  |  |  |  |
| 1 | Vvi-Vitvi08g01467\_t001 |  | Atr-ERN16485 |  |  |  |  |  |  |  |  |  |  |
| 1 | Vvi-Vitvi08g01468\_t001 |  | \| \| |  |  |  |  |  |  |  |  |  |  |
| 1 | Vvi-Vitvi08g04308\_t001 |  | \| \| |  |  |  |  |  |  |  |  |  |  |
| 1 | Vvi-Vitvi08g04309\_t001 |  | \| \| |  |  |  |  |  |  |  |  |  |  |
| 1 | Vvi-Vitvi08g01469\_t001 |  | Atr-ERN16484 |  |  |  |  |  |  |  |  |  |  |
| 0 | Vvi-Vitvi08g04310\_t001 |  |  |  |  |  |  |  |  |  |  |  |  |
| 0 | Vvi-Vitvi08g01470\_t001 |  |  |  |  |  |  |  |  |  |  |  |  |
| 0 | Vvi-Vitvi08g01471\_t001 |  |  |  |  |  |  |  |  |  |  |  |  |
| 0 | Vvi-Vitvi08g01472\_t001 |  |  |  |  |  |  |  |  |  |  |  |  |
| 0 | Vvi-Vitvi08g01473\_t001 |  |  |  |  |  |  |  |  |  |  |  |  |
| 0 | Vvi-Vitvi08g04311\_t001 |  |  |  |  |  |  |  |  |  |  |  |  |
| 0 | Vvi-Vitvi08g01474\_t001 |  |  |  |  |  |  |  |  |  |  |  |  |
| 0 | Vvi-Vitvi08g01475\_t001 |  |  |  |  |  |  |  |  |  |  |  |  |
| 0 | Vvi-Vitvi08g02266\_t001 |  |  |  |  |  |  |  |  |  |  |  |  |
| 0 | Vvi-Vitvi08g04312\_t001 |  |  |  |  |  |  |  |  |  |  |  |  |
| 0 | Vvi-Vitvi08g02267\_t001 |  |  |  |  |  |  |  |  |  |  |  |  |
| 0 | Vvi-Vitvi08g02268\_t001 |  |  |  |  |  |  |  |  |  |  |  |  |
| 0 | Vvi-Vitvi08g04313\_t001 |  |  |  |  |  |  |  |  |  |  |  |  |
| 0 | Vvi-Vitvi08g01476\_t001 |  |  |  |  |  |  |  |  |  |  |  |  |
| 0 | Vvi-Vitvi08g02270\_t001 |  |  |  |  |  |  |  |  |  |  |  |  |
| 0 | Vvi-Vitvi08g04314\_t001 |  |  |  |  |  |  |  |  |  |  |  |  |
| 0 | Vvi-Vitvi08g01478\_t001 |  |  |  |  |  |  |  |  |  |  |  |  |
| 0 | Vvi-Vitvi08g01480\_t001 |  |  |  |  |  |  |  |  |  |  |  |  |
| 0 | Vvi-Vitvi08g04315\_t001 |  |  |  |  |  |  |  |  |  |  |  |  |
| 0 | Vvi-Vitvi08g01481\_t001 |  |  |  |  |  |  |  |  |  |  |  |  |
| 1 | Vvi-Vitvi08g01482\_t001 |  | Atr-ERN13130 |  |  |  |  |  |  |  |  |  |  |
| 1 | Vvi-Vitvi08g01483\_t001 |  | Atr-ERN13127 |  |  |  |  |  |  |  |  |  |  |
| 1 | Vvi-Vitvi08g02271\_t001 |  | \| \| |  |  |  |  |  |  |  |  |  |  |
| 2 | Vvi-Vitvi08g01484\_t001 |  | \| \| |  | Atr-ERN13126 |  |  |  |  |  |  |  |  |
| 2 | Vvi-Vitvi08g02272\_t004 |  | Atr-ERN13115 |  | \| \| |  |  |  |  |  |  |  |  |
| 2 | Vvi-Vitvi08g01485\_t001 |  | \| \| |  | \| \| |  |  |  |  |  |  |  |  |
| 2 | Vvi-Vitvi08g04316\_t001 |  | \| \| |  | \| \| |  |  |  |  |  |  |  |  |
| 2 | Vvi-Vitvi08g01487\_t001 |  | Atr-ERN13098 |  | \| \| |  |  |  |  |  |  |  |  |
| 2 | Vvi-Vitvi08g04317\_t001 |  | \| \| |  | \| \| |  |  |  |  |  |  |  |  |
| 2 | Vvi-Vitvi08g01488\_t001 |  | Atr-ERN13089 |  | \| \| |  |  |  |  |  |  |  |  |
| 2 | Vvi-Vitvi08g01489\_t001 |  | Atr-ERN13088 |  | \| \| |  |  |  |  |  |  |  |  |
| 2 | Vvi-Vitvi08g01490\_t001 |  | Atr-ERN13083 |  | \| \| |  |  |  |  |  |  |  |  |
| 2 | Vvi-Vitvi08g01492\_t001 |  | Atr-ERN13081 |  | \| \| |  |  |  |  |  |  |  |  |
| 1 | Vvi-Vitvi08g01493\_t001 |  |  |  | Atr-ERN13136 |  |  |  |  |  |  |  |  |
| 1 | Vvi-Vitvi08g01494\_t001 |  |  |  | Atr-ERN13139 |  |  |  |  |  |  |  |  |
| 1 | Vvi-Vitvi08g04318\_t001 |  |  |  | \| \| |  |  |  |  |  |  |  |  |
| 1 | Vvi-Vitvi08g01497\_t001 |  |  |  | Atr-ERN13141 |  |  |  |  |  |  |  |  |
| 1 | Vvi-Vitvi08g01498\_t001 |  |  |  | Atr-ERN13142 |  |  |  |  |  |  |  |  |
| 1 | Vvi-Vitvi08g01499\_t001 |  |  |  | Atr-ERN13145 |  |  |  |  |  |  |  |  |
| 1 | Vvi-Vitvi08g04319\_t001 |  |  |  | \| \| |  |  |  |  |  |  |  |  |
| 1 | Vvi-Vitvi08g01500\_t001 |  |  |  | \| \| |  |  |  |  |  |  |  |  |
| 1 | Vvi-Vitvi08g01501\_t001 |  |  |  | Atr-ERN13151 |  |  |  |  |  |  |  |  |
| 1 | Vvi-Vitvi08g01502\_t001 |  |  |  | \| \| |  |  |  |  |  |  |  |  |
| 1 | Vvi-Vitvi08g04320\_t001 |  |  |  | \| \| |  |  |  |  |  |  |  |  |
| 1 | Vvi-Vitvi08g01505\_t001 |  |  |  | Atr-ERN13160 |  |  |  |  |  |  |  |  |
| 1 | Vvi-Vitvi08g01506\_t001 |  |  |  | Atr-ERN13161 |  |  |  |  |  |  |  |  |
| 1 | Vvi-Vitvi08g04321\_t001 |  |  |  | \| \| |  |  |  |  |  |  |  |  |
| 1 | Vvi-Vitvi08g01507\_t001 |  |  |  | Atr-ERN13162 |  |  |  |  |  |  |  |  |
| 1 | Vvi-Vitvi08g04322\_t001 |  |  |  | \| \| |  |  |  |  |  |  |  |  |
| 1 | Vvi-Vitvi08g04323\_t001 |  |  |  | \| \| |  |  |  |  |  |  |  |  |
| 1 | Vvi-Vitvi08g01508\_t002 |  |  |  | \| \| |  |  |  |  |  |  |  |  |
| 1 | Vvi-Vitvi08g01509\_t001 |  |  |  | Atr-ERN13164 |  |  |  |  |  |  |  |  |
| 1 | Vvi-Vitvi08g01510\_t001 |  |  |  | Atr-ERN13180 |  |  |  |  |  |  |  |  |
| 1 | Vvi-Vitvi08g01511\_t001 |  |  |  | \| \| |  |  |  |  |  |  |  |  |
| 1 | Vvi-Vitvi08g04324\_t001 |  |  |  | \| \| |  |  |  |  |  |  |  |  |
| 1 | Vvi-Vitvi08g01512\_t001 |  |  |  | \| \| |  |  |  |  |  |  |  |  |
| 1 | Vvi-Vitvi08g01513\_t001 |  |  |  | \| \| |  |  |  |  |  |  |  |  |
| 1 | Vvi-Vitvi08g04325\_t001 |  |  |  | \| \| |  |  |  |  |  |  |  |  |
| 1 | Vvi-Vitvi08g01514\_t001 |  |  |  | \| \| |  |  |  |  |  |  |  |  |
| 1 | Vvi-Vitvi08g01515\_t001 |  |  |  | Atr-ERN13187 |  |  |  |  |  |  |  |  |
| 1 | Vvi-Vitvi08g04326\_t001 |  |  |  | \| \| |  |  |  |  |  |  |  |  |
| 1 | Vvi-Vitvi08g04327\_t001 |  |  |  | \| \| |  |  |  |  |  |  |  |  |
| 1 | Vvi-Vitvi08g01517\_t001 |  |  |  | \| \| |  |  |  |  |  |  |  |  |
| 1 | Vvi-Vitvi08g01518\_t002 |  |  |  | \| \| |  |  |  |  |  |  |  |  |
| 1 | Vvi-Vitvi08g01519\_t003 |  |  |  | Atr-ERN13192 |  |  |  |  |  |  |  |  |
| 1 | Vvi-Vitvi08g01520\_t002 |  |  |  | \| \| |  |  |  |  |  |  |  |  |
| 1 | Vvi-Vitvi08g01521\_t001 |  |  |  | Atr-ERN13213 |  |  |  |  |  |  |  |  |
| 1 | Vvi-Vitvi08g01522\_t001 |  |  |  | \| \| |  |  |  |  |  |  |  |  |
| 1 | Vvi-Vitvi08g01523\_t002 |  |  |  | Atr-ERN13216 |  |  |  |  |  |  |  |  |
| 1 | Vvi-Vitvi08g02274\_t001 |  |  |  | \| \| |  |  |  |  |  |  |  |  |
| 1 | Vvi-Vitvi08g01524\_t001 |  |  |  | \| \| |  |  |  |  |  |  |  |  |
| 1 | Vvi-Vitvi08g01525\_t001 |  |  |  | Atr-ERN13221 |  |  |  |  |  |  |  |  |
| 1 | Vvi-Vitvi08g04328\_t001 |  |  |  | \| \| |  |  |  |  |  |  |  |  |
| 1 | Vvi-Vitvi08g01527\_t001 |  |  |  | Atr-ERN13225 |  |  |  |  |  |  |  |  |
| 1 | Vvi-Vitvi08g01528\_t001 |  |  |  | \| \| |  |  |  |  |  |  |  |  |
| 1 | Vvi-Vitvi08g02275\_t001 |  |  |  | \| \| |  |  |  |  |  |  |  |  |
| 1 | Vvi-Vitvi08g04329\_t001 |  |  |  | \| \| |  |  |  |  |  |  |  |  |
| 1 | Vvi-Vitvi08g01529\_t001 |  |  |  | \| \| |  |  |  |  |  |  |  |  |
| 1 | Vvi-Vitvi08g02277\_t001 |  |  |  | \| \| |  |  |  |  |  |  |  |  |
| 1 | Vvi-Vitvi08g04330\_t001 |  |  |  | \| \| |  |  |  |  |  |  |  |  |
| 1 | Vvi-Vitvi08g01531\_t001 |  |  |  | \| \| |  |  |  |  |  |  |  |  |
| 1 | Vvi-Vitvi08g04331\_t001 |  |  |  | \| \| |  |  |  |  |  |  |  |  |
| 1 | Vvi-Vitvi08g04332\_t001 |  |  |  | \| \| |  |  |  |  |  |  |  |  |
| 1 | Vvi-Vitvi08g04333\_t001 |  |  |  | \| \| |  |  |  |  |  |  |  |  |
| 1 | Vvi-Vitvi08g01534\_t001 |  |  |  | \| \| |  |  |  |  |  |  |  |  |
| 1 | Vvi-Vitvi08g04334\_t001 |  |  |  | \| \| |  |  |  |  |  |  |  |  |
| 1 | Vvi-Vitvi08g02280\_t001 |  |  |  | \| \| |  |  |  |  |  |  |  |  |
| 1 | Vvi-Vitvi08g01535\_t001 |  |  |  | \| \| |  |  |  |  |  |  |  |  |
| 1 | Vvi-Vitvi08g01536\_t001 |  |  |  | Atr-ERN13227 |  |  |  |  |  |  |  |  |
| 1 | Vvi-Vitvi08g02281\_t001 |  |  |  | Atr-ERN13233 |  |  |  |  |  |  |  |  |
| 1 | Vvi-Vitvi08g01537\_t001 |  |  |  | Atr-ERN13235 |  |  |  |  |  |  |  |  |
| 1 | Vvi-Vitvi08g01538\_t002 |  |  |  | \| \| |  |  |  |  |  |  |  |  |
| 1 | Vvi-Vitvi08g01539\_t001 |  |  |  | Atr-ERN13242 |  |  |  |  |  |  |  |  |
| 1 | Vvi-Vitvi08g01540\_t001 |  |  |  | Atr-ERN13247 |  |  |  |  |  |  |  |  |
| 1 | Vvi-Vitvi08g04335\_t002 |  |  |  | \| \| |  |  |  |  |  |  |  |  |
| 1 | Vvi-Vitvi08g01542\_t001 |  |  |  | Atr-ERN13259 |  |  |  |  |  |  |  |  |
| 1 | Vvi-Vitvi08g04336\_t001 |  |  |  | \| \| |  |  |  |  |  |  |  |  |
| 1 | Vvi-Vitvi08g01543\_t001 |  |  |  | Atr-ERN13260 |  |  |  |  |  |  |  |  |
| 0 | Vvi-Vitvi08g01544\_t001 |  |  |  |  |  |  |  |  |  |  |  |  |
| 1 | Vvi-Vitvi08g01545\_t001 |  | Atr-ERN01300 |  |  |  |  |  |  |  |  |  |  |
| 1 | Vvi-Vitvi08g01546\_t001 |  | Atr-ERN01308 |  |  |  |  |  |  |  |  |  |  |
| 1 | Vvi-Vitvi08g04337\_t001 |  | \| \| |  |  |  |  |  |  |  |  |  |  |
| 1 | Vvi-Vitvi08g01547\_t001 |  | Atr-ERN01312 |  |  |  |  |  |  |  |  |  |  |
| 1 | Vvi-Vitvi08g01548\_t001 |  | \| \| |  |  |  |  |  |  |  |  |  |  |
| 1 | Vvi-Vitvi08g01549\_t001 |  | \| \| |  |  |  |  |  |  |  |  |  |  |
| 1 | Vvi-Vitvi08g01550\_t001 |  | \| \| |  |  |  |  |  |  |  |  |  |  |
| 1 | Vvi-Vitvi08g01551\_t001.1.6037826f |  | Atr-ERN01320 |  |  |  |  |  |  |  |  |  |  |
| 1 | Vvi-Vitvi08g01553\_t001 |  | Atr-ERN01321 |  |  |  |  |  |  |  |  |  |  |
| 1 | Vvi-Vitvi08g01554\_t001 |  | \| \| |  |  |  |  |  |  |  |  |  |  |
| 1 | Vvi-Vitvi08g02282\_t001 |  | \| \| |  |  |  |  |  |  |  |  |  |  |
| 1 | Vvi-Vitvi08g01555\_t001 |  | Atr-ERN01323 |  |  |  |  |  |  |  |  |  |  |
| 1 | Vvi-Vitvi08g01556\_t001 |  | Atr-ERN01332 |  |  |  |  |  |  |  |  |  |  |
| 1 | Vvi-Vitvi08g01558\_t001 |  | Atr-ERN01343 |  |  |  |  |  |  |  |  |  |  |
| 1 | Vvi-Vitvi08g01559\_t001 |  | \| \| |  |  |  |  |  |  |  |  |  |  |
| 1 | Vvi-Vitvi08g01560\_t001 |  | \| \| |  |  |  |  |  |  |  |  |  |  |
| 1 | Vvi-Vitvi08g01561\_t001 |  | Atr-ERN01344 |  |  |  |  |  |  |  |  |  |  |
| 1 | Vvi-Vitvi08g01562\_t001 |  | Atr-ERN01347 |  |  |  |  |  |  |  |  |  |  |
| 0 | Vvi-Vitvi08g04338\_t001 |  |  |  |  |  |  |  |  |  |  |  |  |
| 0 | Vvi-Vitvi08g04339\_t001 |  |  |  |  |  |  |  |  |  |  |  |  |
| 0 | Vvi-Vitvi08g02283\_t002 |  |  |  |  |  |  |  |  |  |  |  |  |
| 0 | Vvi-Vitvi08g02286\_t001 |  |  |  |  |  |  |  |  |  |  |  |  |
| 0 | Vvi-Vitvi08g04340\_t001 |  |  |  |  |  |  |  |  |  |  |  |  |
| 0 | Vvi-Vitvi08g01567\_t001 |  |  |  |  |  |  |  |  |  |  |  |  |
| 0 | Vvi-Vitvi08g01568\_t001 |  |  |  |  |  |  |  |  |  |  |  |  |
| 0 | Vvi-Vitvi08g01569\_t001 |  |  |  |  |  |  |  |  |  |  |  |  |
| 0 | Vvi-Vitvi08g01570\_t001 |  |  |  |  |  |  |  |  |  |  |  |  |
| 0 | Vvi-Vitvi08g01571\_t001 |  |  |  |  |  |  |  |  |  |  |  |  |
| 0 | Vvi-Vitvi08g01572\_t001 |  |  |  |  |  |  |  |  |  |  |  |  |
| 0 | Vvi-Vitvi08g01573\_t001 |  |  |  |  |  |  |  |  |  |  |  |  |
| 0 | Vvi-Vitvi08g01574\_t001 |  |  |  |  |  |  |  |  |  |  |  |  |
| 0 | Vvi-Vitvi08g04341\_t001 |  |  |  |  |  |  |  |  |  |  |  |  |
| 0 | Vvi-Vitvi08g01575\_t001 |  |  |  |  |  |  |  |  |  |  |  |  |
| 0 | Vvi-Vitvi08g01577\_t001 |  |  |  |  |  |  |  |  |  |  |  |  |
| 0 | Vvi-Vitvi08g01578\_t001 |  |  |  |  |  |  |  |  |  |  |  |  |
| 0 | Vvi-Vitvi08g01579\_t002 |  |  |  |  |  |  |  |  |  |  |  |  |
| 0 | Vvi-Vitvi08g01580\_t001 |  |  |  |  |  |  |  |  |  |  |  |  |
| 0 | Vvi-Vitvi08g02288\_t001 |  |  |  |  |  |  |  |  |  |  |  |  |
| 0 | Vvi-Vitvi08g04342\_t001 |  |  |  |  |  |  |  |  |  |  |  |  |
| 0 | Vvi-Vitvi08g01581\_t001 |  |  |  |  |  |  |  |  |  |  |  |  |
| 0 | Vvi-Vitvi08g02289\_t001 |  |  |  |  |  |  |  |  |  |  |  |  |
| 0 | Vvi-Vitvi08g01582\_t001 |  |  |  |  |  |  |  |  |  |  |  |  |
| 0 | Vvi-Vitvi08g01583\_t001 |  |  |  |  |  |  |  |  |  |  |  |  |
| 0 | Vvi-Vitvi08g02291\_t001 |  |  |  |  |  |  |  |  |  |  |  |  |
| 0 | Vvi-Vitvi08g04343\_t001 |  |  |  |  |  |  |  |  |  |  |  |  |
| 0 | Vvi-Vitvi08g01584\_t001 |  |  |  |  |  |  |  |  |  |  |  |  |
| 0 | Vvi-Vitvi08g01585\_t001 |  |  |  |  |  |  |  |  |  |  |  |  |
| 0 | Vvi-Vitvi08g02292\_t001 |  |  |  |  |  |  |  |  |  |  |  |  |
| 0 | Vvi-Vitvi08g01586\_t001 |  |  |  |  |  |  |  |  |  |  |  |  |
| 0 | Vvi-Vitvi08g01587\_t001 |  |  |  |  |  |  |  |  |  |  |  |  |
| 0 | Vvi-Vitvi08g01588\_t001 |  |  |  |  |  |  |  |  |  |  |  |  |
| 0 | Vvi-Vitvi08g04344\_t001 |  |  |  |  |  |  |  |  |  |  |  |  |
| 0 | Vvi-Vitvi08g01589\_t001 |  |  |  |  |  |  |  |  |  |  |  |  |
| 0 | Vvi-Vitvi08g01591\_t001 |  |  |  |  |  |  |  |  |  |  |  |  |
| 0 | Vvi-Vitvi08g04345\_t001 |  |  |  |  |  |  |  |  |  |  |  |  |
| 0 | Vvi-Vitvi08g01596\_t001 |  |  |  |  |  |  |  |  |  |  |  |  |
| 0 | Vvi-Vitvi08g01597\_t001 |  |  |  |  |  |  |  |  |  |  |  |  |
| 0 | Vvi-Vitvi08g01599\_t001 |  |  |  |  |  |  |  |  |  |  |  |  |
| 0 | Vvi-Vitvi08g01600\_t001 |  |  |  |  |  |  |  |  |  |  |  |  |
| 0 | Vvi-Vitvi08g01602\_t001 |  |  |  |  |  |  |  |  |  |  |  |  |
| 0 | Vvi-Vitvi08g01603\_t004 |  |  |  |  |  |  |  |  |  |  |  |  |
| 0 | Vvi-Vitvi08g01604\_t001 |  |  |  |  |  |  |  |  |  |  |  |  |
| 0 | Vvi-Vitvi08g02295\_t001 |  |  |  |  |  |  |  |  |  |  |  |  |
| 0 | Vvi-Vitvi08g01606\_t001 |  |  |  |  |  |  |  |  |  |  |  |  |
| 0 | Vvi-Vitvi08g01607\_t001 |  |  |  |  |  |  |  |  |  |  |  |  |
| 0 | Vvi-Vitvi08g01608\_t001 |  |  |  |  |  |  |  |  |  |  |  |  |
| 0 | Vvi-Vitvi08g01609\_t001 |  |  |  |  |  |  |  |  |  |  |  |  |
| 0 | Vvi-Vitvi08g01611\_t001 |  |  |  |  |  |  |  |  |  |  |  |  |
| 0 | Vvi-Vitvi08g01612\_t001 |  |  |  |  |  |  |  |  |  |  |  |  |
| 0 | Vvi-Vitvi08g01613\_t001 |  |  |  |  |  |  |  |  |  |  |  |  |
| 0 | Vvi-Vitvi08g01614\_t001 |  |  |  |  |  |  |  |  |  |  |  |  |
| 0 | Vvi-Vitvi08g02296\_t002 |  |  |  |  |  |  |  |  |  |  |  |  |
| 0 | Vvi-Vitvi08g01615\_t001 |  |  |  |  |  |  |  |  |  |  |  |  |
| 0 | Vvi-Vitvi08g01616\_t001 |  |  |  |  |  |  |  |  |  |  |  |  |
| 0 | Vvi-Vitvi08g04346\_t001 |  |  |  |  |  |  |  |  |  |  |  |  |
| 0 | Vvi-Vitvi08g04347\_t001 |  |  |  |  |  |  |  |  |  |  |  |  |
| 0 | Vvi-Vitvi08g01617\_t001 |  |  |  |  |  |  |  |  |  |  |  |  |
| 0 | Vvi-Vitvi08g04348\_t001 |  |  |  |  |  |  |  |  |  |  |  |  |
| 0 | Vvi-Vitvi08g02299\_t001 |  |  |  |  |  |  |  |  |  |  |  |  |
| 0 | Vvi-Vitvi08g01618\_t001 |  |  |  |  |  |  |  |  |  |  |  |  |
| 0 | Vvi-Vitvi08g01619\_t001 |  |  |  |  |  |  |  |  |  |  |  |  |
| 0 | Vvi-Vitvi08g04349\_t001 |  |  |  |  |  |  |  |  |  |  |  |  |
| 0 | Vvi-Vitvi08g01621\_t001 |  |  |  |  |  |  |  |  |  |  |  |  |
| 0 | Vvi-Vitvi08g01622\_t001 |  |  |  |  |  |  |  |  |  |  |  |  |
| 0 | Vvi-Vitvi08g01623\_t001 |  |  |  |  |  |  |  |  |  |  |  |  |
| 0 | Vvi-Vitvi08g01624\_t001 |  |  |  |  |  |  |  |  |  |  |  |  |
| 0 | Vvi-Vitvi08g04350\_t001 |  |  |  |  |  |  |  |  |  |  |  |  |
| 0 | Vvi-Vitvi08g04351\_t001 |  |  |  |  |  |  |  |  |  |  |  |  |
| 0 | Vvi-Vitvi08g01627\_t001 |  |  |  |  |  |  |  |  |  |  |  |  |
| 0 | Vvi-Vitvi08g04352\_t001 |  |  |  |  |  |  |  |  |  |  |  |  |
| 0 | Vvi-Vitvi08g01629\_t002 |  |  |  |  |  |  |  |  |  |  |  |  |
| 0 | Vvi-Vitvi08g01630\_t001 |  |  |  |  |  |  |  |  |  |  |  |  |
| 0 | Vvi-Vitvi08g01631\_t001 |  |  |  |  |  |  |  |  |  |  |  |  |
| 0 | Vvi-Vitvi08g02301\_t001 |  |  |  |  |  |  |  |  |  |  |  |  |
| 0 | Vvi-Vitvi08g01634\_t001 |  |  |  |  |  |  |  |  |  |  |  |  |
| 0 | Vvi-Vitvi08g01635\_t001 |  |  |  |  |  |  |  |  |  |  |  |  |
| 0 | Vvi-Vitvi08g04353\_t001 |  |  |  |  |  |  |  |  |  |  |  |  |
| 0 | Vvi-Vitvi08g04354\_t001 |  |  |  |  |  |  |  |  |  |  |  |  |
| 0 | Vvi-Vitvi08g04355\_t001 |  |  |  |  |  |  |  |  |  |  |  |  |
| 0 | Vvi-Vitvi08g01638\_t001 |  |  |  |  |  |  |  |  |  |  |  |  |
| 0 | Vvi-Vitvi08g01639\_t001 |  |  |  |  |  |  |  |  |  |  |  |  |
| 0 | Vvi-Vitvi08g01640\_t001 |  |  |  |  |  |  |  |  |  |  |  |  |
| 0 | Vvi-Vitvi08g01641\_t001 |  |  |  |  |  |  |  |  |  |  |  |  |
| 0 | Vvi-Vitvi08g02304\_t001 |  |  |  |  |  |  |  |  |  |  |  |  |
| 0 | Vvi-Vitvi08g01642\_t001 |  |  |  |  |  |  |  |  |  |  |  |  |
| 0 | Vvi-Vitvi08g01644\_t001 |  |  |  |  |  |  |  |  |  |  |  |  |
| 0 | Vvi-Vitvi08g04356\_t001 |  |  |  |  |  |  |  |  |  |  |  |  |
| 0 | Vvi-Vitvi08g01645\_t001 |  |  |  |  |  |  |  |  |  |  |  |  |
| 0 | Vvi-Vitvi08g01646\_t001 |  |  |  |  |  |  |  |  |  |  |  |  |
| 0 | Vvi-Vitvi08g04357\_t001 |  |  |  |  |  |  |  |  |  |  |  |  |
| 0 | Vvi-Vitvi08g04358\_t001 |  |  |  |  |  |  |  |  |  |  |  |  |
| 1 | Vvi-Vitvi08g01649\_t001 |  | Atr-ERN16263 |  |  |  |  |  |  |  |  |  |  |
| 1 | Vvi-Vitvi08g01651\_t001 |  | \| \| |  |  |  |  |  |  |  |  |  |  |
| 1 | Vvi-Vitvi08g01652\_t001 |  | \| \| |  |  |  |  |  |  |  |  |  |  |
| 1 | Vvi-Vitvi08g01653\_t001 |  | \| \| |  |  |  |  |  |  |  |  |  |  |
| 1 | Vvi-Vitvi08g01655\_t002 |  | Atr-ERN16255 |  |  |  |  |  |  |  |  |  |  |
| 1 | Vvi-Vitvi08g01656\_t001 |  | \| \| |  |  |  |  |  |  |  |  |  |  |
| 1 | Vvi-Vitvi08g01657\_t001 |  | \| \| |  |  |  |  |  |  |  |  |  |  |
| 1 | Vvi-Vitvi08g02307\_t001 |  | Atr-ERN16243 |  |  |  |  |  |  |  |  |  |  |
| 1 | Vvi-Vitvi08g04359\_t001 |  | \| \| |  |  |  |  |  |  |  |  |  |  |
| 1 | Vvi-Vitvi08g01659\_t001 |  | Atr-ERN16234 |  |  |  |  |  |  |  |  |  |  |
| 1 | Vvi-Vitvi08g01660\_t001 |  | \| \| |  |  |  |  |  |  |  |  |  |  |
| 1 | Vvi-Vitvi08g01661\_t002 |  | \| \| |  |  |  |  |  |  |  |  |  |  |
| 1 | Vvi-Vitvi08g01662\_t001 |  | \| \| |  |  |  |  |  |  |  |  |  |  |
| 1 | Vvi-Vitvi08g02308\_t001 |  | \| \| |  |  |  |  |  |  |  |  |  |  |
| 1 | Vvi-Vitvi08g02309\_t001 |  | \| \| |  |  |  |  |  |  |  |  |  |  |
| 1 | Vvi-Vitvi08g04360\_t001 |  | \| \| |  |  |  |  |  |  |  |  |  |  |
| 1 | Vvi-Vitvi08g04361\_t001 |  | \| \| |  |  |  |  |  |  |  |  |  |  |
| 1 | Vvi-Vitvi08g04362\_t001 |  | \| \| |  |  |  |  |  |  |  |  |  |  |
| 1 | Vvi-Vitvi08g01664\_t001 |  | \| \| |  |  |  |  |  |  |  |  |  |  |
| 1 | Vvi-Vitvi08g04363\_t001 |  | \| \| |  |  |  |  |  |  |  |  |  |  |
| 1 | Vvi-Vitvi08g01666\_t001 |  | Atr-ERN16221 |  |  |  |  |  |  |  |  |  |  |
| 1 | Vvi-Vitvi08g04364\_t001 |  | \| \| |  |  |  |  |  |  |  |  |  |  |
| 1 | Vvi-Vitvi08g01667\_t001 |  | \| \| |  |  |  |  |  |  |  |  |  |  |
| 1 | Vvi-Vitvi08g01668\_t001 |  | Atr-ERN16220 |  |  |  |  |  |  |  |  |  |  |
| 0 | Vvi-Vitvi08g02313\_t001 |  |  |  |  |  |  |  |  |  |  |  |  |
| 0 | Vvi-Vitvi08g02314\_t001 |  |  |  |  |  |  |  |  |  |  |  |  |
| 0 | Vvi-Vitvi08g02315\_t001 |  |  |  |  |  |  |  |  |  |  |  |  |
| 0 | Vvi-Vitvi08g01669\_t001 |  |  |  |  |  |  |  |  |  |  |  |  |
| 0 | Vvi-Vitvi08g02317\_t001 |  |  |  |  |  |  |  |  |  |  |  |  |
| 0 | Vvi-Vitvi08g01670\_t001 |  |  |  |  |  |  |  |  |  |  |  |  |
| 0 | Vvi-Vitvi08g01671\_t001 |  |  |  |  |  |  |  |  |  |  |  |  |
| 0 | Vvi-Vitvi08g04365\_t001 |  |  |  |  |  |  |  |  |  |  |  |  |
| 0 | Vvi-Vitvi08g01672\_t001 |  |  |  |  |  |  |  |  |  |  |  |  |
| 0 | Vvi-Vitvi08g01673\_t001 |  |  |  |  |  |  |  |  |  |  |  |  |
| 0 | Vvi-Vitvi08g04366\_t001 |  |  |  |  |  |  |  |  |  |  |  |  |
| 0 | Vvi-Vitvi08g04367\_t001 |  |  |  |  |  |  |  |  |  |  |  |  |
| 0 | Vvi-Vitvi08g04368\_t001 |  |  |  |  |  |  |  |  |  |  |  |  |
| 0 | Vvi-Vitvi08g04369\_t001 |  |  |  |  |  |  |  |  |  |  |  |  |
| 0 | Vvi-Vitvi08g02318\_t001 |  |  |  |  |  |  |  |  |  |  |  |  |
| 0 | Vvi-Vitvi08g04370\_t001 |  |  |  |  |  |  |  |  |  |  |  |  |
| 0 | Vvi-Vitvi08g04371\_t001 |  |  |  |  |  |  |  |  |  |  |  |  |
| 0 | Vvi-Vitvi08g02320\_t003 |  |  |  |  |  |  |  |  |  |  |  |  |
| 0 | Vvi-Vitvi08g04372\_t001 |  |  |  |  |  |  |  |  |  |  |  |  |
| 0 | Vvi-Vitvi08g04373\_t001 |  |  |  |  |  |  |  |  |  |  |  |  |
| 0 | Vvi-Vitvi08g04374\_t001 |  |  |  |  |  |  |  |  |  |  |  |  |
| 0 | Vvi-Vitvi08g04375\_t001 |  |  |  |  |  |  |  |  |  |  |  |  |
| 0 | Vvi-Vitvi08g02323\_t001 |  |  |  |  |  |  |  |  |  |  |  |  |
| 0 | Vvi-Vitvi08g04376\_t001 |  |  |  |  |  |  |  |  |  |  |  |  |
| 0 | Vvi-Vitvi08g04377\_t001 |  |  |  |  |  |  |  |  |  |  |  |  |
| 0 | Vvi-Vitvi08g02324\_t001 |  |  |  |  |  |  |  |  |  |  |  |  |
| 0 | Vvi-Vitvi08g04378\_t001 |  |  |  |  |  |  |  |  |  |  |  |  |
| 0 | Vvi-Vitvi08g02326\_t001 |  |  |  |  |  |  |  |  |  |  |  |  |
| 0 | Vvi-Vitvi08g01674\_t001 |  |  |  |  |  |  |  |  |  |  |  |  |
| 0 | Vvi-Vitvi08g04379\_t001 |  |  |  |  |  |  |  |  |  |  |  |  |
| 0 | Vvi-Vitvi08g01675\_t002 |  |  |  |  |  |  |  |  |  |  |  |  |
| 0 | Vvi-Vitvi08g01676\_t001 |  |  |  |  |  |  |  |  |  |  |  |  |
| 0 | Vvi-Vitvi08g04380\_t001 |  |  |  |  |  |  |  |  |  |  |  |  |
| 0 | Vvi-Vitvi08g01677\_t001 |  |  |  |  |  |  |  |  |  |  |  |  |
| 1 | Vvi-Vitvi08g01678\_t001 |  | Atr-ERN05068 |  |  |  |  |  |  |  |  |  |  |
| 1 | Vvi-Vitvi08g01679\_t001 |  | \| \| |  |  |  |  |  |  |  |  |  |  |
| 1 | Vvi-Vitvi08g01680\_t001 |  | \| \| |  |  |  |  |  |  |  |  |  |  |
| 1 | Vvi-Vitvi08g01681\_t001 |  | \| \| |  |  |  |  |  |  |  |  |  |  |
| 1 | Vvi-Vitvi08g01682\_t001 |  | \| \| |  |  |  |  |  |  |  |  |  |  |
| 1 | Vvi-Vitvi08g01683\_t001 |  | Atr-ERN05072 |  |  |  |  |  |  |  |  |  |  |
| 1 | Vvi-Vitvi08g01684\_t001 |  | \| \| |  |  |  |  |  |  |  |  |  |  |
| 1 | Vvi-Vitvi08g01685\_t001 |  | Atr-ERN05078 |  |  |  |  |  |  |  |  |  |  |
| 1 | Vvi-Vitvi08g02328\_t001 |  | \| \| |  |  |  |  |  |  |  |  |  |  |
| 1 | Vvi-Vitvi08g04381\_t001 |  | \| \| |  |  |  |  |  |  |  |  |  |  |
| 1 | Vvi-Vitvi08g02329\_t001 |  | \| \| |  |  |  |  |  |  |  |  |  |  |
| 1 | Vvi-Vitvi08g04382\_t001 |  | \| \| |  |  |  |  |  |  |  |  |  |  |
| 1 | Vvi-Vitvi08g01686\_t001 |  | Atr-ERN05084 |  |  |  |  |  |  |  |  |  |  |
| 1 | Vvi-Vitvi08g01687\_t001 |  | \| \| |  |  |  |  |  |  |  |  |  |  |
| 1 | Vvi-Vitvi08g04383\_t001 |  | \| \| |  |  |  |  |  |  |  |  |  |  |
| 1 | Vvi-Vitvi08g01689\_t001 |  | \| \| |  |  |  |  |  |  |  |  |  |  |
| 1 | Vvi-Vitvi08g01690\_t001 |  | \| \| |  |  |  |  |  |  |  |  |  |  |
| 1 | Vvi-Vitvi08g01691\_t001 |  | Atr-ERN05104 |  |  |  |  |  |  |  |  |  |  |
| 1 | Vvi-Vitvi08g01692\_t001 |  | Atr-ERN05106 |  |  |  |  |  |  |  |  |  |  |
| 1 | Vvi-Vitvi08g01693\_t001 |  | Atr-ERN05110 |  |  |  |  |  |  |  |  |  |  |
| 1 | Vvi-Vitvi08g01694\_t001 |  | \| \| |  |  |  |  |  |  |  |  |  |  |
| 1 | Vvi-Vitvi08g01968\_t001 |  | \| \| |  |  |  |  |  |  |  |  |  |  |
| 1 | Vvi-Vitvi08g01695\_t001 |  | \| \| |  |  |  |  |  |  |  |  |  |  |
| 1 | Vvi-Vitvi08g01696\_t001 |  | \| \| |  |  |  |  |  |  |  |  |  |  |
| 1 | Vvi-Vitvi08g01697\_t001 |  | Atr-ERN05126 |  |  |  |  |  |  |  |  |  |  |
| 1 | Vvi-Vitvi08g01698\_t001 |  | Atr-ERN05128 |  |  |  |  |  |  |  |  |  |  |
| 1 | Vvi-Vitvi08g02330\_t001 |  | Atr-ERN05130 |  |  |  |  |  |  |  |  |  |  |
| 1 | Vvi-Vitvi08g02331\_t001 |  | \| \| |  |  |  |  |  |  |  |  |  |  |
| 1 | Vvi-Vitvi08g02332\_t002 |  | \| \| |  |  |  |  |  |  |  |  |  |  |
| 1 | Vvi-Vitvi08g01699\_t001 |  | Atr-ERN05132 |  |  |  |  |  |  |  |  |  |  |
| 1 | Vvi-Vitvi08g02333\_t001 |  | \| \| |  |  |  |  |  |  |  |  |  |  |
| 1 | Vvi-Vitvi08g02334\_t001 |  | Atr-ERN05134 |  |  |  |  |  |  |  |  |  |  |
| 0 | Vvi-Vitvi08g02335\_t001 |  |  |  |  |  |  |  |  |  |  |  |  |
| 0 | Vvi-Vitvi08g02336\_t001 |  |  |  |  |  |  |  |  |  |  |  |  |
| 0 | Vvi-Vitvi08g01701\_t001 |  |  |  |  |  |  |  |  |  |  |  |  |
| 0 | Vvi-Vitvi08g04384\_t001 |  |  |  |  |  |  |  |  |  |  |  |  |
| 0 | Vvi-Vitvi08g01702\_t001 |  |  |  |  |  |  |  |  |  |  |  |  |
| 0 | Vvi-Vitvi08g04385\_t001 |  |  |  |  |  |  |  |  |  |  |  |  |
| 0 | Vvi-Vitvi08g04386\_t001 |  |  |  |  |  |  |  |  |  |  |  |  |
| 0 | Vvi-Vitvi08g01707\_t001 |  |  |  |  |  |  |  |  |  |  |  |  |
| 0 | Vvi-Vitvi08g04387\_t001 |  |  |  |  |  |  |  |  |  |  |  |  |
| 1 | Vvi-Vitvi08g01708\_t001 |  | Atr-ERN11666 |  |  |  |  |  |  |  |  |  |  |
| 1 | Vvi-Vitvi08g01709\_t001 |  | Atr-ERN11664 |  |  |  |  |  |  |  |  |  |  |
| 1 | Vvi-Vitvi08g01710\_t001 |  | Atr-ERN11662 |  |  |  |  |  |  |  |  |  |  |
| 1 | Vvi-Vitvi08g01711\_t001 |  | \| \| |  |  |  |  |  |  |  |  |  |  |
| 1 | Vvi-Vitvi08g04388\_t001 |  | \| \| |  |  |  |  |  |  |  |  |  |  |
| 1 | Vvi-Vitvi08g04389\_t001 |  | \| \| |  |  |  |  |  |  |  |  |  |  |
| 1 | Vvi-Vitvi08g01713\_t001 |  | Atr-ERN11654 |  |  |  |  |  |  |  |  |  |  |
| 1 | Vvi-Vitvi08g01714\_t001 |  | \| \| |  |  |  |  |  |  |  |  |  |  |
| 1 | Vvi-Vitvi08g04390\_t001 |  | \| \| |  |  |  |  |  |  |  |  |  |  |
| 1 | Vvi-Vitvi08g01716\_t002 |  | Atr-ERN11649 |  |  |  |  |  |  |  |  |  |  |
| 1 | Vvi-Vitvi08g01717\_t001 |  | Atr-ERN11638 |  |  |  |  |  |  |  |  |  |  |
| 1 | Vvi-Vitvi08g01718\_t001 |  | Atr-ERN11631 |  |  |  |  |  |  |  |  |  |  |
| 1 | Vvi-Vitvi08g01719\_t001 |  | Atr-ERN11630 |  |  |  |  |  |  |  |  |  |  |
| 1 | Vvi-Vitvi08g01720\_t001 |  | Atr-ERN11629 |  |  |  |  |  |  |  |  |  |  |
| 1 | Vvi-Vitvi08g02342\_t001 |  | Atr-ERN11627 |  |  |  |  |  |  |  |  |  |  |
| 1 | Vvi-Vitvi08g04391\_t001 |  | \| \| |  |  |  |  |  |  |  |  |  |  |
| 1 | Vvi-Vitvi08g01721\_t001 |  | \| \| |  |  |  |  |  |  |  |  |  |  |
| 1 | Vvi-Vitvi08g01722\_t001 |  | \| \| |  |  |  |  |  |  |  |  |  |  |
| 1 | Vvi-Vitvi08g01723\_t002 |  | Atr-ERN11610 |  |  |  |  |  |  |  |  |  |  |
| 1 | Vvi-Vitvi08g01724\_t001 |  | Atr-ERN11602 |  |  |  |  |  |  |  |  |  |  |
| 1 | Vvi-Vitvi08g04392\_t001 |  | \| \| |  |  |  |  |  |  |  |  |  |  |
| 1 | Vvi-Vitvi08g01725\_t001 |  | Atr-ERN11599 |  |  |  |  |  |  |  |  |  |  |
| 1 | Vvi-Vitvi08g04393\_t001 |  | \| \| |  |  |  |  |  |  |  |  |  |  |
| 1 | Vvi-Vitvi08g01728\_t001 |  | Atr-ERN11594 |  |  |  |  |  |  |  |  |  |  |
| 1 | Vvi-Vitvi08g04394\_t001 |  | \| \| |  |  |  |  |  |  |  |  |  |  |
| 1 | Vvi-Vitvi08g01729\_t001 |  | \| \| |  |  |  |  |  |  |  |  |  |  |
| 1 | Vvi-Vitvi08g01730\_t001 |  | \| \| |  |  |  |  |  |  |  |  |  |  |
| 1 | Vvi-Vitvi08g01731\_t001 |  | Atr-ERN11591 |  |  |  |  |  |  |  |  |  |  |
| 1 | Vvi-Vitvi08g01732\_t001 |  | Atr-ERN11588 |  |  |  |  |  |  |  |  |  |  |
| 1 | Vvi-Vitvi08g04395\_t001 |  | \| \| |  |  |  |  |  |  |  |  |  |  |
| 1 | Vvi-Vitvi08g01733\_t001 |  | \| \| |  |  |  |  |  |  |  |  |  |  |
| 1 | Vvi-Vitvi08g02343\_t001 |  | Atr-ERN11583 |  |  |  |  |  |  |  |  |  |  |
| 1 | Vvi-Vitvi08g02344\_t001 |  | \| \| |  |  |  |  |  |  |  |  |  |  |
| 1 | Vvi-Vitvi08g01734\_t001 |  | \| \| |  |  |  |  |  |  |  |  |  |  |
| 1 | Vvi-Vitvi08g04396\_t001 |  | \| \| |  |  |  |  |  |  |  |  |  |  |
| 1 | Vvi-Vitvi08g02345\_t001 |  | Atr-ERN11582 |  |  |  |  |  |  |  |  |  |  |
| 1 | Vvi-Vitvi08g01735\_t001 |  | \| \| |  |  |  |  |  |  |  |  |  |  |
| 1 | Vvi-Vitvi08g01736\_t001 |  | \| \| |  |  |  |  |  |  |  |  |  |  |
| 1 | Vvi-Vitvi08g02346\_t001 |  | Atr-ERN11576 |  |  |  |  |  |  |  |  |  |  |
| 1 | Vvi-Vitvi08g01737\_t002 |  | Atr-ERN17104 |  |  |  |  |  |  |  |  |  |  |
| 1 | Vvi-Vitvi08g01738\_t001 |  | Atr-ERN17101 |  |  |  |  |  |  |  |  |  |  |
| 1 | Vvi-Vitvi08g01739\_t002 |  | Atr-ERN17098 |  |  |  |  |  |  |  |  |  |  |
| 1 | Vvi-Vitvi08g01740\_t001 |  | Atr-ERN17092 |  |  |  |  |  |  |  |  |  |  |
| 1 | Vvi-Vitvi08g01741\_t001 |  | Atr-ERN17089 |  |  |  |  |  |  |  |  |  |  |
| 1 | Vvi-Vitvi08g01742\_t001 |  | Atr-ERN17085 |  |  |  |  |  |  |  |  |  |  |
| 1 | Vvi-Vitvi08g01743\_t001 |  | \| \| |  |  |  |  |  |  |  |  |  |  |
| 1 | Vvi-Vitvi08g01744\_t001 |  | \| \| |  |  |  |  |  |  |  |  |  |  |
| 1 | Vvi-Vitvi08g02347\_t001 |  | \| \| |  |  |  |  |  |  |  |  |  |  |
| 1 | Vvi-Vitvi08g04397\_t001 |  | \| \| |  |  |  |  |  |  |  |  |  |  |
| 1 | Vvi-Vitvi08g04398\_t001 |  | \| \| |  |  |  |  |  |  |  |  |  |  |
| 1 | Vvi-Vitvi08g01746\_t001 |  | \| \| |  |  |  |  |  |  |  |  |  |  |
| 1 | Vvi-Vitvi08g04399\_t001 |  | \| \| |  |  |  |  |  |  |  |  |  |  |
| 1 | Vvi-Vitvi08g01747\_t001 |  | \| \| |  |  |  |  |  |  |  |  |  |  |
| 1 | Vvi-Vitvi08g02349\_t001 |  | Atr-ERN17071 |  |  |  |  |  |  |  |  |  |  |
| 0 | Vvi-Vitvi08g02350\_t001 |  |  |  |  |  |  |  |  |  |  |  |  |
| 0 | Vvi-Vitvi08g01748\_t001 |  |  |  |  |  |  |  |  |  |  |  |  |
| 1 | Vvi-Vitvi08g01749\_t001 |  | Atr-ERN11115 |  |  |  |  |  |  |  |  |  |  |
| 1 | Vvi-Vitvi08g01750\_t001 |  | \| \| |  |  |  |  |  |  |  |  |  |  |
| 1 | Vvi-Vitvi08g01751\_t001 |  | Atr-ERN11121 |  |  |  |  |  |  |  |  |  |  |
| 1 | Vvi-Vitvi08g01752\_t001 |  | \| \| |  |  |  |  |  |  |  |  |  |  |
| 1 | Vvi-Vitvi08g01753\_t001 |  | \| \| |  |  |  |  |  |  |  |  |  |  |
| 1 | Vvi-Vitvi08g02352\_t002 |  | \| \| |  |  |  |  |  |  |  |  |  |  |
| 1 | Vvi-Vitvi08g01756\_t001 |  | Atr-ERN11126 |  |  |  |  |  |  |  |  |  |  |
| 1 | Vvi-Vitvi08g01757\_t001 |  | Atr-ERN11128 |  |  |  |  |  |  |  |  |  |  |
| 1 | Vvi-Vitvi08g01758\_t001 |  | Atr-ERN11129 |  |  |  |  |  |  |  |  |  |  |
| 1 | Vvi-Vitvi08g01759\_t001 |  | Atr-ERN11130 |  |  |  |  |  |  |  |  |  |  |
| 1 | Vvi-Vitvi08g04400\_t001 |  | \| \| |  |  |  |  |  |  |  |  |  |  |
| 1 | Vvi-Vitvi08g01760\_t001 |  | \| \| |  |  |  |  |  |  |  |  |  |  |
| 1 | Vvi-Vitvi08g01761\_t001 |  | \| \| |  |  |  |  |  |  |  |  |  |  |
| 1 | Vvi-Vitvi08g04401\_t001 |  | \| \| |  |  |  |  |  |  |  |  |  |  |
| 1 | Vvi-Vitvi08g01762\_t001 |  | Atr-ERN11133 |  |  |  |  |  |  |  |  |  |  |
| 1 | Vvi-Vitvi08g01764\_t001 |  | Atr-ERN11137 |  |  |  |  |  |  |  |  |  |  |
| 1 | Vvi-Vitvi08g01765\_t001 |  | \| \| |  |  |  |  |  |  |  |  |  |  |
| 1 | Vvi-Vitvi08g01766\_t001 |  | \| \| |  |  |  |  |  |  |  |  |  |  |
| 1 | Vvi-Vitvi08g01767\_t001 |  | \| \| |  |  |  |  |  |  |  |  |  |  |
| 1 | Vvi-Vitvi08g04402\_t001 |  | \| \| |  |  |  |  |  |  |  |  |  |  |
| 1 | Vvi-Vitvi08g04403\_t001 |  | \| \| |  |  |  |  |  |  |  |  |  |  |
| 1 | Vvi-Vitvi08g01768\_t001 |  | \| \| |  |  |  |  |  |  |  |  |  |  |
| 1 | Vvi-Vitvi08g04404\_t001 |  | \| \| |  |  |  |  |  |  |  |  |  |  |
| 1 | Vvi-Vitvi08g01769\_t001 |  | Atr-ERN11151 |  |  |  |  |  |  |  |  |  |  |
| 1 | Vvi-Vitvi08g04405\_t001 |  | \| \| |  |  |  |  |  |  |  |  |  |  |
| 1 | Vvi-Vitvi08g01770\_t001 |  | \| \| |  |  |  |  |  |  |  |  |  |  |
| 1 | Vvi-Vitvi08g01771\_t001 |  | Atr-ERN11152 |  |  |  |  |  |  |  |  |  |  |
| 0 | Vvi-Vitvi08g01772\_t002 |  |  |  |  |  |  |  |  |  |  |  |  |
| 0 | Vvi-Vitvi08g01773\_t001 |  |  |  |  |  |  |  |  |  |  |  |  |
| 0 | Vvi-Vitvi08g01774\_t001 |  |  |  |  |  |  |  |  |  |  |  |  |
| 0 | Vvi-Vitvi08g02355\_t001 |  |  |  |  |  |  |  |  |  |  |  |  |
| 0 | Vvi-Vitvi08g02357\_t001 |  |  |  |  |  |  |  |  |  |  |  |  |
| 0 | Vvi-Vitvi08g02358\_t001 |  |  |  |  |  |  |  |  |  |  |  |  |
| 0 | Vvi-Vitvi08g02359\_t001 |  |  |  |  |  |  |  |  |  |  |  |  |
| 0 | Vvi-Vitvi08g04406\_t001 |  |  |  |  |  |  |  |  |  |  |  |  |
| 0 | Vvi-Vitvi08g02360\_t001 |  |  |  |  |  |  |  |  |  |  |  |  |
| 0 | Vvi-Vitvi08g04407\_t001 |  |  |  |  |  |  |  |  |  |  |  |  |
| 0 | Vvi-Vitvi08g01776\_t001 |  |  |  |  |  |  |  |  |  |  |  |  |
| 0 | Vvi-Vitvi08g04408\_t001 |  |  |  |  |  |  |  |  |  |  |  |  |
| 0 | Vvi-Vitvi08g01777\_t001 |  |  |  |  |  |  |  |  |  |  |  |  |
| 0 | Vvi-Vitvi08g01778\_t001 |  |  |  |  |  |  |  |  |  |  |  |  |
| 0 | Vvi-Vitvi08g02363\_t004 |  |  |  |  |  |  |  |  |  |  |  |  |
| 1 | Vvi-Vitvi08g01779\_t002 |  | Atr-ERN04439 |  |  |  |  |  |  |  |  |  |  |
| 1 | Vvi-Vitvi08g01780\_t001 |  | Atr-ERN04442 |  |  |  |  |  |  |  |  |  |  |
| 1 | Vvi-Vitvi08g01781\_t001 |  | \| \| |  |  |  |  |  |  |  |  |  |  |
| 1 | Vvi-Vitvi08g01782\_t001 |  | Atr-ERN04445 |  |  |  |  |  |  |  |  |  |  |
| 1 | Vvi-Vitvi08g01783\_t001 |  | Atr-ERN04447 |  |  |  |  |  |  |  |  |  |  |
| 1 | Vvi-Vitvi08g01784\_t001 |  | Atr-ERN04448 |  |  |  |  |  |  |  |  |  |  |
| 1 | Vvi-Vitvi08g01786\_t001 |  | Atr-ERN04452 |  |  |  |  |  |  |  |  |  |  |
| 1 | Vvi-Vitvi08g02366\_t001 |  | Atr-ERN04458 |  |  |  |  |  |  |  |  |  |  |
| 0 | Vvi-Vitvi08g01787\_t001 |  |  |  |  |  |  |  |  |  |  |  |  |
| 1 | Vvi-Vitvi08g01788\_t001 |  | Atr-ERN20462 |  |  |  |  |  |  |  |  |  |  |
| 1 | Vvi-Vitvi08g01789\_t001 |  | \| \| |  |  |  |  |  |  |  |  |  |  |
| 1 | Vvi-Vitvi08g01790\_t001 |  | \| \| |  |  |  |  |  |  |  |  |  |  |
| 1 | Vvi-Vitvi08g01791\_t002 |  | \| \| |  |  |  |  |  |  |  |  |  |  |
| 1 | Vvi-Vitvi08g01792\_t002 |  | \| \| |  |  |  |  |  |  |  |  |  |  |
| 1 | Vvi-Vitvi08g01793\_t003 |  | \| \| |  |  |  |  |  |  |  |  |  |  |
| 1 | Vvi-Vitvi08g01794\_t001 |  | \| \| |  |  |  |  |  |  |  |  |  |  |
| 1 | Vvi-Vitvi08g04409\_t001 |  | \| \| |  |  |  |  |  |  |  |  |  |  |
| 1 | Vvi-Vitvi08g01795\_t001 |  | \| \| |  |  |  |  |  |  |  |  |  |  |
| 1 | Vvi-Vitvi08g02367\_t001 |  | \| \| |  |  |  |  |  |  |  |  |  |  |
| 1 | Vvi-Vitvi08g04410\_t001 |  | \| \| |  |  |  |  |  |  |  |  |  |  |
| 1 | Vvi-Vitvi08g01796\_t002 |  | Atr-ERN20441 |  |  |  |  |  |  |  |  |  |  |
| 1 | Vvi-Vitvi08g01797\_t001 |  | Atr-ERN20440 |  |  |  |  |  |  |  |  |  |  |
| 1 | Vvi-Vitvi08g01798\_t001 |  | Atr-ERN20418 |  |  |  |  |  |  |  |  |  |  |
| 1 | Vvi-Vitvi08g01965\_t001 |  | Atr-ERN20416 |  |  |  |  |  |  |  |  |  |  |
| 1 | Vvi-Vitvi08g01800\_t001 |  | Atr-ERN20411 |  |  |  |  |  |  |  |  |  |  |
| 1 | Vvi-Vitvi08g04411\_t001 |  | \| \| |  |  |  |  |  |  |  |  |  |  |
| 1 | Vvi-Vitvi08g01802\_t001 |  | Atr-ERN20405 |  |  |  |  |  |  |  |  |  |  |
| 1 | Vvi-Vitvi08g01803\_t001 |  | Atr-ERN20404 |  |  |  |  |  |  |  |  |  |  |
| 1 | Vvi-Vitvi08g01804\_t001 |  | Atr-ERN20398 |  |  |  |  |  |  |  |  |  |  |
| 1 | Vvi-Vitvi08g01805\_t001 |  | Atr-ERN20396 |  |  |  |  |  |  |  |  |  |  |
| 1 | Vvi-Vitvi08g02368\_t001 |  | \| \| |  |  |  |  |  |  |  |  |  |  |
| 1 | Vvi-Vitvi08g01807\_t001 |  | Atr-ERN20394 |  |  |  |  |  |  |  |  |  |  |
| 1 | Vvi-Vitvi08g01808\_t002 |  | Atr-ERN20393 |  |  |  |  |  |  |  |  |  |  |
| 1 | Vvi-Vitvi08g02369\_t001 |  | Atr-ERN20389 |  |  |  |  |  |  |  |  |  |  |
| 1 | Vvi-Vitvi08g01809\_t001 |  | \| \| |  |  |  |  |  |  |  |  |  |  |
| 1 | Vvi-Vitvi08g01810\_t001 |  | Atr-ERN20385 |  |  |  |  |  |  |  |  |  |  |
| 1 | Vvi-Vitvi08g01811\_t001 |  | Atr-ERN20384 |  |  |  |  |  |  |  |  |  |  |
| 1 | Vvi-Vitvi08g01812\_t002 |  | Atr-ERM93524 |  |  |  |  |  |  |  |  |  |  |
| 1 | Vvi-Vitvi08g01813\_t001 |  | Atr-ERM93526 |  |  |  |  |  |  |  |  |  |  |
| 1 | Vvi-Vitvi08g01814\_t001 |  | \| \| |  |  |  |  |  |  |  |  |  |  |
| 1 | Vvi-Vitvi08g01815\_t001 |  | Atr-ERM93531 |  |  |  |  |  |  |  |  |  |  |
| 1 | Vvi-Vitvi08g01816\_t001 |  | Atr-ERM93533 |  |  |  |  |  |  |  |  |  |  |
| 1 | Vvi-Vitvi08g01817\_t003 |  | Atr-ERM93538 |  |  |  |  |  |  |  |  |  |  |
| 2 | Vvi-Vitvi08g01818\_t001 |  | \| \| |  | Atr-ERM93549 |  |  |  |  |  |  |  |  |
| 2 | Vvi-Vitvi08g04412\_t001 |  | \| \| |  | \| \| |  |  |  |  |  |  |  |  |
| 2 | Vvi-Vitvi08g01820\_t001 |  | \| \| |  | Atr-ERM93547 |  |  |  |  |  |  |  |  |
| 2 | Vvi-Vitvi08g01821\_t001 |  | \| \| |  | \| \| |  |  |  |  |  |  |  |  |
| 2 | Vvi-Vitvi08g01822\_t001 |  | \| \| |  | Atr-ERM93546 |  |  |  |  |  |  |  |  |
| 2 | Vvi-Vitvi08g01824\_t001 |  | Atr-ERM93545 |  | \| \| |  |  |  |  |  |  |  |  |
| 2 | Vvi-Vitvi08g01825\_t001 |  | \| \| |  | Atr-ERM93543 |  |  |  |  |  |  |  |  |
| 2 | Vvi-Vitvi08g01826\_t001 |  | \| \| |  | Atr-ERM93542 |  |  |  |  |  |  |  |  |
| 2 | Vvi-Vitvi08g01828\_t001 |  | \| \| |  | \| \| |  |  |  |  |  |  |  |  |
| 2 | Vvi-Vitvi08g01829\_t001 |  | \| \| |  | Atr-ERM93540 |  |  |  |  |  |  |  |  |
| 2 | Vvi-Vitvi08g01831\_t001 |  | \| \| |  | Atr-ERM93539 |  |  |  |  |  |  |  |  |
| 1 | Vvi-Vitvi08g01832\_t001 |  | Atr-ERM93550 |  |  |  |  |  |  |  |  |  |  |
| 1 | Vvi-Vitvi08g01833\_t004 |  | Atr-ERM93560 |  |  |  |  |  |  |  |  |  |  |
| 0 | Vvi-Vitvi08g01834\_t001 |  |  |  |  |  |  |  |  |  |  |  |  |
| 0 | Vvi-Vitvi08g01835\_t001 |  |  |  |  |  |  |  |  |  |  |  |  |
| 0 | Vvi-Vitvi08g01836\_t001 |  |  |  |  |  |  |  |  |  |  |  |  |
| 0 | Vvi-Vitvi08g01837\_t001 |  |  |  |  |  |  |  |  |  |  |  |  |
| 0 | Vvi-Vitvi08g01838\_t001 |  |  |  |  |  |  |  |  |  |  |  |  |
| 0 | Vvi-Vitvi08g01839\_t001 |  |  |  |  |  |  |  |  |  |  |  |  |
| 0 | Vvi-Vitvi08g01841\_t001 |  |  |  |  |  |  |  |  |  |  |  |  |
| 0 | Vvi-Vitvi08g02370\_t001 |  |  |  |  |  |  |  |  |  |  |  |  |
| 0 | Vvi-Vitvi08g01842\_t001 |  |  |  |  |  |  |  |  |  |  |  |  |
| 0 | Vvi-Vitvi08g01843\_t001 |  |  |  |  |  |  |  |  |  |  |  |  |
| 0 | Vvi-Vitvi08g02371\_t001 |  |  |  |  |  |  |  |  |  |  |  |  |
| 1 | Vvi-Vitvi08g01844\_t001 |  | Atr-ERN11670 |  |  |  |  |  |  |  |  |  |  |
| 1 | Vvi-Vitvi08g01845\_t001 |  | Atr-ERN11681 |  |  |  |  |  |  |  |  |  |  |
| 1 | Vvi-Vitvi08g02372\_t001 |  | Atr-ERN11688 |  |  |  |  |  |  |  |  |  |  |
| 1 | Vvi-Vitvi08g01846\_t001 |  | \| \| |  |  |  |  |  |  |  |  |  |  |
| 1 | Vvi-Vitvi08g01847\_t001 |  | \| \| |  |  |  |  |  |  |  |  |  |  |
| 1 | Vvi-Vitvi08g01848\_t001 |  | \| \| |  |  |  |  |  |  |  |  |  |  |
| 1 | Vvi-Vitvi08g04413\_t001 |  | \| \| |  |  |  |  |  |  |  |  |  |  |
| 1 | Vvi-Vitvi08g02373\_t001 |  | \| \| |  |  |  |  |  |  |  |  |  |  |
| 1 | Vvi-Vitvi08g02374\_t001 |  | \| \| |  |  |  |  |  |  |  |  |  |  |
| 1 | Vvi-Vitvi08g02375\_t001 |  | \| \| |  |  |  |  |  |  |  |  |  |  |
| 1 | Vvi-Vitvi08g01851\_t001 |  | \| \| |  |  |  |  |  |  |  |  |  |  |
| 1 | Vvi-Vitvi08g02377\_t001 |  | Atr-ERN11700 |  |  |  |  |  |  |  |  |  |  |
| 1 | Vvi-Vitvi08g01852\_t001 |  | Atr-ERN11704 |  |  |  |  |  |  |  |  |  |  |
| 1 | Vvi-Vitvi08g01853\_t001 |  | Atr-ERN11706 |  |  |  |  |  |  |  |  |  |  |
| 1 | Vvi-Vitvi08g01854\_t001 |  | Atr-ERN11707 |  |  |  |  |  |  |  |  |  |  |
| 1 | Vvi-Vitvi08g01855\_t001 |  | \| \| |  |  |  |  |  |  |  |  |  |  |
| 1 | Vvi-Vitvi08g02378\_t004 |  | Atr-ERN11710 |  |  |  |  |  |  |  |  |  |  |
| 1 | Vvi-Vitvi08g01856\_t001 |  | Atr-ERN11713 |  |  |  |  |  |  |  |  |  |  |
| 1 | Vvi-Vitvi08g01857\_t001 |  | Atr-ERN11715 |  |  |  |  |  |  |  |  |  |  |
| 1 | Vvi-Vitvi08g01858\_t001 |  | \| \| |  |  |  |  |  |  |  |  |  |  |
| 1 | Vvi-Vitvi08g01859\_t001 |  | Atr-ERN11718 |  |  |  |  |  |  |  |  |  |  |
| 1 | Vvi-Vitvi08g01860\_t003 |  | Atr-ERN11719 |  |  |  |  |  |  |  |  |  |  |
| 1 | Vvi-Vitvi08g01861\_t001 |  | \| \| |  |  |  |  |  |  |  |  |  |  |
| 1 | Vvi-Vitvi08g01863\_t001 |  | Atr-ERN11736 |  |  |  |  |  |  |  |  |  |  |
| 1 | Vvi-Vitvi08g04414\_t001 |  | \| \| |  |  |  |  |  |  |  |  |  |  |
| 1 | Vvi-Vitvi08g01864\_t001 |  | Atr-ERN11738 |  |  |  |  |  |  |  |  |  |  |
| 1 | Vvi-Vitvi08g04415\_t001 |  | \| \| |  |  |  |  |  |  |  |  |  |  |
| 1 | Vvi-Vitvi08g01865\_t001 |  | \| \| |  |  |  |  |  |  |  |  |  |  |
| 1 | Vvi-Vitvi08g01866\_t002 |  | Atr-ERN11744 |  |  |  |  |  |  |  |  |  |  |
| 1 | Vvi-Vitvi08g02381\_t001 |  | \| \| |  |  |  |  |  |  |  |  |  |  |
| 1 | Vvi-Vitvi08g04416\_t001 |  | \| \| |  |  |  |  |  |  |  |  |  |  |
| 1 | Vvi-Vitvi08g04417\_t001 |  | \| \| |  |  |  |  |  |  |  |  |  |  |
| 1 | Vvi-Vitvi08g02382\_t001 |  | \| \| |  |  |  |  |  |  |  |  |  |  |
| 1 | Vvi-Vitvi08g02383\_t001 |  | \| \| |  |  |  |  |  |  |  |  |  |  |
| 1 | Vvi-Vitvi08g04418\_t001 |  | \| \| |  |  |  |  |  |  |  |  |  |  |
| 1 | Vvi-Vitvi08g04419\_t001 |  | \| \| |  |  |  |  |  |  |  |  |  |  |
| 1 | Vvi-Vitvi08g04420\_t001 |  | \| \| |  |  |  |  |  |  |  |  |  |  |
| 1 | Vvi-Vitvi08g04421\_t001 |  | \| \| |  |  |  |  |  |  |  |  |  |  |
| 1 | Vvi-Vitvi08g02386\_t001 |  | \| \| |  |  |  |  |  |  |  |  |  |  |
| 1 | Vvi-Vitvi08g02387\_t001 |  | \| \| |  |  |  |  |  |  |  |  |  |  |
| 1 | Vvi-Vitvi08g01867\_t001 |  | Atr-ERN11752 |  |  |  |  |  |  |  |  |  |  |
| 1 | Vvi-Vitvi08g01868\_t001 |  | \| \| |  |  |  |  |  |  |  |  |  |  |
| 1 | Vvi-Vitvi08g01869\_t001 |  | Atr-ERN11757 |  |  |  |  |  |  |  |  |  |  |
| 1 | Vvi-Vitvi08g01870\_t002 |  | \| \| |  |  |  |  |  |  |  |  |  |  |
| 1 | Vvi-Vitvi08g01871\_t001 |  | Atr-ERN11762 |  |  |  |  |  |  |  |  |  |  |
| 1 | Vvi-Vitvi08g04422\_t001 |  | \| \| |  |  |  |  |  |  |  |  |  |  |
| 1 | Vvi-Vitvi08g02389\_t001 |  | \| \| |  |  |  |  |  |  |  |  |  |  |
| 1 | Vvi-Vitvi08g01872\_t001 |  | \| \| |  |  |  |  |  |  |  |  |  |  |
| 1 | Vvi-Vitvi08g01873\_t001 |  | Atr-ERN11776 |  |  |  |  |  |  |  |  |  |  |
| 1 | Vvi-Vitvi08g01874\_t001 |  | \| \| |  |  |  |  |  |  |  |  |  |  |
| 1 | Vvi-Vitvi08g02390\_t001 |  | Atr-ERN11784 |  |  |  |  |  |  |  |  |  |  |
| 1 | Vvi-Vitvi08g04423\_t001 |  | \| \| |  |  |  |  |  |  |  |  |  |  |
| 1 | Vvi-Vitvi08g04424\_t001 |  | \| \| |  |  |  |  |  |  |  |  |  |  |
| 1 | Vvi-Vitvi08g01966\_t001 |  | \| \| |  |  |  |  |  |  |  |  |  |  |
| 1 | Vvi-Vitvi08g01875\_t001 |  | \| \| |  |  |  |  |  |  |  |  |  |  |
| 1 | Vvi-Vitvi08g02391\_t001 |  | \| \| |  |  |  |  |  |  |  |  |  |  |
| 1 | Vvi-Vitvi08g01876\_t002 |  | Atr-ERN11790 |  |  |  |  |  |  |  |  |  |  |
| 1 | Vvi-Vitvi08g01878\_t001 |  | Atr-ERN11795 |  |  |  |  |  |  |  |  |  |  |
| 1 | Vvi-Vitvi08g01879\_t001 |  | Atr-ERN11797 |  |  |  |  |  |  |  |  |  |  |
| 1 | Vvi-Vitvi08g01880\_t002 |  | Atr-ERN00916 |  |  |  |  |  |  |  |  |  |  |
| 2 | Vvi-Vitvi08g01881\_t001 |  | \| \| |  | Atr-ERN00882 |  |  |  |  |  |  |  |  |
| 2 | Vvi-Vitvi08g02393\_t001 |  | \| \| |  | Atr-ERN00890 |  |  |  |  |  |  |  |  |
| 2 | Vvi-Vitvi08g01883\_t002 |  | Atr-ERN00900 |  | Atr-ERN00900 |  |  |  |  |  |  |  |  |
| 2 | Vvi-Vitvi08g01884\_t001 |  | Atr-ERN00899 |  | \| \| |  |  |  |  |  |  |  |  |
| 2 | Vvi-Vitvi08g01886\_t001 |  | \| \| |  | \| \| |  |  |  |  |  |  |  |  |
| 2 | Vvi-Vitvi08g01887\_t001 |  | Atr-ERN00893 |  | \| \| |  |  |  |  |  |  |  |  |
| 2 | Vvi-Vitvi08g01888\_t001 |  | \| \| |  | Atr-ERN00904 |  |  |  |  |  |  |  |  |
| 2 | Vvi-Vitvi08g01889\_t002 |  | \| \| |  | Atr-ERN00909 |  |  |  |  |  |  |  |  |
| 2 | Vvi-Vitvi08g01890\_t003 |  | \| \| |  | Atr-ERN00911 |  |  |  |  |  |  |  |  |
| 1 | Vvi-Vitvi08g01891\_t001 |  | \| \| |  |  |  |  |  |  |  |  |  |  |
| 1 | Vvi-Vitvi08g02394\_t001 |  | Atr-ERN00872 |  |  |  |  |  |  |  |  |  |  |
| 1 | Vvi-Vitvi08g01893\_t001 |  | \| \| |  |  |  |  |  |  |  |  |  |  |
| 1 | Vvi-Vitvi08g01894\_t001 |  | \| \| |  |  |  |  |  |  |  |  |  |  |
| 1 | Vvi-Vitvi08g01895\_t001 |  | Atr-ERN00865 |  |  |  |  |  |  |  |  |  |  |
| 1 | Vvi-Vitvi08g01896\_t001 |  | \| \| |  |  |  |  |  |  |  |  |  |  |
| 1 | Vvi-Vitvi08g02395\_t001 |  | \| \| |  |  |  |  |  |  |  |  |  |  |
| 1 | Vvi-Vitvi08g01897\_t001 |  | \| \| |  |  |  |  |  |  |  |  |  |  |
| 1 | Vvi-Vitvi08g04425\_t001 |  | \| \| |  |  |  |  |  |  |  |  |  |  |
| 1 | Vvi-Vitvi08g01899\_t001 |  | \| \| |  |  |  |  |  |  |  |  |  |  |
| 1 | Vvi-Vitvi08g01900\_t001 |  | \| \| |  |  |  |  |  |  |  |  |  |  |
| 1 | Vvi-Vitvi08g02397\_t001 |  | \| \| |  |  |  |  |  |  |  |  |  |  |
| 1 | Vvi-Vitvi08g01901\_t001 |  | Atr-ERN00851 |  |  |  |  |  |  |  |  |  |  |
| 1 | Vvi-Vitvi08g02398\_t001 |  | \| \| |  |  |  |  |  |  |  |  |  |  |
| 1 | Vvi-Vitvi08g01902\_t001 |  | \| \| |  |  |  |  |  |  |  |  |  |  |
| 1 | Vvi-Vitvi08g04426\_t001 |  | \| \| |  |  |  |  |  |  |  |  |  |  |
| 1 | Vvi-Vitvi08g01904\_t001 |  | \| \| |  |  |  |  |  |  |  |  |  |  |
| 1 | Vvi-Vitvi08g01905\_t002 |  | Atr-ERN00848 |  |  |  |  |  |  |  |  |  |  |
| 1 | Vvi-Vitvi08g01906\_t001 |  | Atr-ERN00845 |  |  |  |  |  |  |  |  |  |  |
| 0 | Vvi-Vitvi08g01910\_t001 |  |  |  |  |  |  |  |  |  |  |  |  |
| 0 | Vvi-Vitvi08g01911\_t001 |  |  |  |  |  |  |  |  |  |  |  |  |
| 0 | Vvi-Vitvi08g01912\_t001 |  |  |  |  |  |  |  |  |  |  |  |  |
| 0 | Vvi-Vitvi08g04427\_t001 |  |  |  |  |  |  |  |  |  |  |  |  |
| 0 | Vvi-Vitvi08g02401\_t001 |  |  |  |  |  |  |  |  |  |  |  |  |
| 0 | Vvi-Vitvi08g01913\_t001 |  |  |  |  |  |  |  |  |  |  |  |  |
| 0 | Vvi-Vitvi08g01914\_t001 |  |  |  |  |  |  |  |  |  |  |  |  |
| 0 | Vvi-Vitvi08g01915\_t001 |  |  |  |  |  |  |  |  |  |  |  |  |
| 0 | Vvi-Vitvi08g01916\_t002 |  |  |  |  |  |  |  |  |  |  |  |  |
| 0 | Vvi-Vitvi08g01917\_t001 |  |  |  |  |  |  |  |  |  |  |  |  |
| 0 | Vvi-Vitvi08g01918\_t001 |  |  |  |  |  |  |  |  |  |  |  |  |
| 0 | Vvi-Vitvi08g01919\_t001 |  |  |  |  |  |  |  |  |  |  |  |  |
| 0 | Vvi-Vitvi08g01920\_t001 |  |  |  |  |  |  |  |  |  |  |  |  |
| 0 | Vvi-Vitvi08g04428\_t001 |  |  |  |  |  |  |  |  |  |  |  |  |
| 0 | Vvi-Vitvi08g01922\_t001 |  |  |  |  |  |  |  |  |  |  |  |  |
| 0 | Vvi-Vitvi08g02406\_t001 |  |  |  |  |  |  |  |  |  |  |  |  |
| 0 | Vvi-Vitvi08g01923\_t001 |  |  |  |  |  |  |  |  |  |  |  |  |
| 0 | Vvi-Vitvi08g01924\_t001 |  |  |  |  |  |  |  |  |  |  |  |  |
| 0 | Vvi-Vitvi08g01925\_t001 |  |  |  |  |  |  |  |  |  |  |  |  |
| 0 | Vvi-Vitvi08g01926\_t001 |  |  |  |  |  |  |  |  |  |  |  |  |
| 0 | Vvi-Vitvi08g01927\_t001 |  |  |  |  |  |  |  |  |  |  |  |  |
| 0 | Vvi-Vitvi08g01929\_t001 |  |  |  |  |  |  |  |  |  |  |  |  |
| 0 | Vvi-Vitvi08g01931\_t001 |  |  |  |  |  |  |  |  |  |  |  |  |
| 0 | Vvi-Vitvi08g01932\_t001 |  |  |  |  |  |  |  |  |  |  |  |  |
| 0 | Vvi-Vitvi08g01933\_t001 |  |  |  |  |  |  |  |  |  |  |  |  |
| 1 | Vvi-Vitvi08g01934\_t001 |  | Atr-ERN05183 |  |  |  |  |  |  |  |  |  |  |
| 1 | Vvi-Vitvi08g01935\_t001 |  | \| \| |  |  |  |  |  |  |  |  |  |  |
| 1 | Vvi-Vitvi08g01936\_t001 |  | \| \| |  |  |  |  |  |  |  |  |  |  |
| 1 | Vvi-Vitvi08g01938\_t001 |  | \| \| |  |  |  |  |  |  |  |  |  |  |
| 1 | Vvi-Vitvi08g01939\_t001 |  | \| \| |  |  |  |  |  |  |  |  |  |  |
| 1 | Vvi-Vitvi08g01940\_t001 |  | \| \| |  |  |  |  |  |  |  |  |  |  |
| 1 | Vvi-Vitvi08g01943\_t001 |  | \| \| |  |  |  |  |  |  |  |  |  |  |
| 1 | Vvi-Vitvi08g02408\_t001 |  | \| \| |  |  |  |  |  |  |  |  |  |  |
| 1 | Vvi-Vitvi08g02410\_t001 |  | \| \| |  |  |  |  |  |  |  |  |  |  |
| 1 | Vvi-Vitvi08g04429\_t001 |  | \| \| |  |  |  |  |  |  |  |  |  |  |
| 1 | Vvi-Vitvi08g02411\_t001 |  | \| \| |  |  |  |  |  |  |  |  |  |  |
| 1 | Vvi-Vitvi08g02412\_t001 |  | \| \| |  |  |  |  |  |  |  |  |  |  |
| 1 | Vvi-Vitvi08g01944\_t001 |  | \| \| |  |  |  |  |  |  |  |  |  |  |
| 1 | Vvi-Vitvi08g04430\_t001 |  | \| \| |  |  |  |  |  |  |  |  |  |  |
| 1 | Vvi-Vitvi08g01945\_t001 |  | \| \| |  |  |  |  |  |  |  |  |  |  |
| 1 | Vvi-Vitvi08g01946\_t001 |  | \| \| |  |  |  |  |  |  |  |  |  |  |
| 1 | Vvi-Vitvi08g01947\_t001 |  | \| \| |  |  |  |  |  |  |  |  |  |  |
| 1 | Vvi-Vitvi08g02413\_t001 |  | Atr-ERN05168 |  |  |  |  |  |  |  |  |  |  |
| 1 | Vvi-Vitvi08g01949\_t001 |  | Atr-ERN05166 |  |  |  |  |  |  |  |  |  |  |
| 1 | Vvi-Vitvi08g04431\_t001 |  | \| \| |  |  |  |  |  |  |  |  |  |  |
| 1 | Vvi-Vitvi08g02414\_t001 |  | \| \| |  |  |  |  |  |  |  |  |  |  |
| 1 | Vvi-Vitvi08g02415\_t001 |  | Atr-ERN05165 |  |  |  |  |  |  |  |  |  |  |
| 1 | Vvi-Vitvi08g01950\_t001 |  | \| \| |  |  |  |  |  |  |  |  |  |  |
| 1 | Vvi-Vitvi08g04432\_t001 |  | \| \| |  |  |  |  |  |  |  |  |  |  |
| 1 | Vvi-Vitvi08g01951\_t002 |  | \| \| |  |  |  |  |  |  |  |  |  |  |
| 1 | Vvi-Vitvi08g01952\_t001 |  | \| \| |  |  |  |  |  |  |  |  |  |  |
| 1 | Vvi-Vitvi08g01953\_t001 |  | \| \| |  |  |  |  |  |  |  |  |  |  |
| 1 | Vvi-Vitvi08g01964\_t001 |  | \| \| |  |  |  |  |  |  |  |  |  |  |
| 1 | Vvi-Vitvi08g01963\_t001 |  | \| \| |  |  |  |  |  |  |  |  |  |  |
| 1 | Vvi-Vitvi08g02419\_t001 |  | \| \| |  |  |  |  |  |  |  |  |  |  |
| 1 | Vvi-Vitvi08g01962\_t001 |  | Atr-ERN05161 |  |  |  |  |  |  |  |  |  |  |
| 1 | Vvi-Vitvi08g01960\_t001 |  | \| \| |  |  |  |  |  |  |  |  |  |  |
| 1 | Vvi-Vitvi08g01959\_t001 |  | Atr-ERN05158 |  |  |  |  |  |  |  |  |  |  |
| 1 | Vvi-Vitvi08g01958\_t001 |  | \| \| |  |  |  |  |  |  |  |  |  |  |
| 1 | Vvi-Vitvi08g02418\_t001 |  | \| \| |  |  |  |  |  |  |  |  |  |  |
| 1 | Vvi-Vitvi08g04433\_t001 |  | \| \| |  |  |  |  |  |  |  |  |  |  |
| 1 | Vvi-Vitvi08g01957\_t001 |  | Atr-ERN05150 |  |  |  |  |  |  |  |  |  |  |
| 1 | Vvi-Vitvi08g01955\_t001 |  | Atr-ERN05149 |  |  |  |  |  |  |  |  |  |  |
